# Longitudinal effect of early social media use on standardized learning outcomes during school career

**DOI:** 10.1038/s41562-026-02522-4

**Published:** 2026-08-03

**Authors:** Marco Gui, Chiara Respi, Giovanni Abbiati, Cristiana Paladini, Sofia Ercolanoni, Francesca Milzani, Tiziana Pirola, Giovanni Vezzoli

**Affiliations:** 1 Department of Sociology and Social Research, University of Milano-Bicocca, Milan, Italy; 2 Department of Economics and Management, University of Brescia, Brescia, Italy; 3 Centro Studi Socialis, Brescia, Italy

**Keywords:** Sociology, Education

## Abstract

This study uses difference-in-differences models to evaluate the impact of early social media use on academic performance among children aged 7–16 years. A dataset comprising 5,227 students from northern Italy was constructed by merging data from a questionnaire on lifetime digital media habits with longitudinal standardized test scores in Italian, English and mathematics. Participants who created a personal social media account in 6th, 7th or 8th grade were separately matched with late adopters who initiated their first social media account in the 9th grade or later. The findings provide compelling evidence that early engagement with social media adversely affects learning outcomes. Additional findings on the effects of early social media use on other academic outcomes for which we do not have longitudinal data are in line with our main findings. We also find supporting evidence that a substantial part of this negative effect is mediated by students’ smartphone pervasiveness in key moments of the day.

## Abstract (web summary)

Analysing data from 5,227 students from northern Italy, this article finds causal evidence that early engagement with social media adversely affects learning outcomes.

## Main

There is a heated debate among scholars and in the media about children’s early access to digital devices^1,2^. Amid conflicting expert recommendations^3,4^, parents and educators seem confused and uncertain^5^. The average moment personal smartphones are acquired has been brought forward among early adolescents aged 10–11 years, and the COVID-19 (coronavirus disease 2019) pandemic further accelerated this trend^6,7^. At the same age, the majority of young people create their first personal social media account^8,9^, often below the platforms’ (or country’s) minimum age requirements (13 years old in the United States and between 14 and 16 years old in Europe for autonomous use). Concerns are further supported by data showing that as minors’ engagement with digital media has increased, several mental health indicators (with the exception of suicides) have declined in many countries^10–12^, and academic performance has also deteriorated on a global level^13,14^.

Social media have been particularly scrutinized by scholars and institutions as risk factors for the well-functioning of minors, with the majority of studies considering psychological well-being as the dependent variable^2,15–17^. Most studies suggest that intensive social media use is associated with negative well-being outcomes, including poorer sleep quality^18–21^, reduced concentration levels^22,23^ and decreased face-to-face interactions, which, in turn, may lower life satisfaction^19,24,25^. Prominent popular science books have argued that social media use, experienced pervasively through a personal smartphone, is a significant contributing factor to mental health issues, at least for a substantial minority of adolescent users^2,26^. However, other scholars have highlighted that the relationship remains unclear owing to conflicting findings, evidence of counter-causality and methodological limitations^17,27–29^. Contrasting perspectives regarding the causal interpretation of findings and their substantive relevance persist^30,31^.

A smaller number of studies have considered the relationship between social media use during leisure time and learning outcomes. A number of meta-analyses found evidence of minor negative correlations^32–34^. Most of the individual studies were conducted on university students and consider the quantity of social media use (for example, time spent and frequency) as the main independent variable^35–37^. Similar findings have been observed in a few studies among lower- and upper-secondary school students^38^. Here too, evidence is often criticized as based on correlational or short-term longitudinal data^1,39,40^.

Another limitation of this field of literature is the lack of substantial evidence regarding the effects of the age at which individuals begin using social media. This is paradoxical given the intense public debate surrounding the issue^41,42^. Growing numbers of parents are campaigning for a delay in the access to smartphones and social media (for example, ‘Wait until 8th’ and ‘Mothers against Media Addiction’ in the United States, ‘Smartphone Free Childhood’ in the United Kingdom and ‘Patti Digitali’ in Italy). Although a few studies have examined the learning impact of early smartphone use^43–45^, to the best of our knowledge, there is no research specifically addressing the impact of the age of first social media adoption on students’ learning outcomes.

To fill this evidence gap, we aim to provide a rigorous estimation of the impact of early social media use on the learning outcomes of students throughout their school career. We focus on social media use as it is used via personal smartphones, reflecting the conditions of the current socio-technical landscape. We hypothesize a negative effect through two different mechanisms. On the one hand, many studies highlight the distracting potential of social media in everyday life, which could displace part of the time students directly dedicate to homework^46^ or—especially when used through a smartphone—increase problematic multitasking^47^. Early social media use could also displace time dedicated to sleep^48^ or physical movement^49^, which indirectly have a positive impact on academic performance^50–52^. On the other hand, the content and the type of relationships enabled by spending time on social media during pre-adolescence could also negatively affect mental well-being^15^ and, consequently, indirectly impact academic achievement^53,54^. Finally, we expect that longer exposure in years will lead to increasingly negative effects, consistent with research on cumulative media impacts^55^ and the heightened vulnerability of pre-adolescence to problematic social media use^17^.

We administered an ad hoc retrospective survey to a sample of students enrolled in 10th and 11th grade during the 2023–2024 school year (6,609 upper-secondary high school students residing in Lombardy, a region located in northern Italy). The survey collected detailed data on students’ background, educational trajectory, grade in which students were first exposed to a set of technological devices, existence of parental control practices, current modalities of smartphone use, and a wide range of school and life attitudes. Our core variables are the grade at which students opened their first personal social media profile and obtained their first personal smartphone. The sample is representative by school type at the regional level. We then merged the dataset with their longitudinal learning performance data at grades 2, 5, 8 and 10 collected through standardized tests by INVALSI (the Italian National Institute for the Evaluation of the Education and Training System). Owing to school closures in 2020, only the students from the birth cohorts 2007 and 2008 (the modal cohorts for 10th and 11th graders in 2023–2024) have been surveyed 4 times by INVALSI and are hence the target of our analysis. The present study uses the merged dataset, which comprehends 5,227 high school students. In this sample, the effect of early social media profile opening has been estimated by means of a difference-in-differences (DiD) strategy.

## Results

### Descriptive analyses

**Fig. 1:**
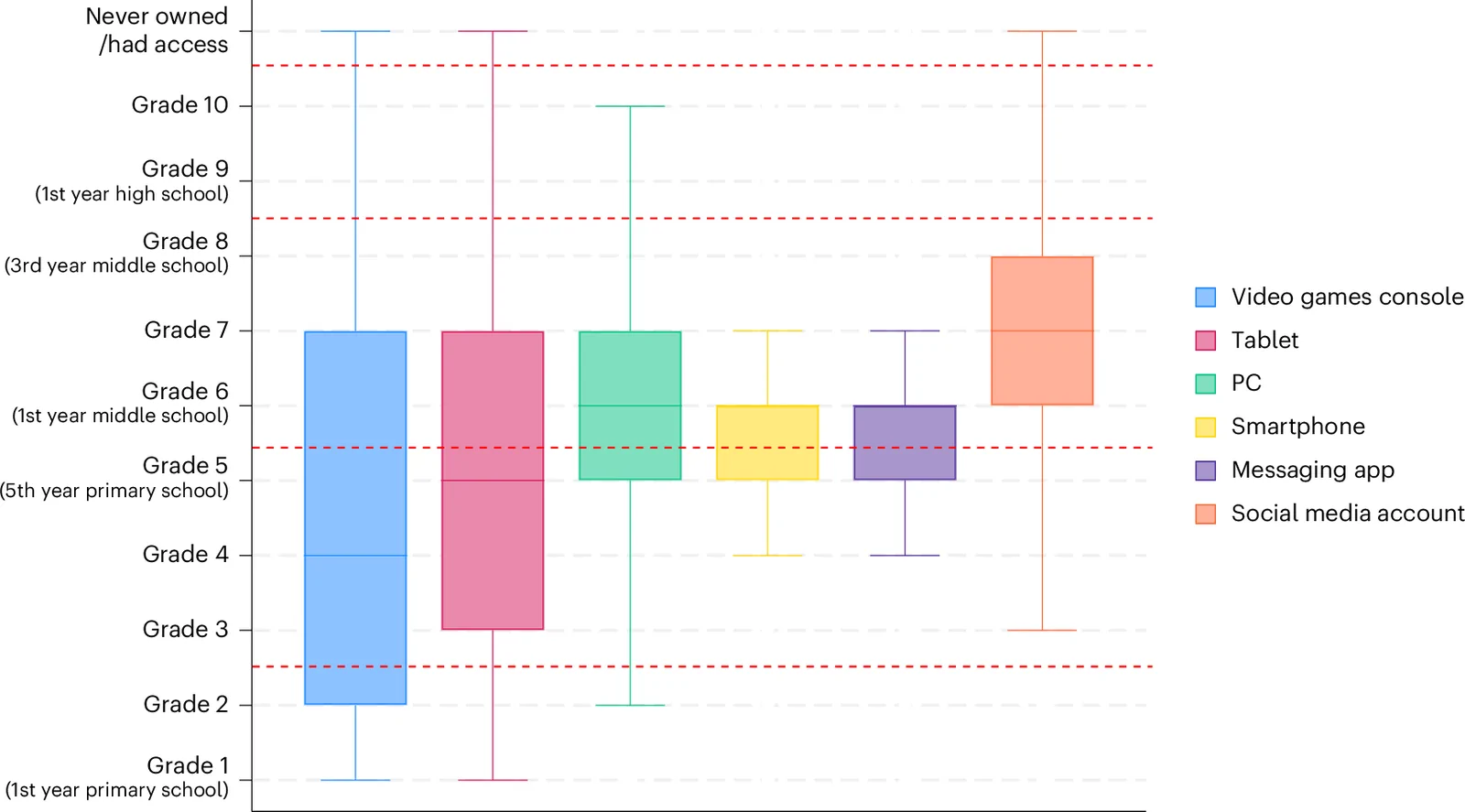
Exposure to technology during primary, lower- and upper-secondary school, box plot. Notes: *N* (students) = 5,227. The centre line indicates the median; box bounds indicate the 25th and 75th percentiles; whiskers extend to the lower and upper adjacent values, defined as the most extreme observations within 1.5 times the interquartile range from the lower and upper quartiles, respectively. The horizontal red dashed line represents the timing of the INVALSI competence tests, which take place at the end of grades 2, 5, 8 and 10. The original questions were worded differently according to each device or account type. Students were asked to indicate the grade in which they first had (i) their own internet-connected smartphone, (ii) access to video game consoles, (iii) independent access to tablet and computer, and (iv) a personal profile for messaging apps and social media accounts.

Recent cohorts of students start to approach technology from a very early age (Fig. 1). In our sample, the median grade at which students started to use a video game console is grade 4, followed by the independent use of a tablet (grade 5). Grade 6 marks the passage to lower-secondary (middle) school and to partial technological independence. This is the median grade for independent access to a computer, ownership of a personal smartphone connected to the internet. With respect to smartphones, 47% of the students received theirs as a present during grade 6. By the end of lower-secondary school (end of grade 8 in Italy), 98% of the students own a smartphone. Messaging apps are normally acquired at the same time as a smartphone or the following year, despite the fact that most messaging apps require users to be at least 13 or 14 years of age. Social media came last for this cohort of students: 11.2% of them opened a profile during primary school, 29.5% during grade 6, 25.0% during grade 7 and 18.0% during grade 8. Only 16.3% waited until grade 9, which is the minimum age set by the Italian law for autonomous access (Italian Legislative Decree 101/2018).

The robust association between the number of years of exposure to social media (measured at grade 8) and standardized competence scores outcomes at the same grade level in Italian and mathematics reveals a negative association (beta coefficient (*B*) = −0.05 s.d. in Italian competences for every additional year of exposure (95% confidence interval (CI) −0.07, −0.03); degrees of freedom (df) = 10 and −0.04 in mathematics (95% CI −0.05, −0.02); df = 10). These results hold also when we take other forms of exposure to technology into account by adding them as regressors in the model (in Italian, *B* = −0.06 (95% CI −0.08, −0.03); df = 14; −0.04 in mathematics (95% CI −0.05, −0.02), df = 14).

### Longitudinal evidence

The above-mentioned association is purely descriptive in nature. To estimate the effect of early social media exposure on students’ competences in mathematics, Italian and English language, we used a DiD strategy implemented using student fixed-effects models combined with statistical matching. Our strategy focuses on the comparison of the outcomes of four groups of students, categorized according to the grade in which they first opened a social media account: 6th grade, 7th grade, 8th grade (collectively referred to as early users) and 9th grade or later, with the latter group representing those who abide by the law and wait until the end of lower-secondary school (in the Italian school system, students transition to upper-secondary school—a tracked path—after grade 8). Since we cannot estimate the effects for students who opened an account during primary school owing to lack of enough prior data points, we excluded them in the longitudinal analyses (11% of the sample).

In our main analyses, we compare each early user group separately with the late user group, after having matched them on a wide set of observable characteristics. This choice is motivated by two main considerations. First, regarding early users, both international literature and our data suggest that parents typically give their children a smartphone during lower-secondary school, a time that often coincides with the creation of their first personal social media account. Second, in the case of late users, 9th grade aligns with the age of 14—the legal minimum set by Italian law in accordance with the European General Data Protection Regulation. This same age threshold is also endorsed by various professional bodies and clinical experts as appropriate for owning a smartphone and/or opening a personal social media account, both internationally^2^ and in Italy^56,57^. Because social media use largely overlaps with smartphone ownership in this age group, we estimate their combined exposure (rather than disentangling the two) and exclude the few students reporting account opening without a personal smartphone.

Table 1 shows the composition of the four groups on the basis of the variables used in our matching strategy. The variables were chosen on the basis of the known predictors of both the dependent and independent variable to avoid confounding. Sex, parental education, characteristics of the household, nursery attendance, economic hardship and parental control practices are known predictors of early exposure to social media^29,43,58^ and of achievement inequalities^59–63^. We added indicators of previous exposure to technology to account for parenting educational style in relation to media and technology (an otherwise important, unobserved factor) and of previous school achievement, measured both in terms of marks and test scores. In line with this literature, we can observe that the four groups are characterized by a strong social gradient: students who opened a profile earlier come from families that are, on average, less educated and less often are intact two-parent families. These students started their digital transition earlier in virtually all devices, and they exhibited lower scores in competence tests and in teachers’ marks. The groups are, instead, more balanced in terms of sex, migratory background, perception of hardship and nursery attendance.

**Table 1:** Average student characteristics on the basis of the grade a social media profile was opened (average or percentage)

	Social media in 6th grade	7th	8th	9th or later
Socio-demographics				
No parent with a high school diploma (%)	19 (0.001)	16 (0.182)	14 (0.602)	13
At least one parent with a high school diploma (%)	49 (0.000)	51 (0.000)	46 (0.005)	40
One parent with a tertiary degree (%)	22 (0.009)	20 (0.000)	26 (0.888)	25
Two parents with a tertiary degree (%)	11 (0.001)	13 (0.000)	14 (0.003)	22
Female (%)	54 (0.466)	55 (0.138)	55 (0.063)	52
Native (%)	87 (0.059)	89 (0.664)	92 (0.391)	90
Attended nursery (%)	47 (0.360)	50 (0.909)	49 (0.829)	50
Intact two-parent family (%)	80 (0.000)	82 (0.000)	85 (0.072)	88
Average subjective economic hardship index (1 high, 4 low)	3.6 (0.781)	3.6 (0.591)	3.6 (0.853)	3.6
Exposure to technology in primary school and parental control (%)				
Computer	28 (0.000)	23 (0.077)	20 (0.952)	20
Tablet	64 (0.000)	57 (0.000)	48 (0.273)	45
Video games	74 (0.000)	66 (0.000)	61 (0.000)	50
Smartphone	44 (0.000)	24	14 (0.554)	13
Ever had parental control^a^	39 (0.000)	49 (0.000)	54 (0.059)	59
School career before middle school (average standardized scores)				
Mathematics competence score, grade 5	0.09 (0.089)	0.10 (0.040)	0.13 (0.107)	0.22
Italian competence score, grade 5	0.18 (0.055)	0.22 (0.037)	0.23 (0.027)	0.34
English competence score, grade 5	0.19 (0.782)	0.20 (0.830)	0.16 (0.350)	0.21
Mark in Italian language, grade 5	−0.03 (0.017)	−0.01 (0.002)	0.03 (0.024)	0.17
Mark in mathematics, grade 5	−0.04 (0.036)	−0.02 (0.004)	0.07 (0.315)	0.13
*N* of students (max)	1,484	1,284	931	846

The analysis includes only students who opened a social media account after owning a smartphone. *P* values, shown in parentheses, are obtained by comparing each of the three early-adopter groups separately with the late-adopter group, using linear probability models in which the dependent variable is the characteristic and the independent variable is the group. Standard errors are clustered at the school level. All tests are two-sided. Model degrees of freedom are 1 for all models. ^a^Variable used for matching only in robustness checks.

Table 1 confirms that a simple comparison of outcomes across groups is likely to be biased, as parental education, family structure and early adoption of digital devices are associated with both social media use and—as is well known in the literature—competence scores. Moreover, these groups already differ in academic outcomes as early as grade 5. This is why we balanced the groups on observable characteristics through statistical matching. We then tracked outcome dynamics over time using a DiD estimator. The availability of grade 2 scores further enhances the credibility of this approach by providing an element for a pre-treatment trend comparison. The combined use of statistical matching and DiD analysis is considered a standard procedure in causal inference with longitudinal data^64^.

**Fig. 2:**
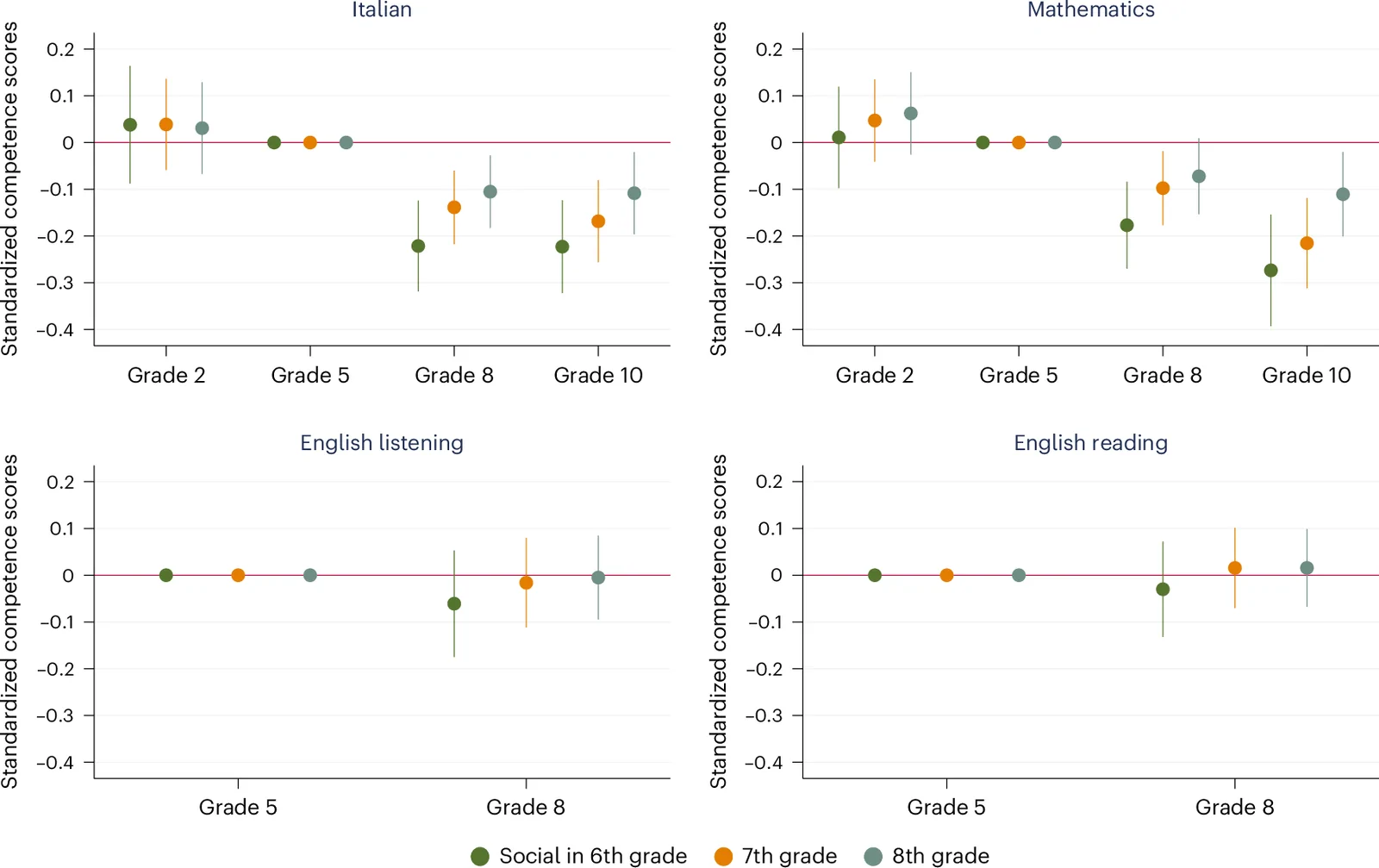
Effect of early use of social media on competences in grades 8 (Italian, mathematics and English) and 10 (Italian and mathematics). Note: results are presented as regression coefficient estimates, with error bars indicating 95% confidence intervals from two-sided tests. The coefficients display the interaction parameters between treatment status and grade from student fixed-effects models. Matching method: entropy balancing. Standard errors clustered at the individual level. *N* refers to student–time unit observations in all models. Models comparing the 6th grade early users group to the late users group: 7,601 obs. in mathematics, 7,596 in Italian, 3,842 in English listening and reading models. B in g8, math. −0.18 s.d. (95% CI −0.27, −0.08); g10 math. −0.27 s.d. (95% CI −0.39, −0.15); g8 Italian −0.22 s.d. (95% CI −0.32, −0.12); g10 Italian −0.22 s.d. (95% CI −0.32, −0.12); g8 Eng. reading −0.03 s.d. (95% CI −0.13, 0.07); g8 Eng. list. −0.06 s.d. (95% CI −0.17, 0.05). Models comparing 7th grade early users group to the late users group: 6,966 obs. in math., 6,984 in Italian, 3,546 in Eng. listening, 3,544 in Eng. reading models. B in g8 math. −0.1 s.d. (95% CI −0.18, −0.02), g10 math. −0.22 s.d. (95% CI −0.31, −0.11), g8 Italian −0.14 s.d. (95% CI −0.22, −0.06), g10 Italian −0.17 s.d. (95% CI −0.26, −0.08), g8 Eng. reading 0.02 s.d. (95% CI −0.07, 0.1), g8 Eng. list. −0.02 s.d. (95% CI −0.11, 0.08). Models comparing the 8th grade early users group to the late users group: 5,890 obs. in math., 5,864 in Italian, 2,982 in Eng. reading and listening models. B in g8 math. −0.07 s.d. (95% CI −0.15, 0.01), g10 math. −0.11 s.d. (95% CI −0.2, −0.02), g8 Italian −0.11 s.d. (95% CI −0.18, −0.03), g10 Italian −0.11 s.d. (95% CI −0.2, −0.02), g8 Eng. reading −0.0 s.d. (95% CI −0.09, 0.08), g8 Eng. listening 0.02 s.d. (95% CI −0.07, 0.09). Model df for all Italian and math. = 7; df for Eng. = 3. Obs., observations; math., mathematics; Eng., English.

The effects of early social media use on academic competences are presented in Fig. 2. In each graph, the horizontal axis represents the grade in which INVALSI assessments were administered, while the vertical axis shows standardized competence scores. The dots indicate the estimated effects for students who opened a social media account in a given grade—green for 6th grade, orange for 7th and blue for 8th—compared with the control group, consisting of late adopters (those who opened an account in grade 9 or later).

By design, all groups were statistically equivalent in grade 5 performance, as the matching procedure ensured balance in pre-treatment academic achievement, measured immediately before the earliest onset of social media exposure.

Four main findings emerge from these results. First, they provide evidence consistent with a causal negative effect of early social media use on competence scores in both Italian and mathematics across nearly all exposure groups and time points. Compared with late adopters, students who created a social media account in grade 6 scored approximately 0.22 standard deviations lower in Italian ((95% CI −0.32, −0.12); df = 7) and 0.18 ((95% CI −0.27, −0.08); df = 7) in mathematics by grade 8. By grade 10, the effect remains −0.22 ((95% CI −0.32, −0.12); df = 7) in Italian and grows to −0.27 in mathematics ((95% CI −0.39, −0.15); df = 7). For those who opened an account in grade 7, the effect was smaller but still meaningful: −0.14 ((Italian, 95% CI −0.22, −0.06); df = 7) and −0.10 ((mathematics, 95% CI −0.18, −0.02); df = 7) in grade 8, and −0.17 ((95% CI −0.26, −0.08); df = 7) and −0.22 ((95% CI −0.31, −0.11); df = 7), respectively, by grade 10. Among those who opened a social media profile in grade 8, we observed a −0.11 effect ((95% CI −0.18, −0.03); df = 7) in Italian and a non-significant −0.07 ((95% CI −0.15, 0.01); df = 7) effect in mathematics by grade 8, and −0.11 in both subjects by grade 10 ((both 95% CI −0.22, −0.02); df = 7).

This gap in performance is noteworthy. According to recent literature, effects exceeding the 0.2 standard deviations threshold in test scores can be considered as large in education^65,66^. Although there is no official equivalence between the INVALSI scores and years of schooling, some educational institutions^67^ adopt the parameter of 0.4 s.d. as the equivalent of 1 year of schooling. In this setting, a 0.2 estimate in our data is equivalent to 6 months of schooling. To give another term of comparison, in our sample, the average differences in performance in grade 5 (that is, before the opening of a social media profile) between students with 2 parents holding a tertiary degree and students with no parents who have a secondary school diploma are 0.73 in Italian language and 0.71 in mathematics. This means that the loss of competences induced by the modal timing of opening of a social media account (that is, grade 6) compared with the suggested late adoption accounts for about 30% of one of the biggest social divides in education. From another perspective, this loss in competence is, in absolute terms, roughly half the size of the average impact of intensive—and costly—tutoring interventions, which are currently among the most effective educational strategies for improving student achievement^68^.

Second, concerning the effect of cumulative exposure, the negative effect is more pronounced at each grade test the earlier the account was created. Evidence of cumulative exposure can be found in Supplementary Information 1, in which we compare seventh- and sixth-grade early adopters (Supplementary Fig. A1), and eighth- and seventh-grade, eighth- and sixth-grade early adopters (Supplementary Fig. A2). Moreover, the adverse impact not only persists in grade 10, but intensifies—particularly in mathematics and among those exposed before grade 8—despite the fact that most late adopters had become social media users by grade 10. This result appears to challenge the belief that delaying pre-adolescents’ access to social media may later result in significant negative effects on learning once they eventually gain access.

Third, the absence of statistically significant differences in grade 2 performance across groups reinforces the credibility of our causal interpretation, especially for the comparisons between late users and grade 6 and 7 early users. In these comparisons, this balance is consistent with the parallel trends assumption that underpins our DiD strategy, that is, that the academic trajectories of the matched groups would have remained aligned in the absence of social media exposure. As concerns the comparison between 8th grade and late adopters, instead, the existence of a pre-trend—albeit non-significant—imposes caution in the interpretation. We further discuss the robustness of the findings in a dedicated section.

Fourth, we find no evidence of any impact of early social media use on English competence. A plausible interpretation of this divergent result is that much of the content consumed online—especially in social media contexts—is in English, which may provide incidental exposure and practice, given the language’s global status and dominance in digital environments. This finding holds across multiple specifications and sample selections (Supplementary Figs. A1, A2 and A10–A12 in Supplementary Information 1).

Our core findings hold consistently across various robustness checks. These include variations of the matching algorithm and model specification, restricting the analysis to the 2007 cohort—which includes grade repeaters—conducting an equivalence analysis of our groups using a proxy for parental involvement (Supplementary Figs. A10–A13 in Supplementary Information 1).

Furthermore, we complement the standard DiD estimates with a sensitivity analysis based on the robust inference framework recently developed by Rambachan and Roth^69^. This analysis reinforces the idea of a significant cumulative exposure effect, with the strongest evidence for negative effects among students who opened social media accounts in 6th grade. For this group, the effect on mathematics competences in grade 10 is robust: the 95% confidence set excluded zero even when allowing post-treatment violations of parallel trends up to twice the magnitude of estimated pre-treatment deviations (Supplementary Fig. A3 in Supplementary Information 1, breakdown value $\bar{M}$ = 2). By contrast, the estimated effect of opening a social media account in 8th grade is not robust even to mild violations of the parallel trends assumption, especially in mathematics (Supplementary Fig. A5 in Supplementary Information 1, breakdown values below $\bar{M}$ = 0.5 for most outcomes). These results support the conclusions that early adoption—up to 7th grade—has a negative effect on competences in mathematics and language, while at the same time impose a cautionary note on the interpretation of the effects calculated on students who opened a social media in grade 8. In general, the effect on competences, both Italian and mathematics, shows greater robustness (that is, a greater breakdown value) in grade 10 than in grade 8 (Supplementary Figs. A3–A5 in Supplementary Information 1). Lastly, we test the internal consistency of our estimates across different matched-group comparisons. All these analyses are detailed in the robustness checks paragraph in Methods and in Supplementary Information 3.

### Additional evidence

This section reports on the estimated effects of early social media use on additional school-related outcomes, namely school marks in Italian and mathematics (as collected by INVALSI in grades 2, 5 and 8), an excellent grade 8 overall mark (8/10 or above) and academic self-efficacy (from the EYES UP survey), grade repetition in upper-secondary school and upper-secondary track at the moment of the interview (from both INVALSI and the EYES UP survey).

The analyses of school marks collected by INVALSI were conducted using the same longitudinal models described in the methodological section (Supplementary Fig. A6 in Supplementary Information 1). For the remaining outcomes, the adoption of a longitudinal design was not possible, either because the variables were collected through our retrospective questionnaire or because of their nature (for example, grade repetition or school track). For these outcomes, we adopted a matching design, using the matched groups and corresponding weights from the main analysis (Supplementary Fig. A7 in Supplementary Information 1). The results show that the early opening of a social media account is also significantly associated with other academic dimensions. The probability of repeating a grade is higher for students who open a social media profile in 7th or 6th grade compared with late users. Furthermore, the three groups of early users are significantly less likely to achieve an excellent mark at the end of lower-secondary school (that is, at the end of grade 8 in Italy) and report lower levels of academic self-efficacy. Instead, the choice of upper-secondary school track appears largely unaffected by early social media use.

### Mediation: an exploratory analysis

After establishing the negative effect of early social media access on academic competences, we now turn to examine the potential mediators of this effect. Previous research suggests that the adverse effects of social media on learning may be mediated by factors such as impaired working memory, exposure to distracting or inappropriate content, depressive symptoms and the effectiveness of parental control^70–72^.

We did not collect direct screen time metrics, as self-reported data are widely recognized as unreliable for this purpose^73,74^, nor did we gather information on the specific content consumed online. We relied, instead, on a validated scale of smartphone pervasiveness (Supplementary Information 2), which captures the degree to which mobile technology intrudes upon key areas of daily life (for example, usage before sleep, upon waking, when doing homework or at school)—domains closely linked to attention disruption and cognitive overload^75–77^. Regarding parental supervision, students were asked whether they had been subject to parental control measures, as well as whether their parents had access to their online activity. This measure is ambiguous, though, as parents might have introduced parental control after the observation of undesired behaviour on their children’s part. We might, then, overestimate the role of this variable. Finally, we included a measure of subjective well-being, as described in the ‘Data’ section.

To assess the potential role of these variables as mediators, we examined whether they are associated with both the outcome (grade 10 competence scores) and the length of exposure (years since opening a personal social media profile). We ran a series of regression analyses using the full sample, regressing competence scores and years of exposure to social media on each potential mediator (smartphone pervasiveness, life satisfaction, parental control and parental access to online activities) in separate models. In each model, the relationship is controlled for socio-demographics (sex, parental education, migratory background, family structure, pre-kindergarten attendance, perceived economic hardship and birth cohort) and grade 2 competences and marks (Supplementary Figs. A8 and A9 in Supplementary Information 1). The findings indicate that higher values on the pervasiveness index are associated with lower scores in both Italian and mathematics by grade 10. Specifically, a one standard deviation increase in the pervasiveness index corresponds to a decline of 0.11 standard deviations for Italian and −0.14 for math. Moreover, students with higher values on this index are also more likely to have opened a social media account earlier. Parental control and parental access to online activities appear to be associated with higher competence scores. Notably, students who opened a social media profile earlier were less likely to have been subject to parental supervision. By contrast, subjective well-being shows no significant association with test scores, and only a weak negative correlation with years of social media exposure. We therefore excluded it as a potential mediator.

Table 2 presents the results of the analysis. Since we lack longitudinal data on the mediators themselves, we first replicate the main effect of early social media exposure using a matching estimator with the same groups and weights as in the longitudinal analysis (row 2), to compare it with the DiD estimate (row 1). In line 3, we include school track as a control variable, which could not be used in the initial matching design but may act as a confounder. In lines 4–6, each potential mediator is introduced separately into the model with school track as a control. Each line shows the estimated effect of early social media exposure and the percentage reduction in effect size attributable to the mediator.

**Table 2:** Impact of early acquisition of social media on students’ competences and potential mediators

	Italian			Mathematics		
	6th versus 9th grade	7th versus 9th grade	8th versus 9th grade	6th versus 9th grade	7th versus 9th grade	8th versus 9th grade
(1) DiD estimate	−0.22 (0.000)	−0.17 (0.000)	−0.11 (0.016)	−0.27 (0.000)	−0.22 (0.000)	−0.11 (0.016)
(2) Matching estimate	−0.22 (0.003)	−0.17 (0.001)	−0.10 (0.055)	−0.26 (0.000)	−0.19 (0.005)	−0.10 (0.043)
(3) Matching estimate controlled for school track	−0.16 (0.003)	−0.14 (0.001)	−0.09 (0.060)	−0.21 (0.000)	−0.17 (0.006)	−0.08 (0.108)
Mediation						
(4) Pervasiveness index	−0.11	−0.10	−0.08	−0.14	−0.11	−0.04
Reduction in the parameter (%)	34 (0.047)	32 (0.21)	15 (0.107)	33 (0.007)	33 (0.044)	47 (0.371)
(5) Parental control	−0.16	−0.14	−0.08	−0.22	−0.17	−0.07
Reduction in the parameter (%)	0 (0.005)	3 (0.001)	5 (0.071)	−2 (0.000)	1 (0.005)	2 (0.115)
(6) Parental access to online activities	−0.14	−0.13	−0.09	−0.19	−0.16	−0.08
Reduction in the parameter (%)	14 (0.009)	7 (0.001)	2 (0.068)	10 (0.000)	5 (0.006)	2 (0.115)
*N* (rows 2/6)	1,852	1,709	1,436	1,848	1,697	1,448

Estimates shown in line 1 refer to the longitudinal estimates shown in Fig. 2. Models shown in lines 2–6: errors clustered at the school level. Matching method: entropy balancing. *P* value in parentheses (two-tailed tests). Line(1) g8 Italian ((95% CI −0.32, −0.12); df = 7) Math. ((95% CI −0.39, −0.15); df = 7). g7 Italian ((95% CI −0.26, −0.08); df = 7) Math. ((95% CI −0.31, −0.11); df = 7). g6 Italian ((95% CI −0.2, −0.02); df = 7) Math. ((95% CI −0.2, −0.02); df = 7). Line(2) g8 Italian ((95% CI −0.21, 0.002); df = 1) Math. ((95% CI −0.20, −0.004); df = 1). g7 Italian ((95% CI −0.27, −0.07); df = 1) Math. ((95% CI −0.32, −0.06); df = 1). g6 Italian ((95% CI −0.36, −0.08); df = 1) Math. ((95% CI −0.39, −0.14); df = 1). Line(3) g8 Italian ((95% CI −0.18, 0.004); df = 3) Math. ((95% CI −0.17, 0.02); df = 3). g7 Italian ((95% CI −0.22, −0.06); df = 3) Math. ((95% CI −0.29, −0.05); df = 3). g6 Italian ((95% CI −0.27, −0.06); df = 3) Math. ((95% CI −0.32, −0.11); df = 3). Line(4) g8 Italian ((95% CI −0.17, 0.017); df = 4) Math. ((95% CI −0.13, 0.05); df = 4). g7 Italian ((95% CI −0.18, −0.02); df = 4) Math. ((95% CI −0.22, −0.003); df = 4). g6 Italian ((95% CI −0.2, −0.002); df = 4) Math. ((95% CI −0.24, −0.04); df = 4). Line(5) g8 Italian ((95% CI −0.18, 0.008); df = 4) Math. ((95% CI −0.17, 0.02); df = 4). g7 Italian ((95% CI −0.21, −0.06); df = 4) Math. ((95% CI −0.28, −0.05); df = 4). g6 Italian ((95% CI −0.27, −0.05); df = 4) Math. ((95% CI −0.33, −0.11); df = 4). Line(6) g8 Italian ((95% CI −0.18, 0.01); df = 4) Math. ((95% CI −0.17, 0.02); df = 4). g7 Italian ((95% CI −0.20, −0.06); df = 4) Math. ((95% CI −0.28, −0.05); df = 4). g6 Italian ((95% CI −0.24, −0.036); df = 4) Math. ((95% CI −0.29, −0.09); df = 4).

The results show that DiD and matching estimates are highly consistent, and that controlling for school track alone accounts for a portion of the effect. When introduced individually, parental controls only explain a small fraction of the relationship. By contrast, smartphone pervasiveness explains a meaningful portion of the effect—between 33% and 47% in mathematics, and between 15% and 34% in Italian.

These results provide supporting, albeit purely correlational, evidence that mechanisms such as attentional disruption and excessive engagement with social media are plausible channels through which early exposure impairs academic learning.

## Discussion

This study provides robust empirical evidence regarding the impact of early social media use on students’ learning outcomes over the course of their school careers. The analysis makes use of a dataset that merges retrospective survey data with standardized academic performance data in four points in time during students’ education.

Our findings suggest a significant negative impact of early social media use on academic performance during lower-secondary school years (grades 6–8 in Italy) in mathematics and Italian with respect to a later adoption. These findings are particularly clear and robust when the students that open a social media account in 6th or 7th grade are compared with those who wait until at least 9th grade. This endorses and extends previous research^34,39,44^, which has highlighted the potential for digital distractions to interfere with students’ learning and school performance. Students who created a personal social media account in 6th or 7th grade consistently exhibit poorer outcomes across nearly every school-related dimension available in our dataset. The negative effects appear to intensify the higher the number of years of exposure, manifesting in lower academic achievement at the end of lower-secondary school, a higher risk of grade repetition, slightly less favourable upper-secondary school type and reduced academic self-efficacy.

One of the most relevant contributions of this study is the longitudinal design, which allows us to track students’ academic performance from primary to secondary school. By using a DiD approach together with a robustness check using the honest DiD framework, we have been able to disentangle the effects of social media use from other confounding variables, offering more solid causal evidence than previous correlational studies. Furthermore, the selection of students we were able to observe does not include the most problematic cases (dropouts, vocational training students and students who repeated multiple years). This may suggest that the effect in the overall population is larger than we have observed (supporting this hypothesis, Supplementary Fig. A10 in Supplementary Information 1 shows that when the analysis is restricted to the 2007 cohort—which includes some repeating students—the effect becomes slightly larger).

Our analysis also reveals that early exposure to social media has no impact on English performance. As a purely hypothetical explanation, this discrepancy may be related to the ubiquitous presence of English-language content on social media platforms, potentially offering some form of informal learning that mitigates the negative effects observed in other subjects.

Given the sensitivity of the topic and the intense, polarized debate that has emerged in recent years, these findings must be interpreted with the utmost care and responsibility. It should first be noted that the existing literature on the impact of social media use on the well-being and functional development of young people, particularly when psychological variables are considered, has not produced results as clear-cut as those highlighted in our study. This discrepancy may in part be attributed to the specific outcome we have focused on—namely learning outcomes. Indeed, the literature on learning outcomes related to social media usage has been more consistent in showing negative effects compared with studies examining subjective well-being or other psychological outcomes. This may prompt future researchers to investigate whether the negative impact of early social media exposure is driven primarily by time displacement and cognitive overload rather than by the harmful effects of social media content, which would explain why it appears more clearly in learning outcomes than in mental health measures. Such a hypothesis can be supported by the mediating role of smartphone pervasiveness that we found.

Most existing research on the impact of social media use on learning has focused on the amount of use over short periods, with the latest meta-analyses showing effect sizes from near zero to small for general use^32–34^. Clearer negative impacts have emerged for problematic social media use^78,79^. As noted in the literature review, studies specifically examining the impact of early social media use are lacking, although some longitudinal studies have found negative impacts of early smartphone use on learning^43–45^. These studies, where a direct comparison is possible, found effects ranging between 0 and 0.11 standard deviations. The magnitude of the impacts we find seem higher (Table 2), suggesting that access to social media could be a more relevant factor than the mere possession of a smartphone during pre-adolescence when it comes to learning outcomes. In general, analysing the age at which social media activity starts may offer a more effective way to capture both the quantity of exposure (in years) and the developmental stage of children when they first encounter these environments. It is indeed possible that the effects of social media exposure during early adolescence differ from those occurring during adolescence^17^.

Because DiD is a non-experimental method, it struggles to control for all plausible confounding variables as effectively as randomized experiments. Attempts to match groups on key variables may still result in systematic differences that bias the findings. For instance, while we controlled for previous performances, socio-economic background and parental education, there may be other unobserved factors, such as family dynamics or individual personality traits that express themselves during early adolescence, affecting both social media use and academic outcomes. Although we checked the existence of a parallel trend before the ‘treatment’, during primary school, we cannot categorically exclude the possibility of confounding elements that we failed to control for. Therefore, we interpret the estimates as causal, conditional on the plausibility of the parallel trends assumption and the absence of time-varying confounders. In addition, our focus on a northern Italian sample limits the generalizability of the results to other cultural or educational contexts. Indeed, it is important to emphasize that these results should be interpreted in light of the social media context of the years 2019–2023 in Western Europe and the United States. During this period, platforms such as TikTok and Instagram were dominant and shared similar features. Short-form videos consumed via social media were on the rise, and social media was accessed almost entirely through personal smartphones in the age range of our sample. Therefore, it is possible that a different social, economic or technological context for the use of social media could produce different results.

Nonetheless, the findings of this study carry important implications for educators, parents and policymakers. First, they emphasize the need for more structured guidelines and digital literacy programmes in schools aimed at developing appropriate awareness of social media use among young students and parents, particularly those from disadvantaged backgrounds. Second, they suggest the need to regulate the engagement mechanisms embedded in these platforms, particularly when they are used by minors. Moreover, in the short term, policies that delay social media access until children are cognitively mature enough to manage its potential distractions could be beneficial. Some of these policies are already in place. For example, within the context of this analysis (Italy), a regulation based on the General Data Protection Regulation prohibits individuals under the age of 14 from independently accessing digital platforms. However, this regulation and similar ones are largely disregarded in Italy, as well as in other parts of Europe and other countries overseas. A cultural intervention that provides parents and educators with concrete justifications for its enforcement could help.

Future research should expand on this study by examining the role of other digital devices and considering how different types of media use—whether passive consumption or active engagement—affect learning outcomes. Finally, it will be important to investigate longitudinally whether early social media exposure impacts psychological well-being and social relationships as clearly as it seems to impact learning outcomes.

## Methods

### Data

To address our research questions, we combined two data sources: (i) survey data from the EYES UP project on digital habits during childhood and early adolescence, and (ii) INVALSI data on students’ learning outcomes.

As part of the project ‘EYES UP—Early Exposure to Screens and Unequal Performance’, we administered a web-based survey to high school students (10th and 11th grades) in five provinces in northern Italy—Brescia, Cremona, Mantua, Milan and Monza e Brianza. We contacted all these secondary school institutes (*N* = 552) via e-mail and telephone; 28 agreed to participate. The final sample of schools is representative, in terms of school type, of the distribution of school institutes in the selected provinces (see Supplementary Table A1 in Supplementary Information 1). No statistical methods were used to pre-determine our sample size, but it is larger than those reported in previous publications on the relationship between social media use and learning outcomes^32–34^. A team of trained interviewers assisted students in completing the online questionnaire during regular school hours. The survey collected retrospective information on students’ educational trajectories, the timing and modalities of their acquisition and use of digital devices, parental control and restrictions over device usage, as well as current satisfaction with various life domains and overall subjective well-being. Data collection took place between late October 2023 and early February 2024. Before data collection, we conducted a two-phase pre-test of the questionnaire aimed at assessing the comprehension, clarity and completeness of the questions and response options. In the first phase, we carried out 10 cognitive interviews focused on selected questions from the questionnaire. In the second phase, we administered the full web questionnaire in six second- and third-year classes (123 students) selected from two schools not part of the survey sample. At the end of the two phases, the research team revised the questionnaire based on the feedback received (phase 1) and the evidence that emerged (phase 2). We obtained complete responses from 6,609 out of 7,668 students enrolled in participating classes—with the shortfall primarily owing to student absences during survey administration. The research protocol and the informed consent statements have been approved by the Data Protection Officer of the University of Milan-Bicocca on 24 March 2023. Although not mandatory for this kind of research in the Italian legal context, we also obtained the approval of the ethical committee of the same university on 7 June 2024 (protocol number 0222504).

INVALSI is the Italian National Institute for the Evaluation of the Education and Training System. It collects standardized data on student achievement by administering tests in Italian and mathematics in grades 2, 5, 8, 10 and 13, and in English in grades 5 and 8. Using a longitudinal anonymous identifier provided by the Italian Ministry of Education, we merged EYES UP retrospective survey data with respondents’ INVALSI test records and with their past school marks in Italian and mathematics. Through this procedure, we created an integrated longitudinal dataset that tracks the entire academic trajectories of current 10th and 11th graders and combines it with our survey.

### Variables

#### Learning outcomes

The core learning outcomes used in this study are represented by the scores provided by INVALSI in mathematics, Italian and English competences (listening and reading comprehension). INVALSI computes these scores by applying Rasch models to each subject-specific assessment. It is important to note that the resulting scores are not directly comparable across grades or, before 2018, across cohorts within the same grade. To enhance interpretability, we standardized all scores within each grade at the national level. As a result, these standardized measures can be interpreted as deviations from the national mean, expressed in standard deviation units for each subject-specific competence.

We also draw on longitudinal data from the INVALSI databases regarding students’ school marks. INVALSI records students’ marks in Italian and mathematics during the same academic years in which standardized tests are administered—specifically, in grades 2, 5, 8 and 10. These marks refer to the marks attributed to students at the end of the first semester, so they may not accurately reflect students’ proficiency, since teachers might play strategically with the mark to ‘stimulate’ students during the second semester. Marks are collected separately for oral and written tests, but the number of missing values is very high for written tests (over 80%), while very few are missing for oral tests (under 10%). Given the very high correlation between the marks in oral and written tests (83%, 95% and 98% in grades 2, 5 and 8, respectively, during the school years used in this paper), we took the average between the two, to obtain one only measure per subject. If values were missing for only one type of mark (typically, written tests), the average was imputed with the non-missing value. However, this limitation introduces some noise in the measure. INVALSI data allow us also to identify students who had to repeat the year (an information that we checked with our questionnaire), and track enrolment. In addition, we include two additional school career outcomes derived from the EYES UP questionnaire: final lower-secondary school mark and academic self-efficacy (see later).

It should be noted that standardized learning outcomes serve as a more neutral indicator compared with measures of digital ill-being or well-being^27^. Indeed, learning outcomes have the potential to capture both positive and negative effects without the bias inherent in scales that focus exclusively on either positive or negative dimensions—scales that are not simply opposites of one another.

#### Exposure to social media

We measure technology use through a battery of items contained in the retrospective section of the questionnaire. Our primary focus is on the grade in which students first opened a social media account, provided that they already had their own smartphone connected to the internet, based on the questions: ‘What grade were you in when you got your first social media profile (for example, Facebook, Instagram, TikTok)? If you got it during the summer, indicate the grade you would have entered in the following school year’. ‘What grade were you in when you received a smartphone connected to the internet (both wi-fi and mobile data) to keep with you? If you got it during the summer, indicate the grade you would have entered in the following school year’. Self-reported age and class of first smartphone acquisition have been used in existing research, with high response rates and evidence that students can easily retrieve and situate this event in time, as it represents a relevant moment in their lives^43–45^.

We also asked students to report the grade in which they started to use their first video game console or tablet independently, as well as the grade when they began to use a computer independently. We chose to ask respondents to report their grade level rather than age for two main reasons. First, aligning technology adoption with school grade was essential to link retrospective exposure to the timing of standardized assessments, which are administered at the end of the academic year. Second, pre-testing of the questionnaire indicated that students recalled the grade in which they first began using a given technology more reliably than their exact age. By design, our retrospective measures exclude earlier periods in which students may have accessed technology (for example, social media or smartphones) through siblings, parents or other relatives. While this definition may appear overly narrow, we contend that the potential positive or negative effects highlighted in the literature—especially regarding social media use—are most likely to manifest fully only when students have access to a personal device or account^80^. Age at first smartphone and social media account, in particular, are pivotal moments in the lives of children and adolescents, leaving a lasting imprint on their memory. Drawing on our previous research^44^, we have found that these events are consistently and vividly recalled. Research on memory in self-reported measures confirms that first experiences, turning points and events that mark the beginning of new social practices are easily remembered^81^. The arrival of the first smartphone and the opening of the first social media account can be considered landmark events^81,82^. Therefore, we consider the use of age at first smartphone and social media use as a proxy for exposure to be a strength of this analysis compared with self-reported screen time, which is the most common measure in the literature (see above).

#### Psychological variables and usage intensity

A dedicated section of the questionnaire focused on assessing the pervasiveness of mobile devices in students’ daily lives, as well as their well-being, life satisfaction and self-efficacy. In this paper, we used the following indexes. All of them are based on or derived from validated scales whose items, construct validity and reliability are detailed in Supplementary Information 2:

- Smartphone pervasiveness^75^: students were asked how frequently they check their smartphones in real-life situations (for example, before going to bed, upon waking, while doing homework and so on).
- Academic self-efficacy^83^.
- General life satisfaction: measured using the Italian validated version of the scale, developed by Su et al.^84^ and adapted by Andolfi et al.^85^.

All indexes were standardized.

#### Other variables

The richness of the questionnaire allowed us to collect detailed information on students’ socio-demographic backgrounds, educational trajectories and family practices related to technology use.

The socio-demographic variables include sex, parental education (combining information from both parents), perceived family economic hardship (measured in accordance with the EU-SILC survey), family structure (intact two-parent families, non-intact two-parent families and single-parent families), migratory background (native, first-generation and second-generation migrants), nursery school attendance and birth cohort.

As for family technology-related practices, we considered whether the student’s smartphone was subject to parental control (and, if so, up to which age), as well as the frequency with which parents accessed the electronic school register—an online platform where teachers and school staff report absences, homework assignments, grades, notices and disciplinary records.

### Analysis

#### Sample selection

We focused on students born in 2007 and 2008—the modal cohorts within our sample—excluding those from earlier cohorts, who typically had repeated multiple school years. This decision was motivated first by the availability of data linkage with the INVALSI database (for example, INVALSI did not administer standardized assessments in 2020 owing to the extended COVID-19 school closures in Italy). The second reason is substantive: given the rapid pace of technological change, we aimed to include only students who experienced a comparable technological and environmental landscape during their developmental years. Notably, students in these cohorts also experienced widespread school closures owing to COVID-19 during their first 2 years of lower-secondary school, and were thus similarly exposed to a forced and simultaneous shift towards increased technology use during a critical developmental window.

Starting from our original sample of 6,609 students, we first excluded those enrolled on 3-year vocational courses (459 students), as they lack the longitudinal identifier required for merging with INVALSI data. We then excluded students from cohorts different from other than 2007 and 2008 (*n* = 897), as well as those with no match in the INVALSI database (*n* = 26). This resulted in a final analytic sample of 5,227 students. These students are included in our descriptive analyses. For the longitudinal analyses of the effects of social media use, we further restricted the sample to students who opened a personal social media account after grade 5 (*n* = 4,641) to exploit 2 ‘pre-treatment’ points in time (2nd and 5th grades). Because we are interested in the combination of a social media account and smartphone ownership, we excluded students who opened a social media account before they owned a smartphone (*n* = 96; final sample size = 4,545 students).

Our sample is affected by selection issues arising from school dropout and grade repetition, in addition to the previously mentioned exclusion of students on short vocational courses. While we cannot precisely quantify the number of students from the 2007–2008 cohorts who dropped out, repeated multiple grades or opted for 3-year vocational training, available data provide some indication. According to our estimates, 1.6% of students had repeated more than 1 school year (typically belonging to the 2005 cohort or earlier), and an additional 7% were enrolled on short vocational courses. School dropout rates are more difficult to estimate accurately, but recent statistics suggest that approximately 8% of students in this region of Italy drop out of school altogether^86^. These students tend to come from disadvantaged backgrounds, such as migrant families or those experiencing economic hardship^86^. Given this context—and the higher incidence of early and unsupervised screen use among students from disadvantaged households^87–89^—we argue that the selective nature of our sample likely results in a lower-bound estimate of the causal relationship between the age at which students open a social media account and their academic competences. Another source of selection—partially mitigated by the design of our survey—concerns students from the 2007 cohort who repeated a single school year. Students could repeat a year only in upper-secondary school, given that during pandemic years, the ministry blocked year repetitions in lower-secondary school. In our data, these students represent approximately 15% of the cohort, a figure consistent with regional statistics on grade repetition^90^. To assess the extent to which this source of selectivity may (upwardly) bias our estimates, we conducted separate analyses focusing exclusively on the 2007 cohort. The results of this robustness check are presented in Supplementary Fig. A10 in Supplementary Information 1. The negative effects shown in this figure are slightly stronger than those presented in the paper, as expected, since repeating students typically come from disadvantaged backgrounds, and thus are also likely to have opened a social media profile earlier.

#### Longitudinal design—identification strategy

This section outlines the identification strategy used to estimate the causal impact of the age at which students first opened a social media account on their academic and learning outcomes, given the possession of a personal smartphone. We began by categorizing students into four groups based on the grade in which they opened a personal social media account: 6th, 7th, 8th and 9th grade or later. Following previous literature, we designated the latter group—those who delayed social media use until after lower-secondary school—as the control group. The first three groups represent varying degrees of exposure during early adolescence. To leverage the longitudinal structure of the INVALSI data, we restricted the analysis to students who opened their first social media account after completing primary school. This approach enables us to use grade 2 standardized test scores as a pre-treatment measure to assess baseline academic trends. Each of the three ‘exposed’ groups was then matched separately with the control group on the basis of observable characteristics. The matching covariates included socio-demographic factors, indicators of technology use during primary school, marks at the end of the primary education cycle and—crucially—competences measured on grade 5 standardized tests, which precede the onset of social media use (see Table 1 for the full list of matching variables). To ensure robustness, we implemented three matching techniques: (i) entropy balancing, (ii) nearest neighbour matching without replacement using a caliper of 0.05, and (iii) radius matching with a caliper of 0.03 (Table 3). In this paper, we primarily report the results obtained through entropy balancing, as this method has been shown to achieve superior covariate balance at baseline^91^ and allows the full sample to be retained through the use of observation-specific weights. The following table presents the number of observations included in each matched comparison.

**Table 3:** Number of observations per group and matching method

	6th	7th	8th	9th or later
*N* (total)	1,484	1,284	931	846
Entropy balancing	1,484	1,284	943	846
Radius 6th versus 9th	1,237			710
Radius 7th versus 9th		1,084		710
Radius 8th versus 9th			797	710
Nearest neighbour 6th–9th	534			534
Nearest neighbour 7th–9th		620		620
Nearest neighbour 8th–9th			629	629

In analyses conducted on the groups matched using entropy balancing and radius algorithms, we implemented our DiD strategy by means of a student fixed-effects model, as described in equation (1):

1 $${Y}_{{it}}={\theta }_{i}+\beta {T}_{t}+\delta {D}_{i}+\eta (T\times D)+\varepsilon ,$$

where *Y*_*it*_ represents the outcome at time *t* for individual *i*, *θ* is the student fixed effect, *T* is the time fixed effects (grades 2, 5, 8 and 10), *D*_*i*_ is the social media user status, equal to 1 for students belonging to the early users group and 0 otherwise, and finally *η* represents the set of parameters of interest (interactions between *T* and *D*) at time 2 (to check for the existence of pre-treatment trends), 8 and 10 (to assess effects at grades 8 and 10). Note that our research design is devoted to the detection of age-specific effects. Our empirical estimands^92^ match our theoretical research questions and hypotheses. Hence, estimators developed for dynamic DiD settings^93^ would not be appropriate in this context. In analyses conducted using the nearest neighbour algorithm, we used the matched couple fixed effect instead of the student fixed effect.

### Robustness checks

The causal interpretation of the DiD estimator relies on the assumption of parallel trends between treatment and control groups posterior to treatment. In our case, for instance, the assumption would require that no time-varying confounding factors affect the difference in average school performance between students that opened a first social media account in 6th grade (7th grade or 8th grade) and late users. Another source of potential bias concerns the exclusive reliance on a single matching algorithm.

To relax the parallel trends assumptions and avoid being tied to a single matching algorithm, we implemented a series of robustness checks, discussed in depth in Supplementary Information 3. First, we matched our groups using different matching algorithms (see Supplementary Fig. A11 in Supplementary Information 1). Second, we included, as additional matching variables, some indicators of parental behaviour recorded in the questionnaire. Technically, these variables are ‘bad controls’, since they reflect parental behaviour in grade 11. However, as we argue in Supplementary Information 3, they may proxy for unobservable parenting styles (Supplementary Figs. A12 and A13 in Supplementary Information 1). Third, we performed pre-trend plausibility tests by means of an honest DiD approach^69^, given the statistical issues related to conventional pre-trends tests^94^ (Supplementary Figs. A3–A5 in Supplementary Information 1). Finally, we assessed the internal consistency of our estimates across different matched-group comparisons (Supplementary Table A2 in Supplementary Information 1). Overall, these tests broadly support our main findings, lending further credibility to the causal interpretation of our estimates.

### Reporting summary

Further information on research design is available in the Nature Portfolio Reporting Summary linked to this article.

## Supplementary information

Supplementary Information Supplementary Information 1 (Figs. A1–A13), Supplementary Information 2 (Tables A1–A8) and Supplementary Information 3.

Reporting Summary

Peer Review File

## Data Availability

As the study relies on administrative data protected by a strict privacy policy, we release the dataset only after an additive stochastic perturbation procedure on test scores, ensuring that the data are fully anonymized. The public dataset is available via the Data Archive for Social Sciences in Italy (DASSI) at https://doi.org/10.71732/JUJ4BI.

## Code availability

The replication code of the analyses, along with the dataset, is available at the same link of the Data Archive for Social Sciences in Italy (DASSI) at https://doi.org/10.71732/JUJ4BI. Analyses have been performed using the software STATA (v15.1) and R (v4.5.0; 2025-04-11 ucrt).
