# Supplementary material for: Longitudinal effect of early social media use on standardized learning outcomes during school career
Source: Nat Hum Behav. 2026 Aug 3;10(10):1953–65. doi: 10.1038/s41562-026-02522-4 (PMC13683038; doi:10.1038/s41562-026-02522-4)
Supplement: Supplementary file 1 [file 41562_2026_2522_MOESM1_ESM.pdf]

nature human behaviour

Supplementary information https://doi.org/10.1038/s41562-026-02522-4

Longitudinal effect of early social media use on standardized learning outcomes during school career

In the format provided by the authors and unedited

Supplementary Information 1 – Additional tables and figures

Figure A1 – Effect of early use of social media on competences in grades 8 (Italian, mathematics and English) and 10 (Italian and mathematics) - 7th vs 6th graders

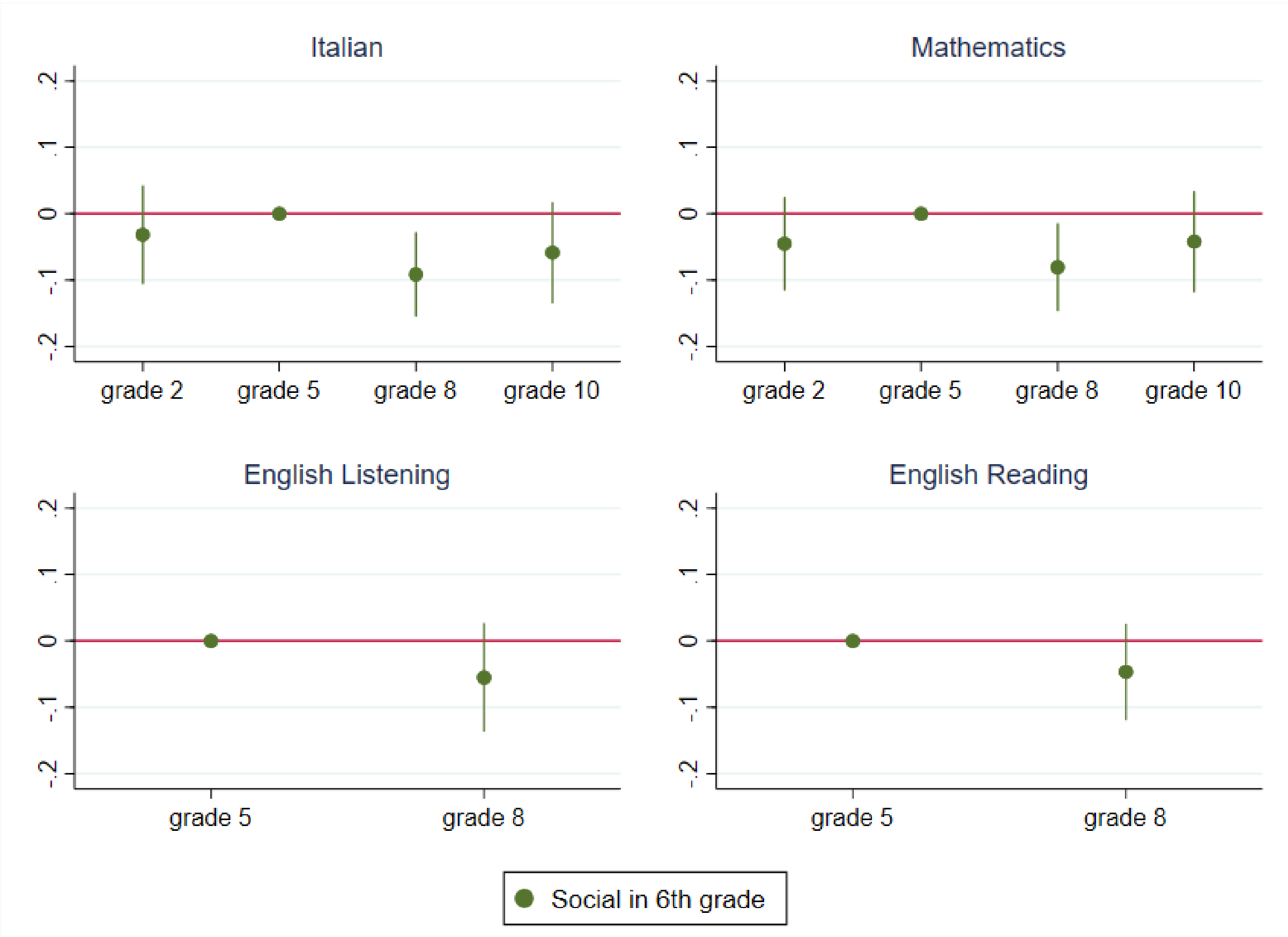

Note: results are presented as regression coefficient estimates, with error bars indicating 95% confidence intervals from two-sided tests. The coefficients display the interaction parameters between treatment status and 7th grade from student fixed-effects models. Matching method: entropy balancing. Standard errors clustered at the individual level. N refers to student–time unit observations in all models. 9,050 obs. in math. and Italian; 4,588 obs. in Eng. reading and listening. Β in g8, math. -0.08 SD [95% CI -0.15, -0.01]; g10 math. -0.04 SD [95% CI -0.12, 0.03]; g8 Italian -0.09 SD [95% CI -0.15, -0.03]; g10 Italian -0.06 SD [95% CI -0.13, 0.02]; g8 Eng. reading -0.05 SD [95% CI -0.12, 0.03]; g8 Eng list. -0.06 SD [95% CI -0.14, 0.03].

Figure A2 – Effect of early use of social media on competences in grades 8 (Italian, mathematics and English) and 10 (Italian and mathematics) - 8th vs 7th and 6th graders

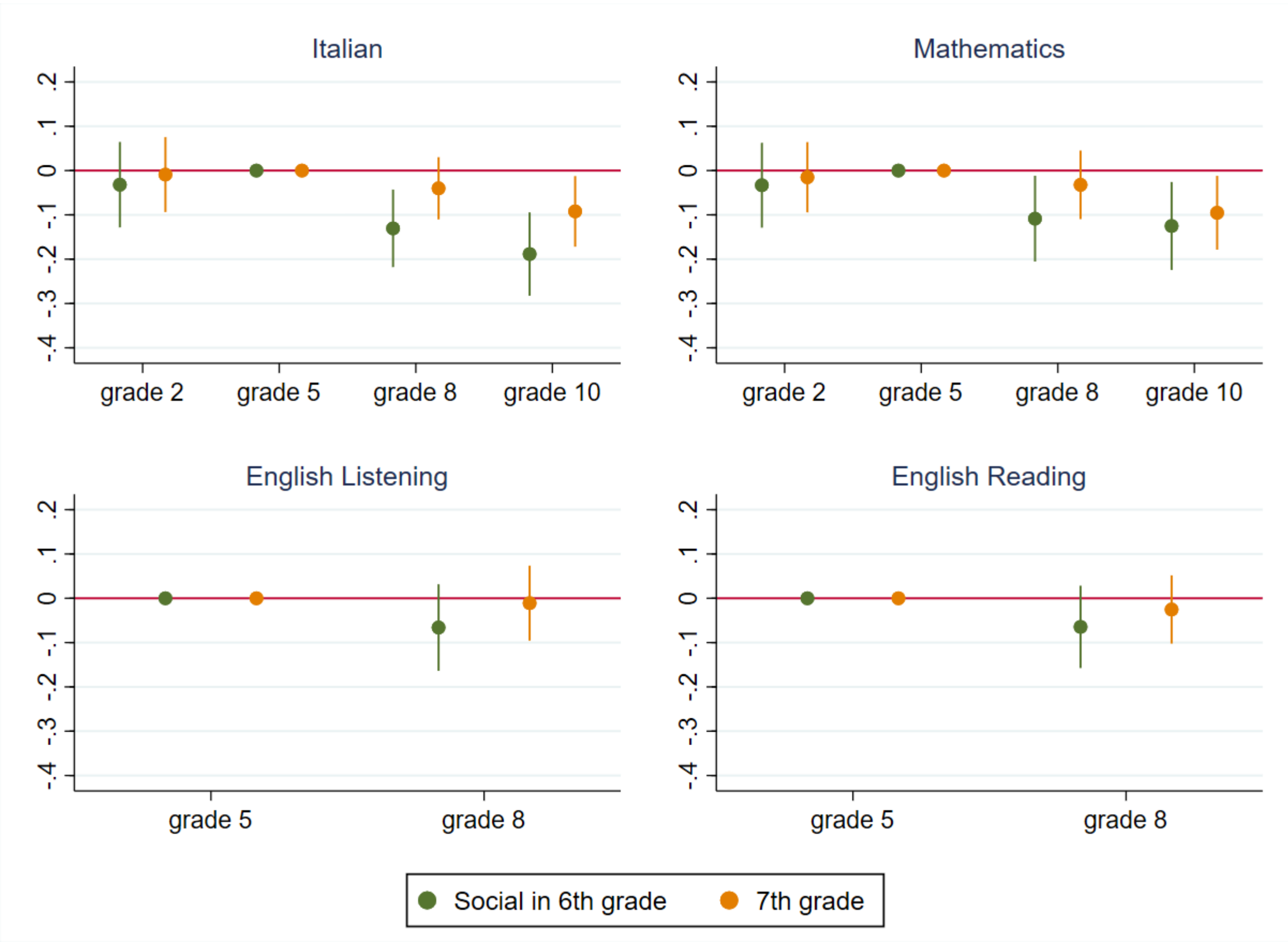

Note: results are presented as regression coefficient estimates, with error bars indicating 95% confidence intervals from two-sided tests. The coefficients display the interaction parameters between treatment status and grade from student fixed-effects models. Matching method: entropy balancing. Standard errors clustered at the individual level. N refers to student–time unit observations in all models. N in models comparing the 6th grade early users group to the 8th grade early user group: 7,947 obs. in math. and Italian; 4,024 obs. in Eng. reading and listening. N in models comparing the 7th grade early users group to the 8th grade early user group: 7,138 obs. in math. and Italian; 3,728 obs. in Eng. reading and listening. Models comparing the 6th grade early users group to the 8th grade users B in g8, math. -0.11 SD [95% CI -0.21, -0.01]; g10 math. -0.13 SD [95% CI -0.22, -0.03]; g8 Italian -0.13 SD [95% CI -0.22, -0.04]; g10 Italian -0.19 SD [95% CI -0.28, -0.09]; g8 Eng. reading -0.06 SD [95% CI 0.16, 0.20]; g8 Eng list. -0.07 SD [95% CI -0.16, 0.03]. Models comparing the 7th grade early users group to the 8th grade users: B in g8, math. -0.03 SD [95% CI -0.11, 0.05]; g10 math. -0.10 SD [95% CI -0.18, -0.01]; g8 Italian -0.09 SD [95% CI -0.17, -0.01]; g10 Italian -0.19 SD [95% CI -0.28, -0.09]; g8 Eng. reading -0.03 SD [95% CI -0.10, 0.05]; g8 Eng list. -0.01 SD [95% CI -0.10, 0.07]. Model df for all Italian and math. = 7; df for Eng. = 3.

Figure A3 – Sensitivity analysis for the effects of opening a social media profile in 6th grade on competences in grades 8 and 10 (mathematics and Italian)

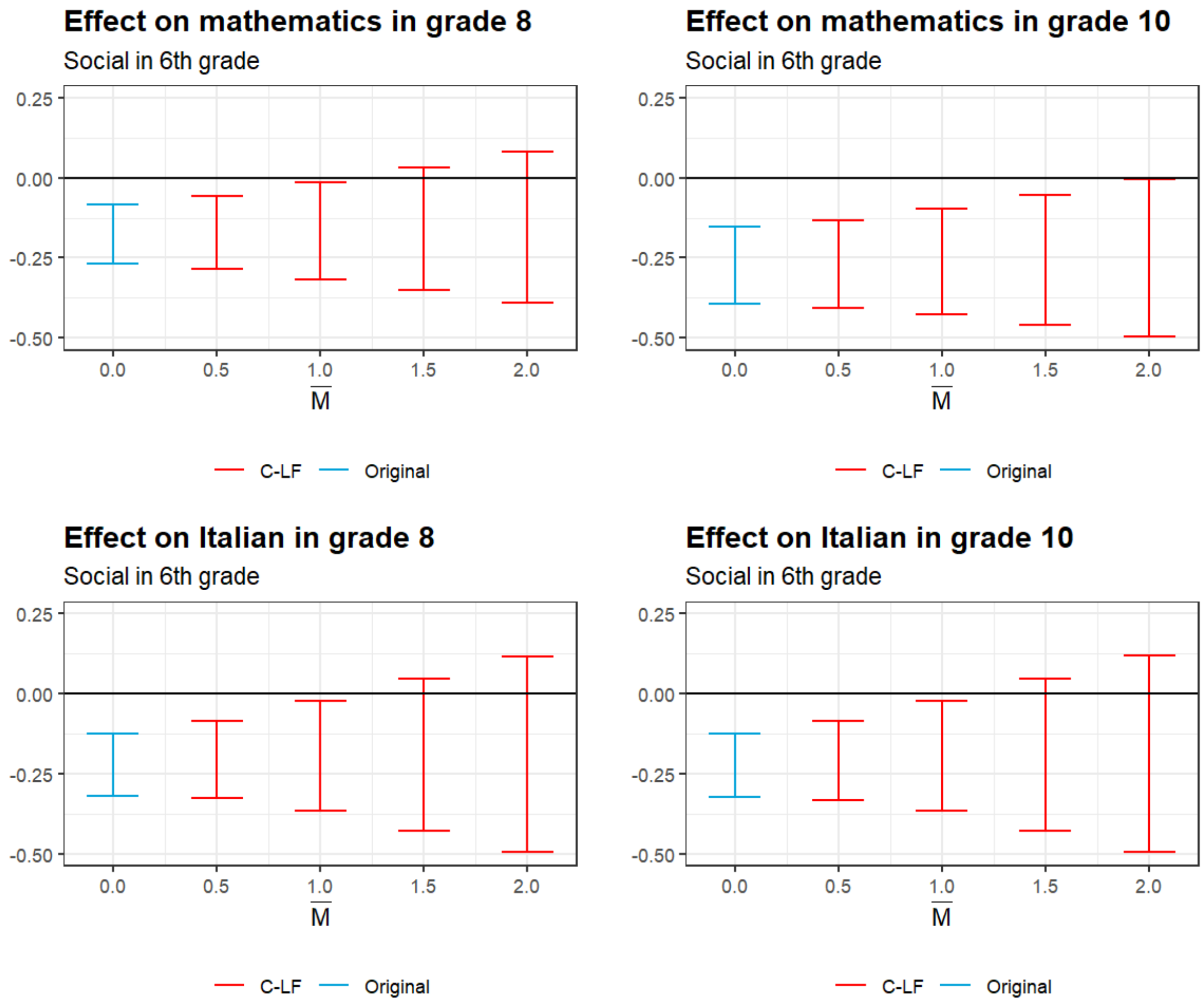

Note: Original estimates drawn from models shown in Figure 2. The blue confidence sets represent standard difference-in-differences estimates (95% CI), while the red confidence sets show robust confidence sets under the Rambachan & Roth[1] sensitivity analysis approach for varying degrees of parallel trends violations. The vertical axis displays standardized competence scores; the horizontal axis shows alternative values of $\bar{M}$, representing the maximum allowed ratio of post-treatment to pre-treatment deviations from parallel trends. Robust 95% confidence sets: Math., g8: $\bar{M}$=0.5: [-0.29, -0.06]; $\bar{M}$=1.0: [-0.32, -0.02]; $\bar{M}$=1.5: [-0.35, 0.03]; $\bar{M}$=2.0: [-0.39, 0.08]. Breakdown value: $\bar{M}$=1.5. Math., g10: $\bar{M}$=0.5: [-0.41, -0.13]; $\bar{M}$=1.0: [-0.43, -0.10]; $\bar{M}$=1.5: [-0.46, -0.05]; $\bar{M}$=2.0: [-0.50, -0.01]. Breakdown value: $\bar{M}$=2.0. Italian, g8: $\bar{M}$=0.5: [-0.33, -0.08]; $\bar{M}$=1.0: [-0.37, -0.02]; $\bar{M}$=1.5: [-0.43, 0.05]; $\bar{M}$=2.0: [-0.49, 0.12]. Breakdown value: $\bar{M}$=1.5. Italian, g10: $\bar{M}$=0.5: [-0.33, -0.08]; $\bar{M}$=1.0: [-0.37, -0.02]; $\bar{M}$=1.5: [-0.43, 0.05]; $\bar{M}$=2.0: [-0.49, 0.12]. Breakdown value: $\bar{M}$=1.5.

Figure A4 – Sensitivity analysis for the effects of opening a social media profile in 7th grade on competences in grades 8 and 10 (mathematics and Italian)

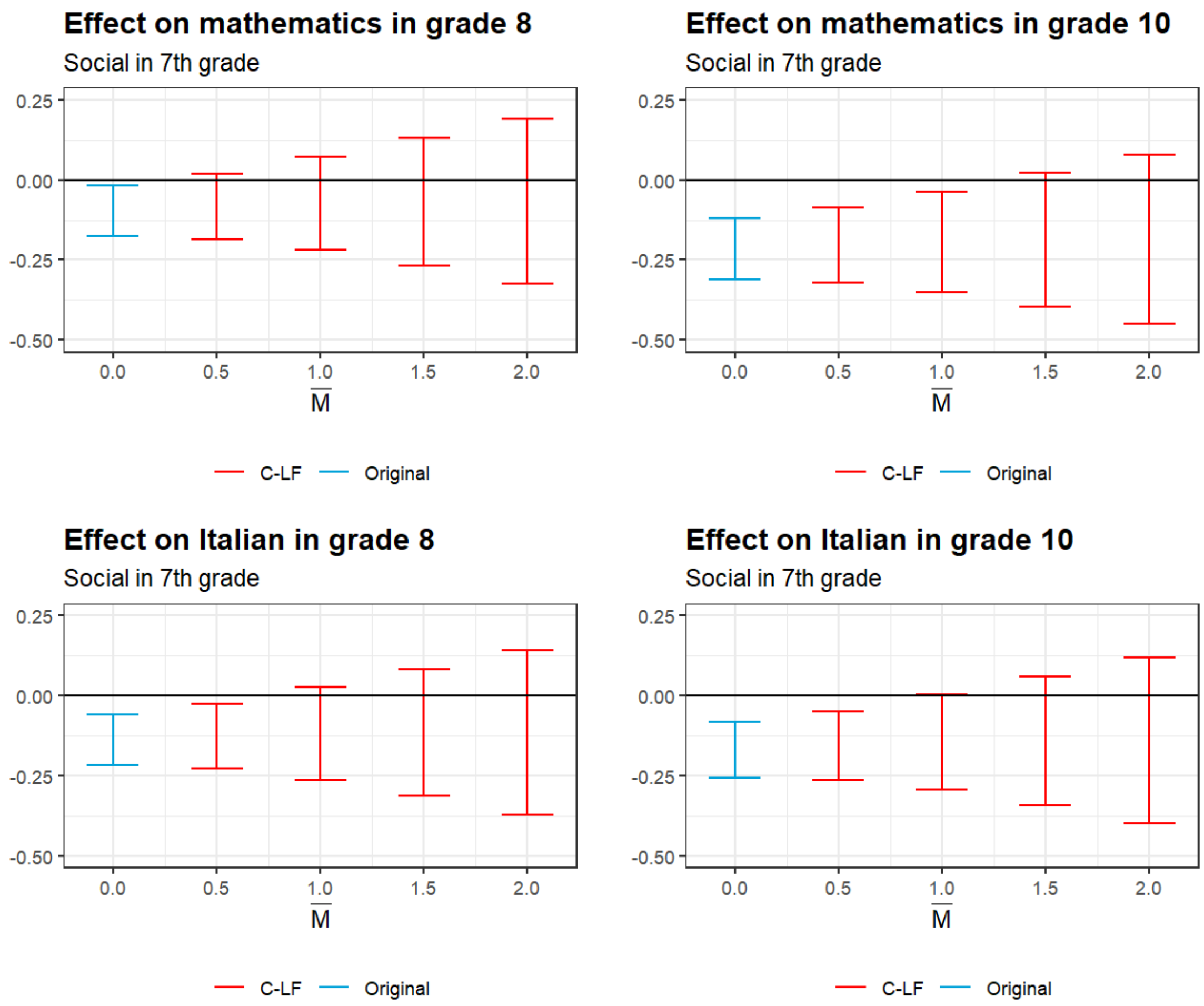

Note: Original estimates drawn from models shown in Figure 2. The blue confidence sets represent standard difference-in-differences estimates (95% CI), while the red confidence sets show robust confidence sets under the Rambachan & Roth[1] sensitivity analysis approach for varying degrees of parallel trends violations. The vertical axis displays standardized competence scores; the horizontal axis shows alternative values of $\bar{M}$, representing the maximum allowed ratio of post-treatment to pre-treatment deviations from parallel trends. Robust 95% confidence sets: Math., g8: $\bar{M}$=0.5: [-0.19, 0.02]; $\bar{M}$=1.0: [-0.22, 0.07]; $\bar{M}$=1.5: [-0.27, 0.13]; $\bar{M}$=2.0: [-0.33, 0.19]. Breakdown value: $\bar{M}$=0.5. Math., g10: $\bar{M}$=0.5: [-0.32, -0.09]; $\bar{M}$=1.0: [-0.35, -0.04]; $\bar{M}$=1.5: [-0.40, 0.02]; $\bar{M}$=2.0: [-0.45, 0.08]. Breakdown value: $\bar{M}$=1.5. Italian, g8: $\bar{M}$=0.5: [-0.23, -0.03]; $\bar{M}$=1.0: [-0.26, 0.03]; $\bar{M}$=1.5: [-0.31, 0.08]; $\bar{M}$=2.0: [-0.37, 0.14]. Breakdown value: $\bar{M}$=1.0. Italian, g10: $\bar{M}$=0.5: [-0.26, -0.05]; $\bar{M}$=1.0: [-0.29, 0.00]; $\bar{M}$=1.5: [-0.34, 0.06]; $\bar{M}$=2.0: [-0.40, 0.12]. Breakdown value: $\bar{M}$=1.0.

Figure A5 – Sensitivity analysis for the effects of opening a social media profile in 8th grade on competences in grades 8 and 10 (mathematics and Italian)

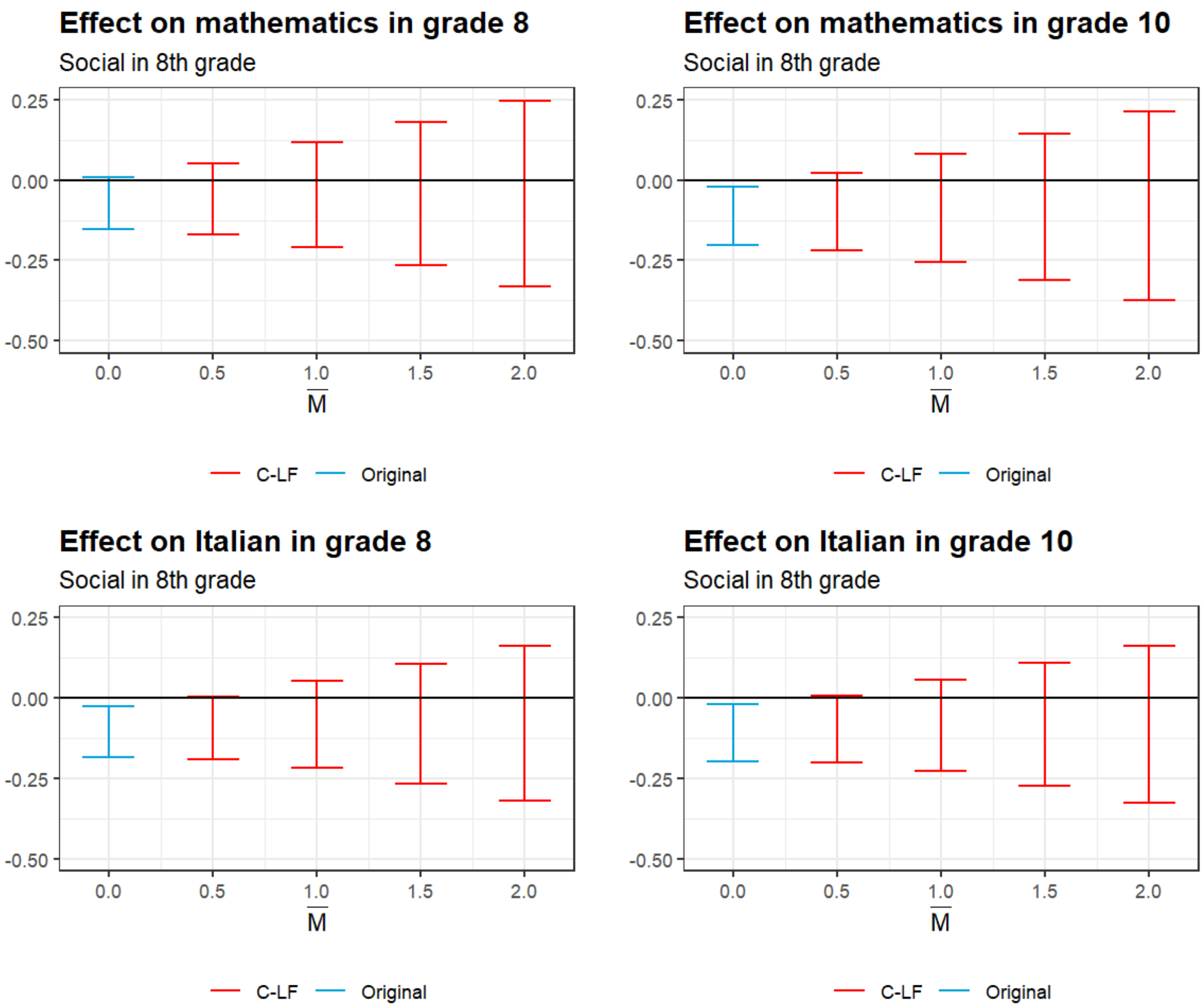

Note: Original estimates drawn from models shown in Figure 2. The blue confidence sets represent standard difference-in-differences estimates (95% CI), while the red confidence sets show robust confidence sets under the Rambachan & Roth[1] sensitivity analysis approach for varying degrees of parallel trends violations. The vertical axis displays standardized competence scores; the horizontal axis shows alternative values of $\bar{M}$, representing the maximum allowed ratio of post-treatment to pre-treatment deviations from parallel trends. Robust 95% confidence sets: Math., g8: $\bar{M}$=0.5: [-0.17, 0.05]; $\bar{M}$=1.0: [-0.21, 0.12]; $\bar{M}$=1.5: [-0.27, 0.18]; $\bar{M}$=2.0: [-0.33, 0.25]. Breakdown value: $\bar{M}$<0.5. Math., g10: $\bar{M}$=0.5: [-0.22, 0.02]; $\bar{M}$=1.0: [-0.26, 0.08]; $\bar{M}$=1.5: [-0.31, 0.14]; $\bar{M}$=2.0: [-0.38, 0.21]. Breakdown value: $\bar{M}$=0.5. Italian, g8: $\bar{M}$=0.5: [-0.19, 0.00]; $\bar{M}$=1.0: [-0.22, 0.05]; $\bar{M}$=1.5: [-0.27, 0.11]; $\bar{M}$=2.0: [-0.32, 0.16]. Breakdown value: $\bar{M}$=0.5. Italian, g10: $\bar{M}$=0.5: [-0.20, 0.01]; $\bar{M}$=1.0: [-0.23, 0.05]; $\bar{M}$=1.5: [-0.27, 0.11]; $\bar{M}$=2.0: [-0.33, 0.16]. Breakdown value: $\bar{M}$=0.5.

Figure A6 – Effect of early use of social networks on school marks in grade 8

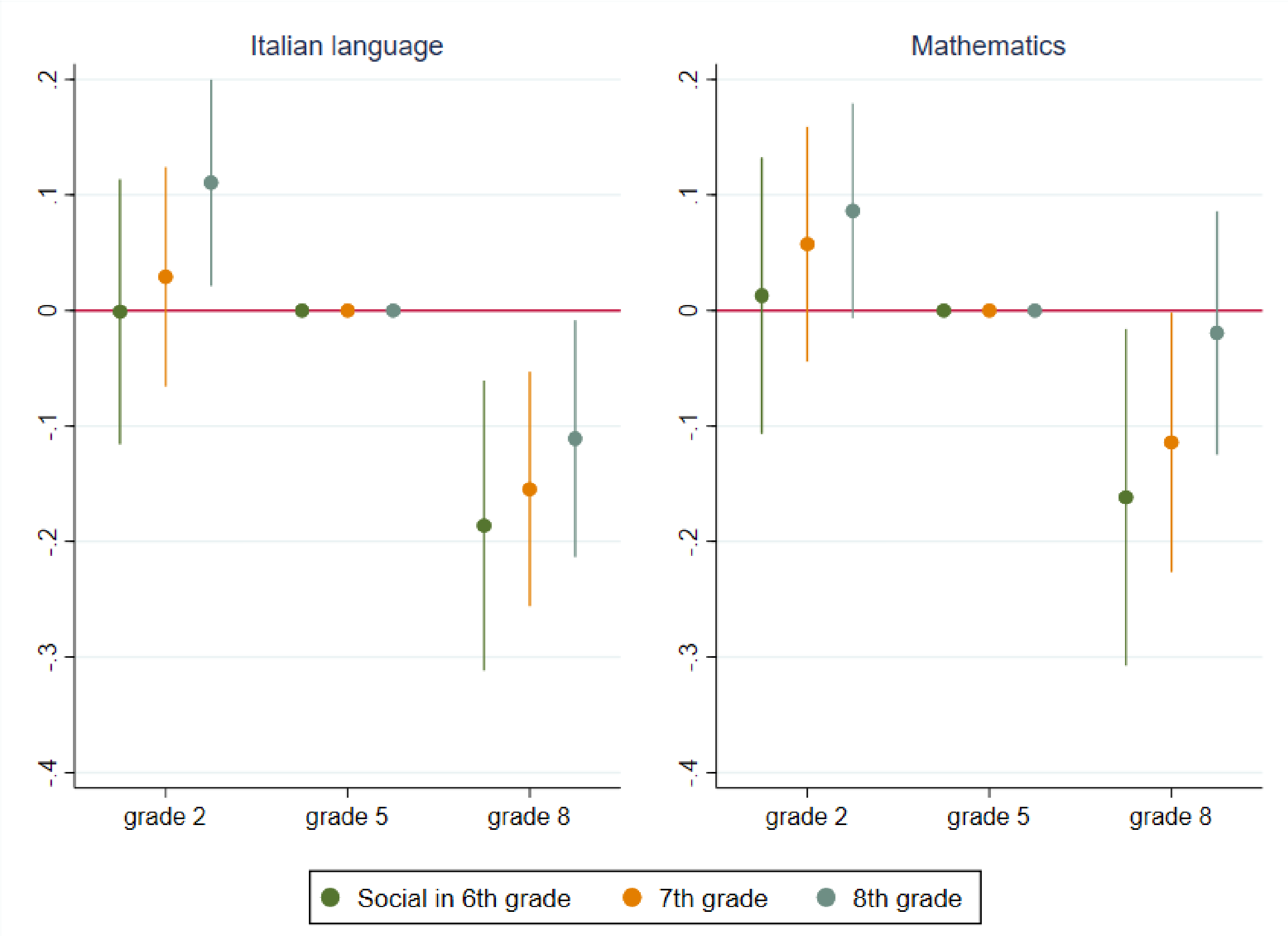

Note: results are presented as regression coefficient estimates, with error bars indicating 95% confidence intervals from two-sided tests. The coefficients display the interaction parameters between treatment status and grade from student fixed-effects models. Matching method: entropy balancing. Standard errors clustered at the individual level. N refers to student–time unit observations in all models. N in models comparing the 6th grade early users group to the late users group: 5,458 obs. in math.; 5,461 obs. in Italian language. N in models comparing the 7th grade early users group to the late users group: 5,004 obs. in math.; 5,008 obs. in Italian language. N in models comparing the 8th grade early users group to the late users group: 4,235 obs. in math.; 4,238 obs. in Italian language. Models comparing the 6th grade early users group to the late users group: B in g8, math. -0.16 SD [95% CI -0.30, -0.02]; g8 Italian -0.19 SD [95% CI -0.31, -0.06]; Models comparing the 7th grade early users group to the late users group: B in g8, math. -0.11 SD [95% CI -0.23, -0.001]; g8 Italian -0.15 SD [95% CI -0.26, -0.05]; Models comparing the 8th grade early users group to the late users group: B in g8, math. -0.02 SD [95% CI -0.12, -0.09]; g8 Italian -0.11 SD [95% CI -0.21, -0.01]. Model df for all Italian and math. = 5

Figure A7 – Effect of early use of social networks on additional academic outcomes

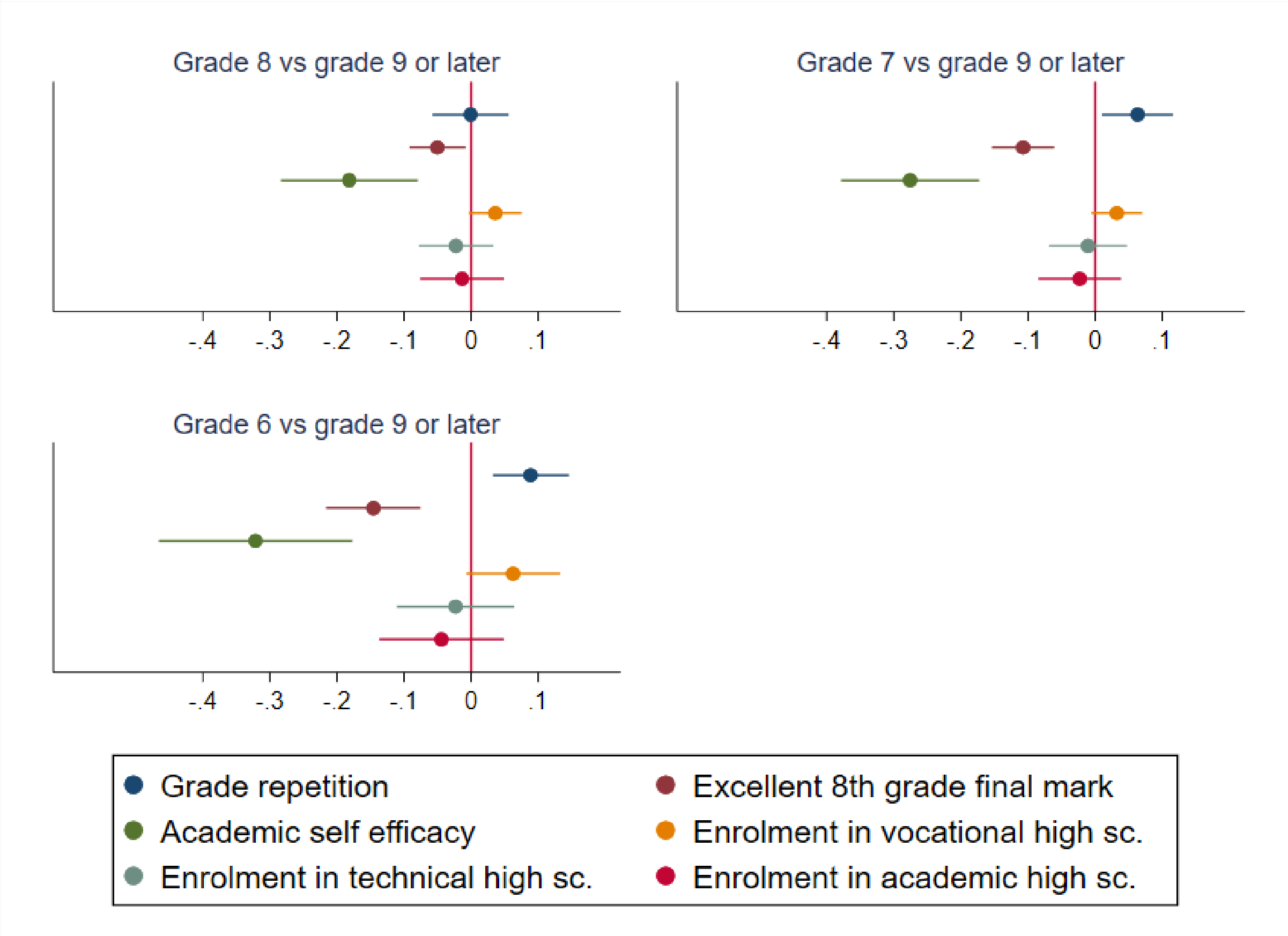

Note: data are presented as linear probability model coefficient estimates (entropy balancing weights used), expressed as differences in outcome probability, except academic self-efficacy, which is expressed in terms of standard deviations, from two-sided tests. Standard errors clustered at the school level. N (students) for models on marks, self-efficacy and enrolment: 1,956 for models on 6th graders, 1,797 for models on 7th graders, 1,511 for models on 8th graders. N for models on grade repetition (estimated on the 2007 cohort only): 965 for models on 6th graders, 870 for models on 7th graders, 723 for models on 8th graders. Excellent 8th grade final mark: g8 vs g9 B -0.05 [95% CI -0.09, -0.01], g7 vs g9 B -0.11 [95% CI -0.15, -0.06]; g6 vs g9 B -0.15 [95% CI -0.22, -0.08]; Enrolment in vocational high school: g8 vs g9 B 0.04 [95% CI -0.003, 0.08], g7 vs g9 B 0.03 [95% CI -0.01, 0.07]; g6 vs g9 B 0.06 [95% CI -0.01, 0.13]; Enrolment in technical high school: g8 vs g9 B -0.02 [95% CI -0.08, 0.03], g7 vs g9 B -0.01 [95% CI -0.07, 0.05]; g6 vs g9 B -0.02 [95% CI -0.11, 0.06]; Enrolment in academic high school: g8 vs g9 B -0.01 [95% CI -0.08, 0.05], g7 vs g9 B -0.02 [95% CI -0.08, 0.04]; g6 vs g9 B -0.04 [95% CI -0.14, 0.05]; Academic self-efficacy: g8 vs g9 B -0.21 [95% CI -0.33, -0.09], g7 vs g9 B -0.32 [95% CI -0.45, -0.20]; g6 vs g9 B -0.38 [95% CI -0.55, -0.21]; Grade repetition: g8 vs g9 B -0.001 [95% CI -0.06, 0.06], g7 vs g9 B 0.06 [95% CI 0.01, 0.12]; g6 vs g9 B 0.09 [95% CI 0.03, 0.15].

Figure A8 – Association between potential mediators and academic competences in grade 10

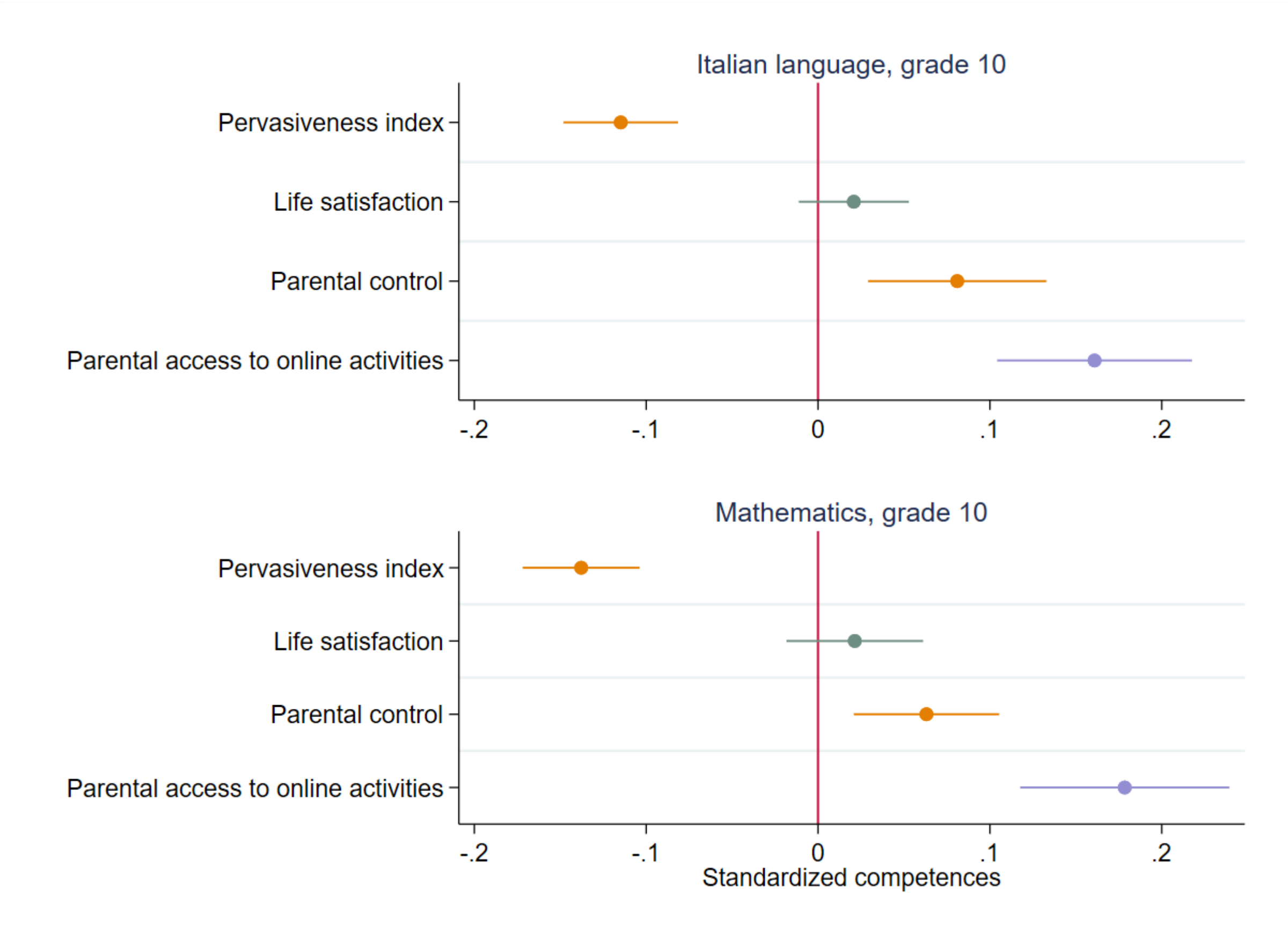

Note: data are presented as linear model coefficient estimates, expressed in terms of standard deviations in scores, from two-sided tests. N (students): 4,383 for the model on mathematics and 4,414 for the model on Italian. Models estimated separately for each outcome and potential mediator. Df of the models for maths and Italian = 11. Italian: Pervasiveness index: B -0.11 [95% CI -0.15, -0.08], Life satisfaction: B 0.02 [95% CI -0.01, 0.05]; Parental control: B 0.08 [95% CI 0.03, 0.13]; Parental access to online activities: B 0.16 [95% CI 0.10, 0.22]. Maths: Pervasiveness index: B -0.14 [95% CI -0.17, -0.10], Life satisfaction: B 0.02 [95% CI -0.02, 0.06]; Parental control: B 0.06 [95% CI 0.02, 0.11]; Parental access to online activities: B 0.18 [95% CI 0.12, 0.24].

Figure A9 – Association between potential mediators and years of exposure to social media by grade 10

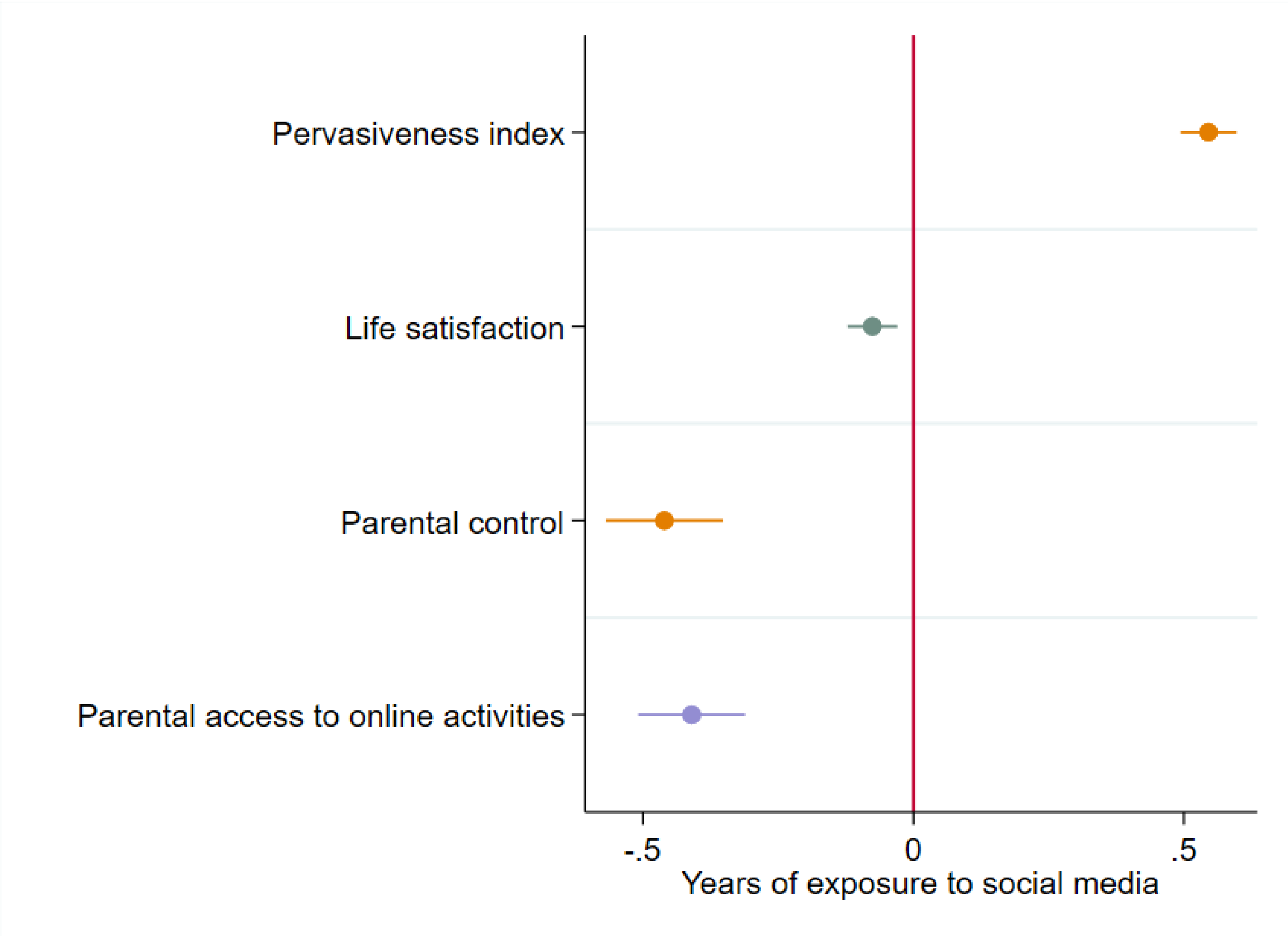

Note: data are presented as linear model coefficient estimates (entropy balancing weights used), expressed in years, from two-sided tests. Standard errors clustered at the school level; N (students): 4,589. Models estimated separately for each potential mediator. Df of the model = 12. Pervasiveness index: B 0.55 [95% CI 0.49, 0.60], Life satisfaction: B -0.08 [95% CI -0.12, -0.03]; Parental control: B -0.46 [95% CI -0.57, -0.35]; Parental access to online activities: B -0.41 [95% CI -0.51, -0.31],

Figure A10 – Effect of early use of social networks on competences in grades 8 and 10. 2007 cohort only.

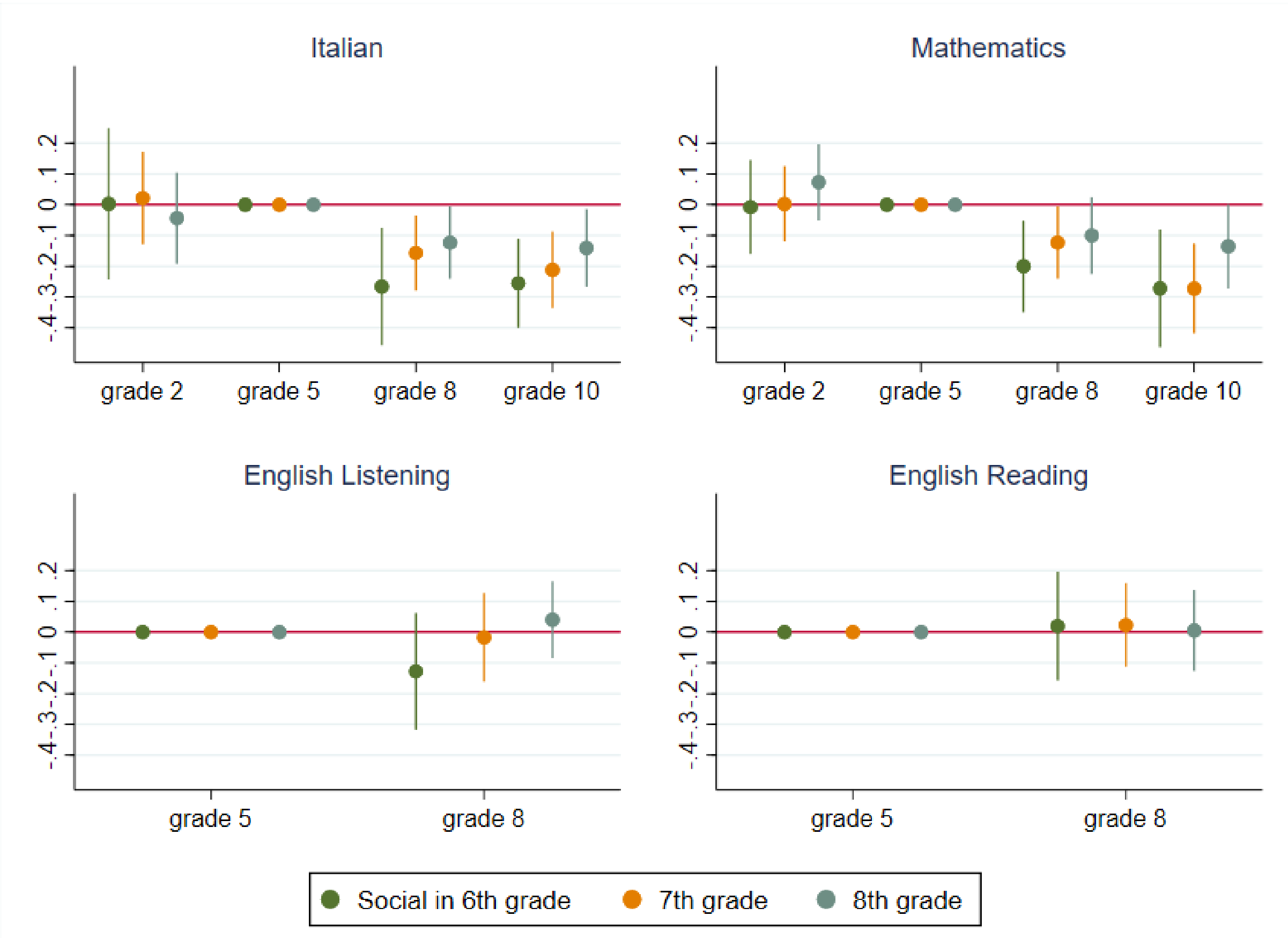

Note: results are presented as regression coefficient estimates, with error bars indicating 95% confidence intervals from two-sided tests. The coefficients display the interaction parameters between treatment status and grade from student fixed-effects models. Matching method: entropy balancing. Standard errors clustered at the individual level. N refers to student–time unit observations in all models. N in models comparing the 6th grade early users group with the late users group: 3,846 obs. in math. and Italian; 1,938 obs. in Eng. reading and listening. N in models comparing the 7th grade early users group with the late users group: 3,408 obs. in math. and Italian; 1,732 obs. in Eng. reading and listening. N in models comparing the 8th grade early users group with the late users group: 2,828 obs. in math. and Italian; 1,432 obs. in Eng. reading and listening. Models comparing the 6th grade early users group to the late users group: B in g8, math. -0.20 SD [95% CI -0.35, -0.05], g10 math. -0.27 SD [95% CI -0.46, -0.08], B in g8 Italian. -0.27 SD [95% CI -0.46, -0.07], g10 Italian. -0.26 SD [95% CI -0.40, -0.11]; g8 Eng. reading 0.02 SD [95% CI -0.16, 0.20]; g8 Eng list. -0.12 SD [95% CI -0.32, 0.06]; Models comparing the 7th grade early users group to the late users group: B in g8, math. -0.12 SD [95% CI -0.24, -0.01], g10 math. -0.27 SD [95% CI -0.42, -0.13], B in g8 Italian -0.16 SD [95% CI -0.30, -0.04], g10 Italian. -0.21 SD [95% CI -0.34, -0.09]; g8 Eng. reading 0.02 SD [95% CI -0.11, 0.16]; g8 Eng list. -0.02 SD [95% CI -0.16, 0.13]; Models comparing the 8th grade early users group to the late users group: B in g8, math. -0.10 SD [95% CI -0.22, 0.02], g10 math. -0.14 SD [95% CI -0.27, 0.001], B in g8 Italian -0.12 SD [95% CI -0.24, -0.01], g10 Italian. -0.14 SD [95% CI -0.27, -0.01]; g8 Eng. reading 0.01 SD [95% CI -0.13, 0.14]; g8 Eng list. 0.04 SD [95% CI -0.09, 0.17]. Model df for all Italian and math. = 7; df for Eng. = 3

Figure A11 – Effect of early use of social networks on competences in grades 8 and 10 in Italian and mathematics, alternative matching specifications

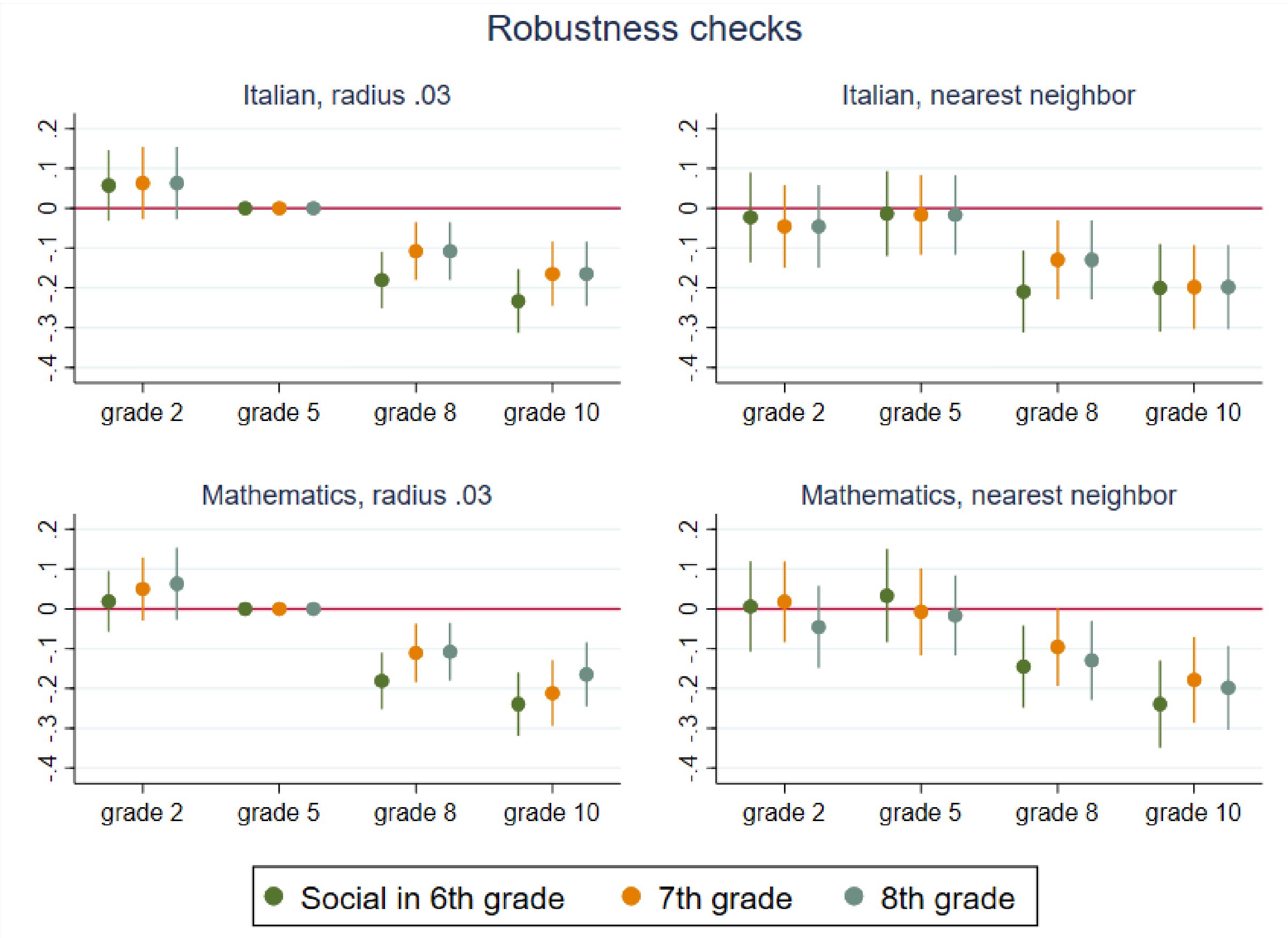

Note: results are presented as regression coefficient estimates, with error bars indicating 95% confidence intervals from two-sided tests. The coefficients display the interaction parameters between treatment status and grade from student fixed-effects models. N refers to student–time unit observations in all models. Models using nearest neighbor matching: matched-pairs fixed-effects models. Errors clustered at the individual level. N refers to student–time unit observations in all models. N in models comparing the 6th grade early users group with the late users group: radius matching 7,570 obs.; nearest neighbor matching 4,157 obs. N in models comparing the 7th grade early users group with the late users group: radius matching 6,955 obs.; nearest neighbor matching 4,827 obs. N in models comparing the 8th grade early users group with the late users group: radius matching 5,874 obs.; nearest neighbor matching 4,911 obs. Models comparing the 6th grade early users group to the late users group: B in g8, math. (Radius .03) -0.15 SD [95% CI -0.25, -0.04], g10 math. (Radius .03) -0.24 SD [95% CI -0.35, -0.13], B in g8, math. (Nearest neighbor.) -0.19 SD [95% CI -0.25, -0.11], g10 math. (Nearest neighbor.) -0.24 SD [95% CI -0.32, -0.16]; B in g8 Italian. (Radius .03) -0.21 SD [95% CI -0.31, -0.11], g10 Italian. (Radius .03) -0.20 SD [95% CI -0.31, -0.09], B in g8 Italian. (Nearest neighbor.) -0.18 SD [95% CI -0.25, -0.11], g10 Italian. (Nearest neighbor.) -0.23 SD [95% CI -0.31, -0.15]; Models comparing the 7th grade early users group to the late users group: B in g8, math. (Radius .03) -0.10 SD [95% CI -0.19, 0.001], g10 math. (Radius .03) -0.18 SD [95% CI -0.29, -0.07], B in g8, math. (Nearest neighbor.) -0.11 SD [95% CI -0.18, -0.04], g10 math. (Nearest neighbor.) -0.21 SD [95% CI -0.29, -0.13]; B in g8 Italian. (Radius .03) -0.13 SD [95% CI -0.23, -0.03], g10 Italian. (Radius .03) -0.20 SD [95% CI -0.30, -0.09], B in g8 Italian. (Nearest neighbor.) -0.11 SD [95% CI -0.18, -0.04], g10 Italian. (Nearest neighbor.) -0.16 SD [95% CI -0.25, -0.08]; Models comparing the 8th grade early users group to the late users group: B in g8, math. (Radius .03) -0.04 SD [95% CI -0.14, 0.06], g10 math. (Radius .03) -0.13 SD [95% CI -0.23, -0.03], B in g8, math. (Nearest neighbor.) -0.09 SD [95% CI -0.17, -0.01], g10 math. (Nearest neighbor.) -0.13 SD [95% CI -0.22, -0.05]; B in g8 Italian. (Radius .03) -0.09 SD [95% CI -0.19, 0.004],

g10 Italian. (Radius .03) -0.11 SD [95% CI -0.21, -0.01], B in g8 Italian. (Nearest neighbor.) -0.08 SD [95% CI -0.16, -0.01], g10 Italian. (Nearest neighbor.) -0.11 SD [95% CI -0.19, -0.02]. Model df for both Italian and math. = 8

Figure A12 – Effect of early use of social media on competences in grades 8 (Italian, mathematics and English) and 10 (Italian and mathematics), with the inclusion of parental control variables among the matching covariates

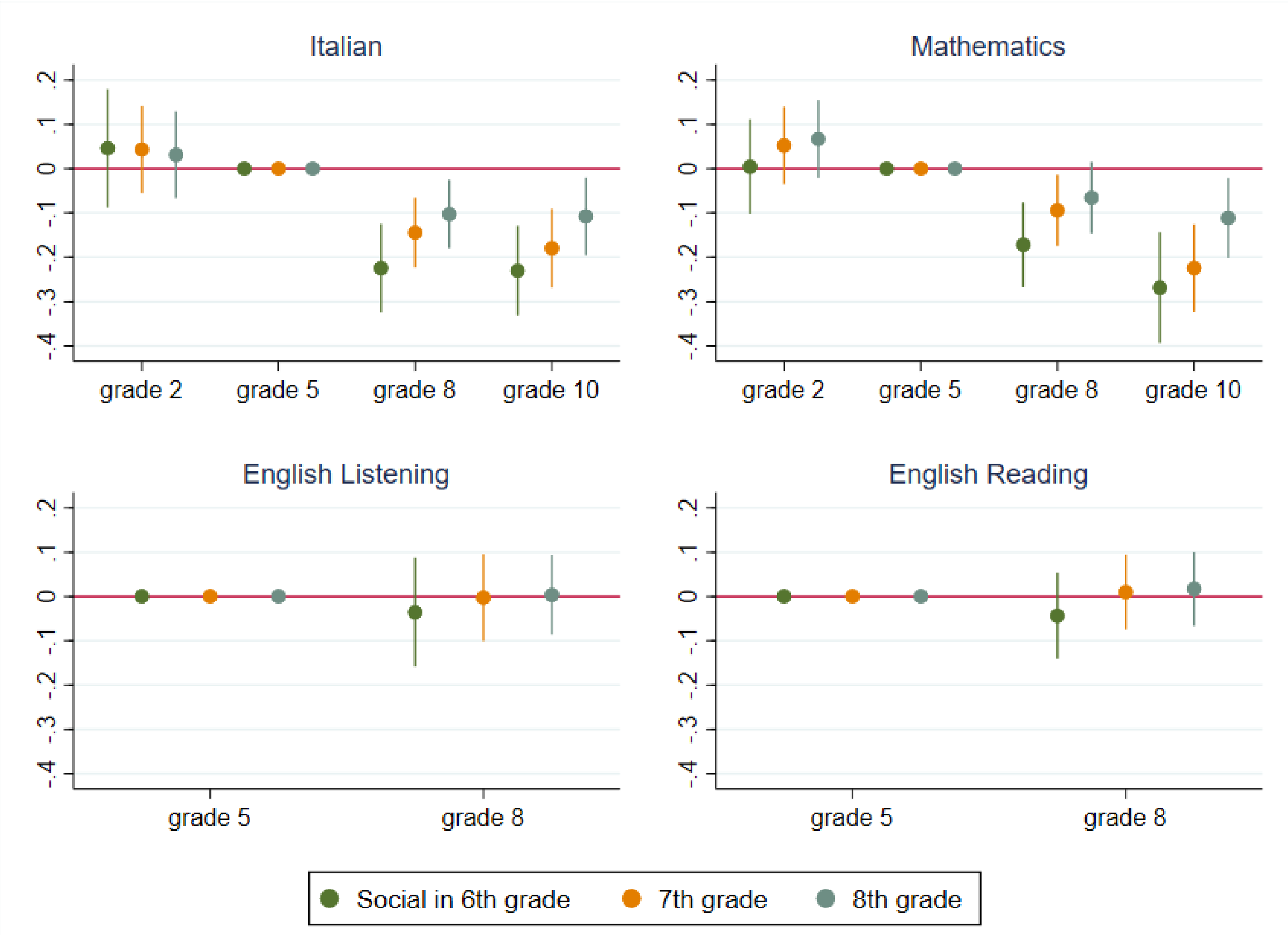

Note: see Figure 2 for model specifications and sample sizes. Parental control variables include: whether or not the student was subject to parental control measures (parental control tools or internet activity control) and the grade at which these controls were implemented. Models comparing the 6th grade early users group to the late users group: B in g8, math. -0.18 SD [95% CI -0.28, -0.08], g10 math. -0.27 SD [95% CI -0.40, -0.15], B in g8 italian. -0.23 SD [95% CI -0.34, -0.13], g10 Italian. -0.24 SD [95% CI -0.34, -0.13]; g8 Eng. reading 0.05 SD [95% CI -0.15, 0.05]; g8 Eng list. -0.04 SD [95% CI -0.17, 0.08]; Models comparing the 7th grade early users group to the late users group: B in g8, math. -0.09 SD [95% CI -0.17, -0.01], g10 math. -0.22 SD [95% CI -0.32, -0.12], B in g8 Italian -0.14 SD [95% CI -0.22, -0.06], g10 Italian. -0.17 SD [95% CI -0.26, -0.08]; g8 Eng. reading 0.01 SD [95% CI -0.07, 0.10]; g8 Eng list. -0.003 SD [95% CI -0.10, 0.09]; Models comparing the 8th grade early users group to the late users group: B in g8, math. -0.07 SD [95% CI -0.15, 0.01], g10 math. -0.11 SD [95% CI -0.20, -0.02], B in g8 Italian -0.10 SD [95% CI -0.18, -0.03], g10 Italian. -0.11 SD [95% CI -0.20, -0.02]; g8 Eng. reading 0.02 SD [95% CI -0.07, 0.10]; g8 Eng list. -0.000 SD [95% CI -0.09, 0.09]. Model df for all Italian and math. = 7; df for Eng. = 3

Figure A13 – Equivalence between groups in parental frequency of access to online school register; matching technique: entropy balancing.

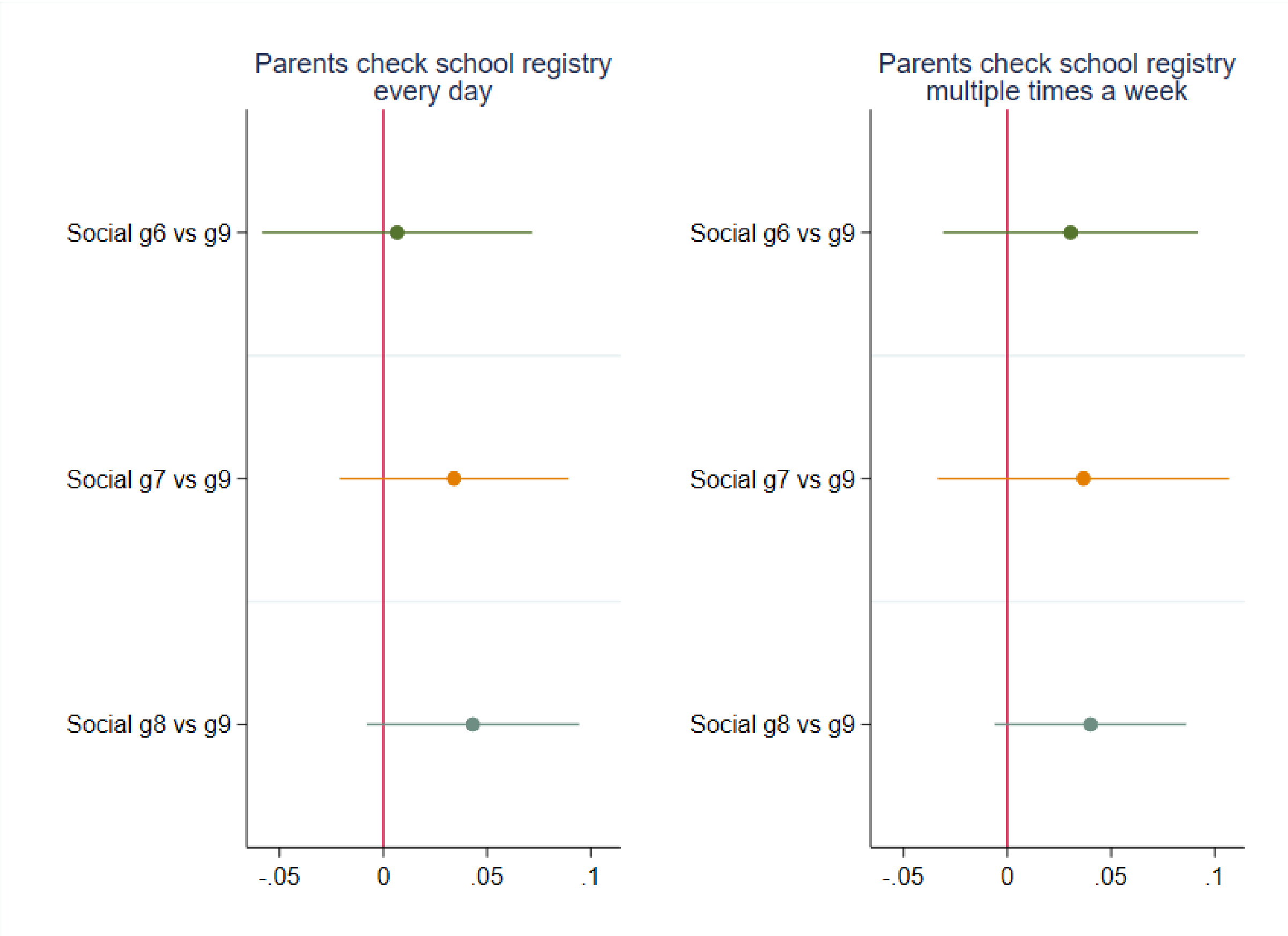

Note: data are presented as linear probability model coefficient estimates, expressed as differences in outcome probability from two-sided tests. Matching method: entropy balancing. Standard errors clustered at the school level. N (students): 1,511 for models on 8th graders; 1,797 for models on 7th graders and 1,956 for models on 6th graders. g8 vs g9 (every day) B 0.4 [95% CI -0.01, 0.09], g8 vs g9 (Multiple times week) 0.04 [95% CI -0.01, 0.09]; g7 vs g9 (every day) 0.03 [95% CI -0.02, 0.09], g7 vs g9 (Multiple times week) 0.04 [95% CI -0.03, 0.11]; g6 vs g9 (every day) 0.01 [95% CI -0.06, 0.07], g6 vs g9 (Multiple times week) 0.03 [95% CI -0.03, 0.09].

Table A1 - Sample representativeness

Track	All selected provinces	EYES UP starting sample	EYES UP final sample
Academic	51	47	49
Technical	33	33	33
Vocational	16	20	18
Total	100	100	100
Total (N students)	99,815	7,083	6,150

Note: we had to exclude students enrolled in three-year vocational programs courses (459 students in our final sample), that are not present in national registers from this table. Starting sample: students enrolled in the sampled classrooms. Final sample: respondents.

Table A2 - Congruence between effect estimated on different samples

Comparison<br>(1)	Effect estimated at grade<br>(2)	Estimated effect<br>(3)	Difference in the effects against "late users"<br>(4)	Difference in point estimates<br>(5)
		Italian		
7th vs 8th grade	8	-0,040	-0,034	-0,006
	10	-0,092	-0,060	-0,032
6th vs 8th grade	8	-0,130	-0,116	-0,014
	10	-0,188	-0,115	-0,073
6th vs 7th grade	8	-0,090	-0,082	-0,008
	10	-0,059	-0,055	-0,004
		Mathematics		
7th vs 8th grade	8	-0,032	-0,026	-0,007
	10	-0,095	-0,104	0,009
6th vs 8th grade	8	-0,109	-0,105	-0,004
	10	-0,125	-0,162	0,037
6th vs 7th grade	8	-0,081	-0,079	-0,001
	10	-0,042	-0,058	0,016

Note: all effects estimated using entropy balancing matching by means of student fixed-effect models (for model specifications: see Figure 2 in the Main manuscript). Column 3 reports the effects of the comparison between the groups described in column 1, at the grade specified in column 2. Column 4 reports the difference between the effects estimated against late users of the two groups described in column 1 (e.g. the effect of 8th grade against late users minus the effect of 7th grade against late users). Column 5 is calculated by subtracting column 3 from column 4 and highlights the similarity of results by means of different and independent estimation methods.

Supplementary Information 2 – Scales

2.1 Smartphone Pervasiveness Scale for Adolescents (SPS-A)

2.1.1 Literature

Smartphone pervasiveness in students' daily lives was measured through the extended Smartphone Pervasiveness Scale for Adolescence (SPS-A), which was first validated on a sample of 3,289 Italian upper secondary school students[1]. The scale assesses the subjective frequency of smartphone use in key daily-life moments that could affect adolescents' social and physiological well-functioning (e.g. during mealtimes with family, while spending time with friends and on waking up). When assessing non-pathological problematic smartphone use, the SPS-A has been proved to be both a valuable alternative to smartphone addiction scales and a good predictor of smartphone duration of use with respect to self-reported measures[2]. Students were asked to report on a five-point Likert scale ranging from "Never" to "Always" (*Never* = *1, Rarely* = *2, Sometimes* = *3, Often* = *4, Always* = *5*) how frequently they usually make use of their smartphones during seven key moments of the day (see Table A3).

2.1.2 Reliability and construct validity

In terms of inter-item reliability, the items included in the analysis displayed an acceptable degree of internal consistency (Cronbach's alpha = 0.74). Corrected item-total correlations, i.e. the relationship between single items and the overall scale when the concerned item is removed, were investigated: the values were all positive and above the 0.3 threshold.

Construct validity was assessed by means of a Confirmatory Factor Analysis (CFA) on the entire sample, verifying the unidimensionality of the latent construct, i.e., Smartphone Pervasiveness. The Weighted Least Square Mean and Variance estimation method (WLSMV) was adopted, being one of the best performing estimators when dealing with ordinal data[3]. In accordance with the rules of thumb provided in the literature[4,5,6], the model specification results in an acceptable fit (RMSEA = 0.072 [0.068-0.076], SRMR = 0.047, CFI = 0.936, TLI = 0.915). The standardized factor loadings were all positive, significant and above the 0.4 threshold[4] (see Table A4 for further details).

2.1.3 Items and computation

The pervasiveness index is computed by taking the mean of the nine items composing the SPS-A scale.

Table A3 - Smartphone Pervasiveness Scale for adolescents (SPS-A); *Italian version in brackets*

How often do you find yourself using your smartphone during the following everyday situations? (*Quanto spesso ti capita di usare lo smartphone durante le seguenti situazioni quotidiane?*)
1 - During meals with my family (*Durante i pasti con i miei familiari*)<br>2 - While I'm with friends (*Mentre sono con gli amici*)<br>3 - While doing homework (*Durante lo svolgimento dei compiti a casa*)<br>4* - The evening before falling asleep (*La sera prima di prendere sonno*)<br>5 - At night if I wake up (*Di notte se mi sveglio*)<br>6 - In the morning as soon as I wake up (*La mattina appena mi sveglio*)<br>7 - During lessons at school, including when we are not supposed to be using devices (*Durante le lezioni a scuola, anche quando non è previsto l'utilizzo di dispositivi*)<br>8 - While watching a series, a film or TV (*Mentre guardo una serie, un film o la TV*)<br>9* - While doing physical activities (e.g. sports, trips, housework) (*Mentre svolgo attività di movimento (es. sport, gite, lavori in casa)*)

*Items added to the SPS-A scale.

Table A4 - Descriptive Statistics, factor loadings of the one-factor CFA model and factorial validity of the SPS-A latent construct.

Item	n	Mean	Std dev	Skewness	Excess kurtosis	Factor Loadings
1	6609	1.877	1.074	1.148	0.527	0.475
2	6609	3.03	0.912	0.047	-0.338	0.470
3	6609	3.299	1.031	-0.284	-0.352	0.531
4	6609	4.271	1.072	-1.571	1.744	0.676
5	6609	2.234	1.427	0.830	-0.713	0.664
6	6609	3.462	1.416	-0.381	-1.214	0.599
7	6609	2.664	1.174	0.200	-0.811	0.489
8	6609	2.921	1.179	0.007	-0.875	0.530
9	6609	2.037	1.164	0.922	-0.136	0.444
Fit Indices (WLSMV estimation method) – one factor CFA						
2	df	p-value	RMSEA	CFI	TLI	SRMR
958.67	27	0.000	0.072 [0.068-0.076]	0.936	0.915	0.047

2.2 Subjective well-being

2.2.1 Literature

The Subjective well-being scale was first developed to assess one of the seven core theoretical dimensions of psychological well-being, measured by means of the Comprehensive Inventory of Thriving for Children (CTI)[7]. Subjective well-being has been defined as "an internal barometer of how life is going" and it is articulated in 3 facets, i.e. life satisfaction, positive feelings and negative feelings. Each subscale is defined by 3 indicators answered on a 5 point-Likert scale ranging from "Strongly disagree" to "Strongly agree" (*Strongly disagree = 1, Disagree = 2, Neither agree nor disagree = 3, Agree = 4, Strongly agree = 5*) (see Table A5). The psychometric features of the Italian adaptation of the CIT have been evaluated in previous research[9], providing evidence of the validity and reliability of the instrument.

2.2.2 Reliability and construct validity

Concerning inter-item reliability, the items included in the analysis displayed a good/excellent degree of internal consistency with respect to both the total SWB scale (Cronbach's alpha = 0.91) and the single subscales (Life satisfaction Cronbach's alpha = 0.85, Positive feelings Cronbach's alpha = 0.90, Negative feelings Cronbach's alpha = 0.83). Corrected item-total correlations values with respect to both the total scale and the subscales were all positive and above the 0.3 threshold.
The three facets of psychological well-being are assumed to be unidimensional and distinguishable from each other: a CFA was conducted on the whole sample to test the posited factor structure. A 3-factor model was specified: single items loaded on the hypothesized latent variable and factors were allowed to correlate with each other. The Weighted Least Square Mean and Variance estimation method (WLSMV) was adopted, being one of the best performing estimators when dealing with ordinal data[3] . Missing values were treated through listwise deletion, i.e., removing individual records with missing values from the analysis. In accordance with the rules of thumb provided in the literature[4,5,6] the model specification results in an acceptable fit (RMSEA = 0.065 [0.061-0.069], SRMR = 0.019, CFI = 0.995, TLI = 0.993). The standardized factor loadings were all positive, significant and above the 0.4 threshold[3] (see Table A6). Factor correlations were all below the 0.8 threshold ([0.62 – 0.79] range), supporting the notion that the three subscales represent related yet distinguishable latent constructs[3].

2.2.3 Scale computation

As reported in Appendix A on the website of the first author of the CIT development and validation paper, the score on each subscale is computed by averaging the responses across three items on the scale.

Table A5 - Subjective well-being; *Italian version in brackets*

Please indicate your agreement or disagreement with each of the following statements using the scale below (1 Strongly Disagree, 2 Disagree, 3 Neither Agree nor Disagree, 4 Agree, 5 Strongly Agree). (*Indica quanto sei d'accordo con ciascuna delle seguenti frasi, selezionando la tua scelta sulla seguente scala che va da 1 "Per niente d'accordo" a "Molto d'accordo"*).
1 - In most ways my life is close to my ideal (*In molte cose la mia vita è come la vorrei*)<br>2 - I am satisfied with my life (*Sono contento/a della mia vita*)<br>3 - My life is going well (*La mia vita va bene*)<br>4 - I feel positive most of the time (*La maggior parte delle volte mi sento contento/a*)<br>5 - I feel happy most of the time (*La maggior parte delle volte mi sento felice*)<br>6 - I feel good most of the time (*La maggior parte delle volte mi sento di buon umore*)<br>7 - I feel negative most of the time (*La maggior parte delle volte mi sento triste*)<br>8 - I experience unhappy feelings most of the time (*La maggior parte delle volte mi sento infelice*)<br>9 - I feel bad most of the time (*La maggior parte delle volte mi sento di cattivo umore*)

Table A6. Descriptive Statistics, factor loadings of the 3-factor CFA model.

Item	n	Mean	Std dev	Skewness	Excess kurtosis	Subscale	Factor Loadings
1	6599	3.334	1.050	-0.396	-0.516	Life satisfaction	0.750
2	6587	3.653	1.057	-0.703	-0.064		0.922
3	6551	3.664	1.051	-0.697	-0.031		0.887
4	6585	3.473	1.037	-0.429	-0.375	Positive feelings	0.928
5	6584	3.422	1.046	-0.373	-0.44		0.939
6	6588	3.355	1.035	-0.324	-0.455		0.841
7	6581	3.322	1.037	-0.344	-0.475	Negative feelings	0.867
8	6577	3.488	1.098	-0.424	-0.549		0.889
9	6601	3.195	1.144	-0.234	-0.749		0.748
Fit Indices (WLSMV estimation method) – three factors CFA							
2	df	p-value	RMSEA	CFI	TLI		SRMR
674.946	24	0.000	0.065 [0.061-0.069]	0.995	0.993		0.019

2.3 Self-efficacy

2.3.1 Literature

Perceived self-efficacy is defined as the "belief in one's capabilities to organize and execute the courses of action required to produce given attainments[9]. Self-efficacy is supposed to play a general role on mental health, determining how people feel, think,

motivate themselves and behave and therefore sustaining personal accomplishments, reducing stress and lowering vulnerability to depression and anxiety[10,11]. It has been posited that three different domains of self-efficacy are involved in the regulation of negative affect: academic, social and emotional self-efficacy[12]. Academic self-efficacy refers to the individual's perceived ability to control their learning behaviours, master subjects and meet educational expectations. Social self-efficacy refers to the individual's perceived ability to be authentic and assertive in peer relationships. Emotional self-efficacy refers to the individual's perceived ability to cope with negative emotions[12]. The self-efficacy questionnaire for children was developed to account for the above-mentioned domains, each of which was originally defined by eight items. Due to optimization reasons related to the length of the questionnaire, not all the original items were adopted in the questionnaire. First, one item per subscale was removed considering Muris's[12] EFA, which provided evidence that some of the items considered did not indeed load convincingly on their intended factor. Second, of the seven remaining items per domain, four were selected and implemented in the questionnaire. The choice relied on the combination of three main criteria: i) research objectives, ii) avoidance of overlapping items, i.e., items similar in meaning items and iii) the higher factor loadings value according to Muris[12].

The final 12-item scale was measured on a 5-point Likert scale, with values ranging from "Not at all" to "Very well" (*Not at all = 1, Very well = 5*).

2.3.2 Reliability and construct validity

Analysis of the inter-item reliability of subscales suggested removing one item from the social self-efficacy subscale and one item from the emotional self-efficacy subscale, as the two items were both associated with an increase in reliability following their respective drop. To produce further evidence supporting the removal of the two items, an exploratory analysis was conducted to investigate item clustering in greater detail. The sample was randomly divided into two halves: EFA was conducted on the first half, while CFA was then implemented on the second half. According to the literature, parallel analysis suggested the retention of three factors, which were extracted by means of Principal Axis Factoring and obliquely rotated (oblimin rotation). As both the items concerned did not load convincingly on the intended factor, the research team agreed on their removal. Following the above-mentioned adjustment, the indicators displayed acceptable/good degree of internal consistency with respect to both the total self-efficacy scale (Cronbach's alpha = 0.71) and the single subscales (academic self-efficacy Cronbach's alpha = 0.74, social self-efficacy Cronbach's alpha = 0.63, emotional self-efficacy Cronbach's alpha = 0.78). Corrected item-total correlations values with respect to both the total scale and the subscales were all positive and above the 0.3 threshold.

The three self-efficacy domains are assumed to be unidimensional and distinguishable from each other: a CFA was conducted on the whole sample to test the posited factor structure. A 3-factor model was specified: single items loaded on the hypothesized latent variable and factors were allowed to correlate with each other. The Weighted

Least Square Mean and Variance estimation method (WLSMV) was adopted, being one of the best performing estimators when dealing with ordinal data[3]. In accordance with the rules of thumb provided in the literature[4,5,6] the model specification results in an acceptable fit (RMSEA = 0.056 [0.052-0.060], SRMR = 0.035, CFI = 0.977 , TLI = 0.968). The standardized factor loadings were all positive, significant and above the 0.4 threshold[3] (see Table A8 for further details). Factor correlations were all far below the 0.8 threshold ([0.12 – 0.51] range), supporting the notion that the three subscales represent distinguishable latent constructs[3].

2.3.3 Scale computation

The score on each subscale is computed by averaging the responses across the items on the scale.

Table A7 - Self-efficacy; *Italian version in brackets*

How well can you… (1 - “Not at all”, 5 - “Very well”) *(Indica con un valore compreso tra 1 “Per niente” e 5 “Molto”, scegliendo la risposta appropriata per ciascun elemento. Quanto riesci a…)*
1 – Can you study when there are other interesting things to do? (*Studiare quanto ci sono altre cose interessanti da fare?*)<br>2 – Can you express your opinions when other classmates disagree with you? (*Esprimere le tue opinioni quando altri compagni di classe non sono d’accordo con te?*)<br>3 – Do you succeed in cheering yourself up when an unpleasant event has happened? (*Tirarti su di morale quando è accaduto un evento spiacevole?*)<br>4 – Can you study a chapter for a test? (*Studiare un capitolo per una verifica-interrogazione?*)<br>5 – Can you become friends with other children? (*Fare amicizia con gli altri coetanei?*)<br>6 – Do you succeed in suppressing unpleasant thoughts? (*Scacciare pensieri spiacevoli?*)<br>7 – Do you succeed in finishing all your homework every day? (*Finire tutti i tuoi compiti ogni giorno?*)<br>9 – Can you give yourself a pep talk when you feel low (*Darti una carica positiva quando ti senti giù?*)<br>10 – Can you pay attention during every class? (*Prestare attenzione durante ogni lezione?*)<br>11 – Can you tell other children that they are doing something that you don’t like? (*Dire ai tuoi coetanei che stanno facendo qualcosa che non ti piace?*)

Removed items:
Social self-efficacy subscale: 8 – Can you work in harmony with your classmates? (*Lavorare in armonia con i tuoi compagni di classe?*)
Emotional self-efficacy subscale: 12 – Do you succeed in not worrying about things that might happen? (*Vivere le cose che potrebbero accadere senza preoccupazione?*)

Table A8 - Descriptive Statistics, factor loadings of the 3-factor CFA model.

Item	n	Mean	Std dev	Skewness	Excess kurtosis	Subscale	Factor Loadings
1	6609	2.47	1.06	0.37	-0.43	Academic self-efficacy	0.643
4	6609	3.47	1.16	-0.44	-0.62		0.717
7	6609	3.00	1.34	0.00	-1.19		0.705
10	6609	3.03	1.06	-0.06	-0.60		0.655
2	6609	3.30	1.27	-0.22	-1.00	Social self-efficacy	0.666
5	6609	3.41	1.15	-0.32	-0.71		0.605
11	6609	3.25	1.21	-0.20	-0.88		0.659
3	6609	2.98	1.21	0.04	-0.91	Emotional self-efficacy	0.759
6	6609	2.71	1.18	0.27	-0.78		0.684
9	6609	2.95	1.20	0.06	-0.86		0.856
Fit Indices (WLSMV estimation method) – three factors CFA							
2	df	p-value	RMSEA	CFI	TLI	SRMR	
695.467	32	0.000	0.056 [0.052-0.060]	0.977	0.968	0.035	

Supplementary Information 3 – Robustness checks

This section details the robustness checks summarized in the main paper (section: Robustness checks). One approach to relax the parallel trends assumption is to assume it holds conditional on covariates, implying that it is valid among subpopulations of "matched" treated and untreated units characterized by similar values of a covariate vector. In our research, the goal is pursued computing a set of weights by means of entropy balancing to improve covariates balance between early and late users, reinforcing the plausibility of a conditional independence assumption (of treatment from potential outcomes) on changes. Another source of potential bias concerns the exclusive reliance on a single matching algorithm. To address this, we implemented three alternative matching procedures, as detailed in the previous section: entropy balancing, nearest neighbor matching (without replacement, caliper = 0.05), and radius matching (caliper = 0.03). The results of these robustness checks are presented for Italian and mathematics in Figure A11. The robustness checks largely confirm our main results: early social media use is associated with negative, statistically significant and substantial effects on competence scores, which tend to increase from grade 8 to grade 10. However, the relationship between the magnitude of the effect and the number of years of exposure is less clearly defined, particularly when comparing students who opened accounts in grades 7 and 8.

A common criticism of statistical matching is that it only accounts for observable characteristics, raising concerns about unobserved confounding. For example, families that allow their children to open a social media account earlier may also be less engaged in their children's education or in their leisure time. To explore this possibility, we examined parental involvement using two proxies: i) parental control activities (presence/absence of parental control strategies and, if present, until which

age; parents' access to online activities and, if present, until which age) and ii) the frequency with which parents accessed the electronic school register, as reported by students. These indicators have been collected at the moment of the interview only, so we are aware that they are technically "bad controls" if we want to use them at the same level of analysis of the variables used for matching (that refer to invariant characteristics or to behaviors/outcomes observed before grade 6): in fact, we might hypothesize that early exposed students are controlled more frequently (than they would have been otherwise) by virtue of a lower school performance induced by the early opening of social media.

Concerning parental control and access to online activity, we know from the literature (and confirmed in our sample in Table 1 in the Main manuscript) that families more aware of the risks connected to the internet impose more limitations on their offspring, and that this behaviour is correlated with competences (as seen in Figure A8). We did not include these variables in the main matching strategy, since parental control behavior might well reflect the evolution of the awareness of parents over time as a consequence of the effects of social media exposure. Since we do not have longitudinal data on this practice, we cannot disentangle the two possible behaviors. We nonetheless imposed this equivalence in parental control practices (both parental control of devices and parental access to online activities and age until which both practices were performed) on the matching algorithms. The results can be seen in Figure A12 and are virtually identical to those presented in the main specification.

As for electronic school registers, these platforms usually provide information on marks, absences, homework, and disciplinary notes and are always present in Italian secondary schools. In our sample, 37% of students reported that their parents accessed the register daily, and an additional 34% reported access several times a

week. Given that this activity is not directly related to the students' use of technology (contrarily to parental control or parental access), we opted for a balance test instead of a recalibration of the matching algorithm. For each comparison group, we regressed the frequency of parental access on the "treatment" indicator, using the same matching weights applied in the main analyses (see Figure A13). We found minimal and not statistically significant differences across groups. Interestingly, early social media users reported slightly higher parental monitoring – contrary to what one might expect if unobserved parental neglect was biasing the results in favor of late adopters.

Our identification strategy rests on the parallel trend assumption. Due to confounding, unobserved factors, we may still be unsure on whether the mean outcome of the treated population would have evolved in parallel with the mean outcome of the control population in the absence of treatment. It has become common practice to perform plausibility checks verifying the significance of pre-treatment differences in trends. Intuitively, the absence of differential pre-treatment trends between treatment and control groups supports the plausibility of the parallel trends assumption in post-treatment periods.

Therefore, we examined whether early and late adopters of social media exhibited comparable academic trajectories before exposure. As noted earlier, this test can be performed using grade 2 standardized assessments in mathematics and Italian (English competences are not tested in grade 2). These tests support the robustness of our findings with respect to our main outcome, competence scores.

However, verifying the existence of such pre-trends may be insufficient, as recent literature highlights the low power of these tests, the existence of statistical issues arising from conditioning the analysis on their results and, ultimately, the lack of clear guidance for researchers when relevant pre-trends are detected[13].

To address these limitations, our analysis adopts a recently developed approach for robust inference and sensitivity analysis[14], designed for empirical settings in which parallel trends may not strictly hold. The objective is to determine how large the post-treatment violation of parallel trends would have to be for the estimated causal effects on competences to lose statistical significance.

Following the logic behind pre-trend plausibility tests, potential post-treatment deviations from parallel trends are bounded using information on pre-treatment deviations.

In our case, taking grade 5 as the baseline, we estimate the pre-treatment deviation from parallel trends - denoted by δ_{-1} - by comparing differences in trends between grade 2 and 5 across treatment and control groups. We then impose restrictions on the magnitude of possible post-treatment deviations δ_1.

Specifically, we assume that the time varying confounding factors that drive non-parallel trends in competences are of similar magnitude before and after the treatment. Formally, the post-treatment bias is assumed to be at most $\overline{M}$ times the maximal pre-treatment bias.

The robust confidence sets Δ are defined as ($\delta \in \Delta^{RM}(\overline{M})\ for\ \overline{M} > 0$):

$$\Delta^{RM}(\overline{M}) = \left\{\delta\colon |\delta_{t+1} - \delta_t| \leq \overline{M} \times \max_{s<0}|\delta_{s+1} - \delta_s|,\ \forall\, t \geq 0\right\}$$

Ultimately, confidence sets for the causal effect that are uniformly valid under the imposed restrictions can be constructed using the conditional and hybrid methods[15]. It should be noted that the confidence sets are larger when the uncertainty concerning the estimate of the pre-trends is higher.

The resulting confidence sets are reported in Figures A3, A4 and A5, distinguishing the three comparison groups and the causal effects estimated for grades 8 and 10, for both mathematics and Italian. In each figure, the blue confidence

set represents the standard DiD estimate, while the red confidence sets correspond to those obtained under the robust DiD approach. The vertical axis displays the standardized competence scores, while the horizontal axis reports alternative values of $\overline{M}$. The larger the value of $\overline{M}$, the greater the violation of the parallel trends assumption that is being allowed. A "breakdown value" of $\overline{M}$ is identified as the smallest value at which the robust confidence set includes zero. This breakdown value indicates the relative magnitude of post-treatment deviations from parallel trends – expressed in terms of the estimated pre-trends – that would be sufficient to invalidate the conclusion of a statistically significant effect.

Figure A3 shows that when restricting the violation of parallel trends to be no larger than the estimated pre-trend (i.e., $\overline{M}= 1$), the negative causal effects of opening a social media account in 6th grade is statistically significant. The breakdown value is 1.5 for mathematics in grade 8 [95% CI -0.35, 0.03] as for Italian in both grades 8 [95% CI -0.43, 0.05] and 10 [95% CI -0.43, 0.05]. Hence, to overturn the conclusion of a significant negative effect, the violation of parallel trends in the post-treatment period would need to be 1.5 times larger than the estimated pre-trend deviation. The effect on mathematics competences in grade 10 is the most robust: even allowing the post-treatment violation to be twice as large as the pre-treatment deviation (i.e $\overline{M}= 2$), the causal effect is significantly different from zero [95% CI -0.50, -0.01].

Regarding early social media exposure in 7th grade (Figure A4), the results of the analysis are less robust to violations of the parallel trends assumption compared to those for 6th grade. The effect on mathematics competences in grade 8 loses significance even under mild deviations: when post-treatment trends deviate by half the estimated pre-trend ($\overline{M}= 0.5$), the robust confidence set already includes zero [95% CI -0.19, 0.02]. The breakdown value for Italian competences in grades 8 and

10 is slightly higher, around $\overline{M}= 1$ [95% CI -0.26, 0.03] in grade 8, [95% CI -0.29, 0.00] in grade 10). The most robust finding concerns the effect on mathematics competences in grade 10: if we are willing to restrict the violation of the parallel trends assumption in the post-treatment period to be no more than around 1.5 the pre-trend deviation, a null effect is ruled out [95% CI -0.40, 0.02] for $\overline{M}= 1.5$).

For the group opening social media accounts in 8th grade (Figure A5), the conclusion of significant effects are not robust once any relaxation of the parallel trends assumption is introduced. For all estimated causal effects, when allowing post-treatment deviations to be half the estimated pre-trend ($\overline{M}= 0.5$), the robust confidence sets include zero, implying that the results are highly sensitive to deviations from the identifying assumption [95% CI -0.17, 0.05] for mathematics in grade 8, 95% CI -0.22, 0.02 for mathematics in grade 10, 95% CI -0.19, 0.00 for Italian in grade 8, 95% CI -0.20, 0.01 for Italian in grade 10).

Finally, we tested the internal consistency of our estimates across different matched group comparisons. Specifically, when comparing two early-adopter groups (e.g., 6th vs. 7th grade), we expected the effect of social media exposure to be similar to the difference in effects observed when each group is compared separately with late adopters (9th grade or later). We matched these early-exposed groups directly to each other, following the same procedure outlined in the Methods section. The resulting estimates, reported in Table A2, are highly consistent across group comparisons, providing additional support for the validity of our identification strategy. In only one out of twelve tests is the difference in estimates higher than 0.05.

References

1. Gerosa, T., Gui, M. & Büchi, M. (2022). Smartphone Use and Academic Performance: A Pervasiveness Approach Beyond Addiction. *Social Science Computer Review*, *40*(6), 1542-1561

2. Chakraborty, S., Gui, M., Gerosa, T. & Marciano, L. (2024). Testing the validity of the smartphone pervasiveness scale for adolescents with self-reported objective smartphone use data. *DIGITAL HEALTH*, 10, 1-9

3. Brown, T. (2006). *Confirmatory factor analysis for applied research.* New York: Guildford

4. Hu, L. T. & Bentler, P. M. (1999). Cutoff criteria for fit indexes in covariance structure analysis: Conventional criteria versus new alternatives. *Structural equation modeling: a multidisciplinary journal*, 6(1), 1-55

5. Browne, M. W. & Cudek, R. (1993). Alternate ways of assessing model fit. In K. A. Bollen & J. S. Long (Eds.), *Testing structural equations models* (pp. 136-162). Newbury Park, CA: Sage

6. MacCallum, R. C., Browne, M. W. & Sugawara, H. M. (1996). Power analysis and determination of sample size for covariance structure modelling. *Psychological methods*, *1*(2), 130

7. Su, R., Tay, L. & Diener, E. (2014). The development and validation of the Comprehensive Inventory of Thriving (CIT) and the Brief Inventory of Thriving (BIT). *Applied Psychology: Health and Well-Being*, *6*(3), 251-279

8. Andolfi, V. R., Tay, L., Confalonieri, E. & Traficante, D. (2017). Assessing well-being in children: Italian adaptation of the Comprehensive Inventory of Thriving for Children (CIT-Child). *TPM: Testing, Psychometrics, Methodology in Applied Psychology*, *24*(1), 127-145

9. Bandura, A. (1997). *Self-efficacy: The exercise of control*. New York: W. H. Freeman/Times Books/Henry Holt & Co

10. Tahmassian, K. & Moghadam, N. J. (2011). Relationship between self-efficacy and symptoms of anxiety, depression, worry and social avoidance in a normal sample of students. *Iranian journal of psychiatry and behavioral sciences*, *5*(2), 91-98

11. Muris, P. (2002). Relationships between self-efficacy and symptoms of anxiety disorders and depression in a normal adolescent sample. *Personality and Individual Differences*, *32*(2), 337-348

12. Muris, P. (2001). A brief questionnaire for measuring self-efficacy in youths. *Journal of Psychopathology and Behavioral Assessment*, *23*(3), 145-149

13. Roth, J., Sant'Anna, P. H., Bilinski, A. & Poe, J. (2023). What's trending in difference-in-differences? A synthesis of the recent econometrics literature. *Journal of Econometrics*, *235(2)*, 2218-2244

14. Rambachan, A. & Roth, J. (2023). A more credible approach to parallel trends. *Review of Economic Studies*, *90*(5), 2555-2591

15. Andrews, I., Roth, J. & Pakes, A. (2023). Inference for linear conditional moment inequalities. *Review of Economic Studies*, *90*(6), 2763-279
