# Supplementary material for: Longitudinal effect of early social media use on standardized learning outcomes during school career
Source: Nat Hum Behav. 2026 Aug 3;10(10):1953–65. doi: 10.1038/s41562-026-02522-4 (PMC13683038; doi:10.1038/s41562-026-02522-4)
Supplement: Supplementary file 2 [file 41562_2026_2522_MOESM2_ESM.pdf]

nature portfolio

Corresponding author(s): Chiara Respi

Last updated by author(s): May 20, 2026

Reporting Summary

Nature Portfolio wishes to improve the reproducibility of the work that we publish. This form provides structure for consistency and transparency in reporting. For further information on Nature Portfolio policies, see our Editorial Policies and the Editorial Policy Checklist.

Statistics

For all statistical analyses, confirm that the following items are present in the figure legend, table legend, main text, or Methods section.

n/a	Confirmed	
☐	☒	The exact sample size (*n*) for each experimental group/condition, given as a discrete number and unit of measurement
☐	☒	A statement on whether measurements were taken from distinct samples or whether the same sample was measured repeatedly
☐	☒	The statistical test(s) used AND whether they are one- or two-sided<br>*Only common tests should be described solely by name; describe more complex techniques in the Methods section.*
☐	☒	A description of all covariates tested
☐	☒	A description of any assumptions or corrections, such as tests of normality and adjustment for multiple comparisons
☐	☒	A full description of the statistical parameters including central tendency (e.g. means) or other basic estimates (e.g. regression coefficient) AND variation (e.g. standard deviation) or associated estimates of uncertainty (e.g. confidence intervals)
☐	☒	For null hypothesis testing, the test statistic (e.g. *F*, *t*, *r*) with confidence intervals, effect sizes, degrees of freedom and *P* value noted<br>*Give P values as exact values whenever suitable.*
☐	☒	For Bayesian analysis, information on the choice of priors and Markov chain Monte Carlo settings
☐	☒	For hierarchical and complex designs, identification of the appropriate level for tests and full reporting of outcomes
☐	☒	Estimates of effect sizes (e.g. Cohen's *d*, Pearson's *r*), indicating how they were calculated

Our web collection on statistics for biologists contains articles on many of the points above.

Software and code

Policy information about availability of computer code

Data collection	We used Qualtrics to implement our web survey.
Data analysis	STATA, version 15, and R, v 4.5.0 - 2025-04-11 ucrt

For manuscripts utilizing custom algorithms or software that are central to the research but not yet described in published literature, software must be made available to editors and reviewers. We strongly encourage code deposition in a community repository (e.g. GitHub). See the Nature Portfolio guidelines for submitting code & software for further information.

Data

Policy information about availability of data

All manuscripts must include a data availability statement. This statement should provide the following information, where applicable:

- Accession codes, unique identifiers, or web links for publicly available datasets
- A description of any restrictions on data availability
- For clinical datasets or third party data, please ensure that the statement adheres to our policy

As the study relies on administrative data protected by a strict privacy policy, we release the dataset only after an additive stochastic perturbation procedure on test scores, ensuring that the data are fully anonymized. The public dataset is available through the Data Archive for Social Sciences in Italy (DASSI): doi:10.71732/JUJ4BI (https://doi.org/10.71732/JUJ4BI).

Research involving human participants, their data, or biological material

Policy information about studies with human participants or human data. See also policy information about sex, gender (identity/presentation), and sexual orientation and race, ethnicity and racism.

Reporting on sex and gender	Sex was identified through administrative data; respondents gave their consent at the beginning of the interview for all its contents. We used this information as a covariate in the analysis. The term 'sex' was used appropriately in the text. However, given the novelty of the topic and the intense debate around causality, we focused on the causal inference procedure, which is complex and requires detailed explanations. We will address gender and other differences in future publications. These, moreover, would deserve a more in-depth treatment in the theoretical section, which would create significant space constraints.
Reporting on race, ethnicity, or other socially relevant groupings	We used variables that refer to specific groups as covariates: migratory status, family type (e.g. intact-two parent family), highest level of parental educational attainment. This information was collected thorugh the survey. As concerns family educational attainment, we used the dominance criterion (i.e. the highest level of educational attained by the parents) to build the family maximum level of education among parents, as customary in social sciences. Being migrant students in secondary education in Italy still a small minority (in part because of selection in early educational stages) it is impossible to meaningfully distinguish between groups when making the analyses. As concerns family type, we only distinguished between two-parent intact family and the res, because in Italy the family structure is still traditional, and two-parent intact families still represent the norm in the population. Other groups, albeit existent, were not enough big in size to allow a meaningful statistical distinction.<br>We controlled for the potential confounding influence of these variables on the results by including them in the statistical matching process.
Population characteristics	See above.
Recruitment	Participant schools self-selected into our project. Every 10th and 11th grade class of participant school took part in the survey, which was conducted during school hours by trained interviewers. We addressed the external validity of our findings in the comments as well as in the methods section, by showing that the distribution of our student population by school track is highly similar to the overall regional distribution. Therefore, ours is a representative sample by school-type in the sampled provinces.<br>Another source of selection comes from the prevalence by 10th or 11th grade of dropouts and students enrolled in regional courses not covered by the ministerial registries (around 10%-15%). These students are not part of our sample. However, in order to estimate long-term effects of social media use on learning outcomes, the only solution was to use the 10th/11th graders population. We extensively discuss this issue, as well as its implications, in the methods section and in the discussion.
Ethics oversight	The study was approved by the University of Milano-Bicocca's Data Protection Officer and by the University's ethical committee. The details are reported in the paper.

Note that full information on the approval of the study protocol must also be provided in the manuscript.

Field-specific reporting

Please select the one below that is the best fit for your research. If you are not sure, read the appropriate sections before making your selection.

☐ Life sciences ☒ Behavioural & social sciences ☐ Ecological, evolutionary & environmental sciences

For a reference copy of the document with all sections, see nature.com/documents/nr-reporting-summary-flat.pdf

Behavioural & social sciences study design

All studies must disclose on these points even when the disclosure is negative.

Study description	The data used in the study are quantitative and longitudinal.
Research sample	The final respondents' sample is 6109 secondary school students enrolled in grade 10 and 11. See table A1 (appendix) for the representativeness and table 1 for the descriptives of the sample.
Sampling strategy	The sample of schools is self-selected. The participating institutions responded to a call sent to all schools in the sampled provinces. However, within each school, all 10th and 11th grade classes and students participated in the study with no exceptions other than physiological absenses. We accepted all responding schools within our budgetary constraints. Anyway, the sample size is in line or higher compared to the standards in the literature of reference.
Data collection	The web survey was self-administered through the Qualtrics platform in classes. Students used laptops and computers provided by their schools. A trained interviewer was always present during the filling our of the questionnaire, together with a teacher. Students were informed that the study was about the relationship between young people and digital media, but they were blind to the specific research goal of this paper.
Timing	Data collection took place between late October 2023 and early February 2024.

Data exclusions	We excluded a small number of students who were not matched with administrative data as well as multiple grade repeaters, as justified and indicated in the paper.
Non-participation	The only non-participants students were those physically absent on the day of the survey in their class. We had three cases of refusal to complete the questionnaire.
Randomization	Being our analysis of a longitudinal and quasi-experimental nature, we did not use any randomization procedure. We built comparison groups based on the covariates indicated in note 20 in the paper. These variables were used to perform statistical matching.

Reporting for specific materials, systems and methods

We require information from authors about some types of materials, experimental systems and methods used in many studies. Here, indicate whether each material, system or method listed is relevant to your study. If you are not sure if a list item applies to your research, read the appropriate section before selecting a response.

Materials & experimental systems

n/a	Involved in the study	
☒	☐	Antibodies
☒	☐	Eukaryotic cell lines
☒	☐	Palaeontology and archaeology
☒	☐	Animals and other organisms
☒	☐	Clinical data
☒	☐	Dual use research of concern
☒	☐	Plants

Methods

n/a	Involved in the study	
☒	☐	ChIP-seq
☒	☐	Flow cytometry
☒	☐	MRI-based neuroimaging

Plants

Seed stocks	n.a.
Novel plant genotypes	n.a.
Authentication	n.a.
