# Supplementary material for: Longitudinal effect of early social media use on standardized learning outcomes during school career
Source: Nat Hum Behav. 2026 Aug 3;10(10):1953–65. doi: 10.1038/s41562-026-02522-4 (PMC13683038; doi:10.1038/s41562-026-02522-4)
Supplement: Supplementary file 3 [file 41562_2026_2522_MOESM3_ESM.pdf]

nature portfolio

Peer Review File

Longitudinal effect of early social media use on standardized learning outcomes during school career

Corresponding Author: Dr Chiara Respi

Version 0:

Decision Letter:

8th July 2025

Dear Dr Respi,

Thank you once again for your manuscript, entitled "Longitudinal effect of early social media use on standardized learning outcomes during school career," and for your patience during the peer review process.

Your manuscript has now been evaluated by 3 reviewers, whose comments are included at the end of this letter. In the light of their advice, I regret that we cannot offer to publish your manuscript in Nature Human Behaviour.

While the reviewers find your work of some interest, they raise concerns about the appropriateness of the technical approach and the robustness of the data. We feel that these reservations are sufficiently important as to preclude publication of this work in Nature Human Behaviour.

I am sorry that we cannot be more positive on this occasion but hope that you will find our reviewers' comments helpful when preparing your paper for submission elsewhere.

Sincerely,

[redacted]

[redacted]
Nature Human Behaviour

Reviewer expertise:

Reviewer #1: causal inference ; DiD

Reviewer #2: social media and learning outcomes

Reviewer #3: social media and learning outcomes

Reviewers' Comments:

Reviewer #1 (Remarks to the Author):

This study investigates the impact of early social media use on students' academic outcomes over time. The authors combined covariate matching with difference-in-differences, using data from a cohort of students in Italy. Early social media exposure, defined as opening a personal social media account before grade 9, was the intervention, and change in academic performance on standardized tests from grade 5 was the outcome. The authors found that earlier exposure to social media is associated with lower standardized test scores. For example, students who created their first social media accounts in 6th or 7th grade scored significantly lower in their 8th/10th-grade Italian and math tests compared to those who waited longer. Overall, this is a timely and important topic – the question of how early digital media use affects education is of great interest to parents, educators, and policymakers. The manuscript's strengths include its longitudinal design, rigorous analytic methods, and attempt to explore mediating factors. While I appreciate the authors' careful analysis and recognize the value of their findings, I do have several major concerns and suggestions for the author to consider:

Results from descriptive analyses:
1. Figure 2 does not take into account the different ranges in years of different exposures and ignores the cumulative effect of exposure over the years. In particular, Figure 1 shows that the majority of the students opened their first social media accounts between grades 6 and 8, suggesting that the number of years exposed to social media could only range from 0 to 2 for most students in this population. However, the years of exposure to video games can range from 1 to 6. A -0.04 in mathematics for every additional year of exposure to social media translates into up to a -0.08 cumulative effect for most students; on the other hand, a +0.01 in mathematics for every additional year of exposure to video games can translate into as much as a +0.06 cumulative benefit, which has a very similar magnitude as the exposure to social media. Hence, this graph showing only the per-additional-year effect can be misleading and may mask the cumulative effect which is also an important outcome.
2. Why do videogames (and to a lesser extent PCs too) appear beneficial? As explained above, I believe the magnitude of the cumulative effects should be similar between video games and social media, but I'm not sure why their effects are completely in the opposite direction. There are studies suggesting that some of the skills developed through playing certain video games during early ages could help with brain development, but I think it is important to highlight and provide some rationale for this big observation here.
3. This potentially large cumulative effect of video games also made me wonder what the effect would look like if the authors ran a similar DiD design using videogame adoption as the treatment variable. Would the results remain positively statistically significant?
4. Table 1: It is hard to tell if the differences between groups were large enough to show a meaningful difference without something test statistics. Can the authors include a p-value for each treatment-comparison group comparison so that we can know if the differences were significant?

Difference in differences:
1. From Figure 3, early adopters have shown consistently better performance in mathematics and Italian than late adopters, suggesting that there is potential for a decreasing trend over time. Even though the pre-intervention effect is not statistically significant and not comparable in size as the post-intervention findings, many recent studies have cautioned against directly comparing this pre-trend effect to zero due to the low power of this test. I would instead recommend that the authors perform other valid tests like non-inferiority tests on pre-intervention differences to more formally show that any pre-trend divergence was truly negligible. Another robust approach to consider would be to apply a bounding sensitivity analysis such as through HonestDiD.
2. The target estimands seem to differ for different outcomes. Specifically, when looking at the academic outcome for students at grade 8, the comparison group (i.e., those who did not open a social media account until 9th grade or later) had no exposure to social media at all. Hence this comparison essentially compared ever-takers to never-takers. On the other hand, in the comparison looking at the 10th grade outcome, all groups had opened a social media account, so it was essentially studying the association between outcome and the length of time since social media adoption.
3. Can the authors please report confidence intervals for the results shown in Table 2?
4. In footnote 14, the authors note that "If values were missing for only one type of mark (typically, oral tests), the average was imputed with the non-missing value. However, this limitation introduces some noise in the measure." Please clarify the details of the missing value imputation algorithm used. Because the missing percentage was so high, it would be important to address this additional uncertainty and potential bias in the methods.

Reviewer #2 (Remarks to the Author):

I appreciated the opportunity to review this manuscript. It is on an important topic and has some significant strengths. Unfortunately, the results here are so weak they are difficult to interpret. Worse…the authors here falsely interpret weak results as "strong" and "robust" when they are no such thing. This is a common error in this field and one which has caused significant confusion and misinformation. There are also other significant weaknesses to the study I believe would be difficult to correct on revisions such as to give us a clearer picture. I hope that my review is constructive to the authors.

I appreciated that the authors here were honest about debates and conflicts in this field. However, there were still parts where the authors overstate the evidence for various effects. For instance the authors claim: "Concerns are further supported by data showing that as minors' engagement with digital media has increased, mental health has declined in many countries". However, that statement is very much in dispute. For instance, there's no evidence for this in suicide data, which is probably the most reliable data, based on Eurostat and other sources, where youth suicides have remained largely stable or gone down slightly. Similarly, prevalence studies based on DSM criteria have found that, worldwide, anxiety disorders have gone down, not up among youth. Other recent data has cast doubt on other sources…for instance we now know that youth self report is unreliable with a tendency toward false positives (Michael Sheeringa has a recent paper on this and he specifically related this to the social media debate). Further Corredor-Waldron 2024 noted that hospital data is mostly due to the artifact of changes in reporting standards. Scholars should know better than to rely on this kind of ecological fallacy data. Also pop books like Haidt (2024) and Twenge (2017) are not credible sources and should not be cited in a science test.

Minor issue but throughout please use tab indents. It vastly improves readability, which I struggled with with this draft.

The authors assert that there's clear evidence for effects on academics, but that doesn't appear to be true. For instance, most studies of cell-phone bans have found they have little impact on student grades. I think the authors need to dig deeper here to avoid citation bias. Doug Parry's meta-analyses may also be helpful here.

The authors assert "Most studies suggest that intensive social media use is associated with negative well-being outcomes, including poorer sleep quality…" but this isn't true at all. Meta-analyses have consistently found little to no evidence for effects (see Ferguson, Kaye et al., Psychology of Popular Media; Yang & Feng, 2024, as well as Ahmed et al., 2024 for sleep). Once again, the authors exhibit some bias at this point of their literature review, which does call into question some degree of their objectivity on this score.

The study unfortunately has some limitations that are significant. Must of it is based in self-report, which is unreliable. This also subjects the study to some demand characteristics, which can cause some false-positive results.

The main problem with this study is a common one…due to the large sample size, it runs the risk that many "noise" relationships may pop up as "statistically significant", despite not being real effects. Unfortunately, most of the effect sizes reported here are well within this noise threshold (see Ferguson & Heene,, 2022 for discussion and simulations), meaning we can't be confident that they are real. Even if they were real, they are so small that they are unlikely to be clinically meaningful. This has been a common problem in this field where very tiny effect sizes are misinterpreted as hypothesis supportive when they shouldn't be, and then once again miscommunicated to the public both as causal and large, when they are not. This has been a significant source of false information. At best, the authors here can say they have weak and ambiguous findings that are difficult to interpret and are no cause for alarm.

In some analyses, there are overlapping error bars, which also are grounds to avoid overinterpretation.

In some cases the authors seem to wave away null findings, which once again, gives me some pause about their objectivity. They seem over-eager to find "bad news" for smartphones, which again is very common in this field, but is not good science.

The authors appear to find no effect on subjective wellbeing, which seems like an important finding to highlight more than they do.

The discussion is going to need to be completely rewritten. Right now it greatly exaggerates confidence in the hypothesis, despite weak and inconsistent results. The authors claim "robust evidence" and "strong" despite having found nothing of the sort. The indulge in causal language such as "impact" which is entirely inappropriate for a correlational study, and their recommendations section is, once again, based on causal assumptions that are not appropriate.

Overall, I am concerned about the statistical approach. I generally don't like it when authors use what appear to be overly complicated approaches to massage their data, particularly when this is not preregistered. This opens up multiple avenues to p-hacking (to be clear, I'm not making the accusation, just noting this is a widespread problem in this field). I really don't buy this DiD approach as it is used here. Unfortunately, I have no means to check their work as the dataset is unavailable. I understand the authors limitations in using a protected dataset, but unfortunately that's not a rationale for being unable to check the authors' work which I have considerable concerns about.

Ultimately, I just don't see much compelling evidence here…and I worry that the authors seem overeager to overinterpret what appear to be a set of weak and inconsistent results. I wish I could be more positive, and don't mean to be discouraging, and do hope that my comments may give the authors some guidance in their future work.

Standard note: In a couple places, I reference my own work, mainly because I am lazy. The authors should not feel compelled to cite these, and in fact are encouraged to find alternative citations that may provide the same purpose.

Signed,
Chris Ferguson

Reviewer #3 (Remarks to the Author):

I congratulate the authors on this piece of work which will make a meaningful contribution to the field. The manuscript examines the associations between time of social media adoption and academic outcomes in a large dataset of Italian students. The research addresses an important and timely question about the educational consequences of social media adoption among adolescents, employing a quasi-experimental design aimed at discovering the causal relationships. The authors utilise a difference-in-difference analysis to compare students who opened social media accounts at different grade levels and provide compelling insights into the effects of social media adoption on education outcomes. The manuscript demonstrates methodological rigour and tackles a policy-relevant question with significant implications for educational practice. While the study makes a meaningful and important contribution to the understanding of the relationship between social media adoption and academic outcomes, several concerns regarding the strength of causal claims, effect size interpretation, and robustness of findings require careful attention to better align the conclusions with the methodological limitations and evidence presented.

Major comments:

1. Causal inference

The manuscript presents a disconnect between the strong causal language employed throughout the text and the methodological assumptions and limitations of the analyses. This requires substantial revision to more accurately reflect the strength and confidence in causality of the present findings.

The manuscript contains several strong causal assertions, including terms such as "causal interpretation" and "solid causal evidence". Although several confounders are considered, the current presentation lacks clarity regarding the systematic process used to identify and select confounding variables. It remains unclear whether the selection was theoretically driven, pragmatically determined by data availability, or a combination of both. Detailed documentation of this process (e.g., causal diagram) is required to a) support the strong causal claims made, and b) allow readers to evaluate the causal assumptions.

While I appreciate the authors' use of matching algorithms and their testing for robustness across different procedures, several concerns remain. The absence of group differences between grades two and five do not guarantee that the parallel trends

assumption holds throughout the entire study period. Given the limited selection of confounders, as well as the developmental nature of the study population, potential changes in educational policies or practices over time, or evolving socioeconomic conditions that may differentially affect treatment and control groups, it is possible that (time-variant) unobserved variables may confound the present results. While this is not something that can necessarily be addressed in the analyses, it needs to be reflected in a more nuanced presentation of the results and certainty in causality.

2. Robust and homogenous outcomes

The manuscript features a section examining the robustness of the results for different methodological decisions. The resulting statements about the robustness of the findings at times extend beyond the context in which robustness was tested. Specifically, the section ‘robustness checks’ evaluates the robustness of the results to different matching algorithms. However, statements such as “robust empirical evidence regarding the impact of early social media use” overstate this.

Moreover, I would like to see the comparisons between the different grades discussed in the manuscript. Whereas the current writing implies larger effects with earlier introduction, the group comparisons in support of these statements remain non-significant in various instances. (although the trend appears to be consistent). Also, for the additional school-related outcomes, the conclusion that the “effect found on competencies are mirrored on other related academic dimensions” seems to conflict with the mixed nature of the results: several of the additional academic outcomes show no differences across all three grade comparisons. Similarly, the probability of repeating the 9th or 10th grade is increased for students who opened a social media account in the 6th or 7th grade, however, the absence of this relationship for students who opened an account in the 8th grade is not discussed. I would like to see a more nuanced discussion of both the findings that align and conflict with the main interpretation of the present results, which is paramount given the implications of this work.

3. Effect size interpretation

The language used to describe the results tends to overstate the strength of effects. Examples that make claims about the strength of the evidence include “strong, significant and negative impact” or “solid causal evidence”. While the effects are theoretically meaningful, the observed effect sizes would not be considered strong/large by conventional effect size benchmarks.

Minor comments:

4. Mechanistic explanations

The introduction presents two mechanistic explanations that may underlie the potential negative effects of early social media adoption on academic achievement. I agree that the present data do not allow for direct tests of these hypotheses but I wonder whether the differential findings for videogame consoles (and other technological devices in general, where one may suspect similar mechanisms to be at play) could be used to reflect on the mechanisms put forward in the introduction.

5. Type-editing

p6: Given international differences in school systems, referring to grades is most informative for an international readership. For example, “end of lower secondary” (p.6).

p7: Inconsistent terminology of family structure categories. Specifically, referred to as intact and non-intact in the footnote (Footnote 3) but as intact and separated/divorced two-parent family later (p26).

p27: It is stated that written tests tend to be missing but a later section in the footnote suggests that oral tests tend to be missing: “if values were missing for only one type of mark (typically, oral tests)” (Footnote 14).

***

**Following suitable revisions, you may want to consider transferring your manuscript. Although we cannot offer to publish your manuscript, I suggest that you consider Humanities and Social Sciences Communications as a suitable venue for this work. To transfer your manuscript, please use our manuscript transfer portal. You will not have to re-supply manuscript metadata and files, unless you wish to make modifications. For more information, please see our manuscript transfer FAQ page.

Version 1:

Decision Letter:

Dear Dr Respi,

Thank you for your patience while we evaluated your request to reconsider our decision not to take your manuscript forward. Although we appreciate the points you make in your appeal letter, these points address only a small fraction of the issues the reviewers raised, which led to the rejection of your paper. Unfortunately, based on these arguments, we are unable to revisit our

original decision because the extensive and severe methodological and interpretational concerns raised by our reviewers are not addressed in your letter, except in passing.

If you would like us to reconsider our decision, we would require a full point-by-point response to all of the reviewer comments, as well as a revised manuscript. However, please do keep in mind that we cannot make a decision on whether to send your manuscript back to the reviewers before evaluating your full response and revised work.

If you would like to submit a revised manuscript and point-by-point response to each of the reviewer comments, we would hope to receive it within four months. Please let us know if you encounter difficulties with meeting this deadline or would like to withdraw your appeal.

When revising your paper:

- ensure it complies with our format requirements as set out in our Guide to Authors.

- state in a cover note the length of the text, methods and figure legends; the number of references and the number of display items.

Please ensure that all correspondence is marked with your Nature Human Behaviour reference number in the subject line.

Please use the following link to submit your revised manuscript:

Link Redacted

** This url links to your confidential home page and associated information about manuscripts you may have submitted or be reviewing for us. If you wish to forward this email to co-authors, please delete the link to your homepage first **

I look forward to hearing from you soon.

Best regards,

[redacted]

[redacted]
Nature Human Behaviour

Version 2:

Decision Letter:

11th December 2025

Dear Dr Respi,

RE: "Longitudinal effect of early social media use on standardized learning outcomes during school career"

Thank you for submitting your revised manuscript and for all your work on the revision.

Although your manuscript has been revised in response to reviewer comments, it does not fully comply with our editorial policies and formatting requirements.

Before we can send this back to our reviewers, we ask for extensive revisions of the statistical reporting. Specifically, we ask that you report exact p-values (please do not use asterisks to denote statistical significance) and report confidence intervals throughout. The statistical significance threshold should be $p = .05$ - any values higher than that must be interpreted as not statistically significant. We ask that you refrain from describing “trends” or interpreting “marginally” significant results as statistically significant.

Before we can send the manuscript back to our reviewers, we ask that you revise it to ensure that it complies fully with our policies and is formatted according to our requirements. I have attached another copy of our checklist. If you are uncertain as to how to address any of the points in the checklist, please don't hesitate to contact me.

Please use the link below to submit your revised manuscript and related files:

Link Redacted

Note: This URL links to your confidential home page and associated information about manuscripts you may have submitted, or that you are reviewing for us. If you wish to forward this email to co-authors, please delete the link to your homepage.

Thank you in advance for attending to these requests and I look forward to receiving your revised manuscript.

Sincerely,

[redacted]

Nature Human Behaviour

Version 3:

Decision Letter:

15th January 2026

Dear Dr Respi,

RE: "Longitudinal effect of early social media use on standardized learning outcomes during school career"

Thank you for submitting your revised manuscript and for all your work on the revision.

Although your manuscript has been revised in response to reviewer comments, it does not fully comply with our editorial policies and formatting requirements. Specifically, we ask that you include p-values for all results reported in the text. Ideally, you would also include tables for all of the figures in the main text so that full numeric results are reported.

Frequentist inferential statistics should be reported in full wherever they occur (main text, Figure captions, Tables) as follows:

statistic(degrees of freedom) = value, p = value, effect size statistic = value, % Confidence Intervals = values

P values reported should be exact. Do not report 'ns' – give the exact value.

Before we can send the manuscript back to our reviewers, we ask that you revise it to ensure that it complies fully with our policies and is formatted according to our requirements. I have attached another copy of our checklist, as well as the template document that exemplifies our formatting and policy requirements. If you are uncertain as to how to address any of the points in the checklist, please don't hesitate to contact me.

Please use the link below to submit your revised manuscript and related files:

Link Redacted

Note: This URL links to your confidential home page and associated information about manuscripts you may have submitted, or that you are reviewing for us. If you wish to forward this email to co-authors, please delete the link to your homepage.

Thank you in advance for attending to these requests and I look forward to receiving your revised manuscript.

Sincerely,

[redacted]

Nature Human Behaviour

Version 4:

Decision Letter:

Our ref: NATHUMBEHAV-25052488D

23rd April 2026

Dear Dr. Respi,

Thank you for submitting your revised manuscript "Longitudinal effect of early social media use on standardized learning outcomes during school career" (NATHUMBEHAV-25052488D). It has now been seen by the original referees and their comments are below. As you can see, the reviewers find that the paper has improved in revision. We will therefore be happy in principle to publish it in Nature Human Behaviour, pending minor revisions to satisfy the referees' final requests and to comply with our editorial and formatting guidelines.

Please note that we recruited an additional reviewer (Reviewer 4) for this round. Reviewer 4 was invited because it was unclear to what extent Reviewer 2's concerns regarding the strength of the causal inference strategy were shared by other experts in the field. Although we appreciate the issues raised by Reviewer 2, we agree with Reviewer 4 that concerns related to the self-reporting of grades, the informativeness of effect sizes, and the use of causal inference methods with observational data do not preclude

publication of this work in Nature Human Behaviour. However, it is essential that all remaining issues raised by the reviewers—including those from Reviewer 2—are fully addressed in a final revision. This includes expanding the discussion of limitations and responding to concerns about the language used to describe potential mechanisms.

Reviewer 2 also raised a fundamental concern regarding the lack of data availability for peer review. As a condition of acceptance, all data must be made available to the editors and publicly deposited. In addition, fully documented code must be provided.

We are now performing detailed checks on your paper and will send you a checklist detailing our editorial and formatting requirements within two weeks. Please do not upload the final materials and make any revisions until you receive this additional information from us.

Please do not hesitate to contact me if you have any questions.

Sincerely,

[redacted]

[redacted]
Nature Human Behaviour

Reviewer #1 (Remarks to the Author):

The authors have done a great job avoiding overstatement in the revised manuscript, and the new sensitivity analyses has addressed my concerns about the original diff-in-diff analysis. I have no additional concerns in the methods.

In the Discussion, while the authors do acknowledge ongoing debates in the literature, I recommend more explicitly situating this study's findings, with their magnitudes, within the broader spectrum of estimates reported by prior work. I believe the Discussion would benefit from a fuller synthesis supported by an ample set of citations that demonstrates strong coverage of the relevant literature, including the ones the authors provided in the response to reviewers letter, some of which (especially the ones on benchmarking results) should also be elaborated in the main text as well.

Reviewer #2 (Remarks to the Author):

I appreciate these revisions to the authors' manuscript. At the start, I want to say I appreciate the very cordial nature with which they responded to my concerns. At the same time, I was very disappointed with the substance of the revisions themselves, which often were only superficial and dismissive (if, again, cordially so) of my concerns.

As such, most of my concerns remain and, though I think the authors are well-intentioned, their inattentiveness to these issues could only further my questioning of their objectivity, particularly in light of the continued unavailability of the data.

I would refer back to the original review, which remains in force. What follows is merely an addendum, replying to some of the authors rebuttals.

The issue of the mental health claims remains unaddressed. The authors dismiss suicide data, but this is irresponsible to do, as suicide data are the most reliable trackers of mental health. The authors can't simply dismiss this with a few pat references, That's not good science. Further, they fail to note data even from self report that conflicts with their claims. For instance, they refer to US CDC data but, in fact, in recent years the CDC has noted that not only have youth suicides declined in the US, but self-reported mental distress has improved. Similarly, in Australia, serious mental distress has declined from 25% in 2023, to 19% in 2025 (see: https://www.missionaustralia.com.au/what-we-do/evidence-impact-and-advocacy/research/youth-survey/youth-survey-reports/youth-survey-2025/?utm_source=chatgpt.com). Liu et al., 2024 report global *decreases* in youth anxiety over 30 years. Also the authors need to be honest about the limits of self report (see Michael Sheeringa's work in this area).

From this point alone I really encourage the authors to take a moment and consider their (to me, at this point) obvious biases…how that is impact their work, and how that work could potentially misinform others. This is not a personal attack…we are all human, and we all must do this some times. That's what peer review is for, to help you!

Again, the authors are going to have to acknowledge that cellphone bans appear to be failing (see Goodyear et al., 2025 in Lancet). The authors seem to refer to ecological data in other places, so they should also note that, as cellphone bans have proliferated in the US, US standardized testing scores have continued to decline. Please do not keep engaging in minimalization of data you do not like (that's confirmation bias). It's your ethical duty to report all the information, however inconvenient or controversial.

The authors made many claims about the negative impacts of social media across multiple realms, but failed to take seriously my encouragement to include data that would counter that. They, ethically, need to do so, including on "life satisfaction" (which the authors refer to and the Ferguson, Kaye et al., meta does directly address), the Ahmed et al., meta on sleep, as does the systemic review of Ndubisi et al., 2025 which concludes there are only small effects from general use. Most of the papers the authors cite in their rebuttal are just narrative reviews, only Han et al., provide an effect size estimate and it's rather small…and…fails to control for their variables so the actual effect size is likely even smaller. Also, the authors are going to need to make plainly clear in this

paragraph that these are correlations, not causal and it's entirely possible that adolescents who sleep less simply use more screens because they are awake, not that there's some deleterious causal effect that I think is insinuated now (even if that wasn't the authors' intentions).

As to concerns about self-report, again see Michael Sheeringa's work on this. He finds that youth self-report is unreliable, typically in the direction of exaggerating negatives. Also there is work in media psychology that finds that individuals who are higher in psychological problems tend to overreport their screen use. These types of questions, asking participants about use is not only unreliable but also causes false positive via hypothesis guessing. These are all serious Type I error problems for this study. The authors would need to honestly acknowledge them.

The authors are simply incorrect about the interpretation of their effect size. This was actually noted by several reviewers. They seem to have failed to read Ferguson and Heene (2021) which notes how effect sizes in this realm, even from very good studies, tend to be false positive "noise". It's nice the authors chatted among themselves and (presumably) friendly statisticians, but no…this is actually one of the worst problems in social and medical science, leading to considerable public misinformation. I'm well aware of the "foxes guarding the henhouse" effect where you can cite other scholars doing the same thing, but it's bad science there and it's bad science here. The problem for our field is that MOST of our effects are trivial and irrelevant to real life. That has caused a lot of anxiety and rationalization along the "small is big" lines and a lot of unfalsifiable excuses like "well maybe small effects add up over time" or "small effects are big, if you sprinkle them like pixie dust in a big population (which you always can)". This should be obviously self-serving, nonscientific, and greeted with cynicism. We need to improve rigor in our fields not keep misleading people with weak and unreliable results like these. You need to acknowledge your results are inconsistent at best.

The authors seem perplexed that they have null results, but they do. English achievement effects were null (the authors offer a silly rationalization of this, but that reflects obvious confirmation bias). You got results that didn't confirm your hypothesis, end of story. You need to reflect that honestly in your abstract and discussion and not wave it away. Grade 8 results were weak and fragile as the authors note briefly, but mostly breeze by. Effects were generally null for 8th grade adopters. Null results for upper secondary tract, etc. All of these undermine your narrative. Your narrative, as such, must change to acknowledge that your results are weak and inconsistent. You can't claim consistent effects.

Also much of your language remains causal. You can't use correlational results to talk about "effects" (including in the title) or make policy prescriptions. That all must be deleted. Longitudinal data is still correlational and DiD doesn't change that (I'm sorry, despite claims otherwise, it doesn't nothing does). For instance, you just can't say stuff like "First, they emphasize the need for more structured guidelines and digital literacy programs in schools aimed at mitigating the negative impact of social media use among young students." It's irresponsible. That's particularly true given many of your assumptions about your use of DiD are not theoretically justified.

Again, you find no well-being effect. That's a major finding you need to highlight, including in the abstract. Particularly given you refer to it in the introduction when talking about the literature. You can't hide null findings on a major issue under a wall of text when it has substantial policy implications…particularly if you're going to talk about policy.

Again, no your discussion needs to be rewritten almost entirely. Your data is correlational. DiD doesn't change that. There *is no* magical statistical wand that converts correlational data to causal. Every generation has it's new "quick fix" for this…I remember all the promises about SEM, but they fail, particularly given the multiple assumptions, numerous statistical choices, etc., that underly these complex techniques. The more complex the modeling, the further you can be sure you're getting from anything that is actually happening in real life. Point being: you can't take weak, correlational data with effect sizes so small they could reasonably be noise or Type I error and make causal attributions. It's just not good science.

Again, the authors need to make their data public for the review process. That is necessary during peer review to provide independent testing of results. It's amazing this isn't required as a part of submission to top journals like this one. I appreciate the situation the authors may be in, but I'd suggest they wait to publish until they can present the data.

Again, I'll end this by saying that I apologize if I come across as harsh, but there are many, many persistent problems with this manuscript and, in the aggregate, I believe it will do far more to misinform than inform. I hope, this time, the authors will take my concerns more seriously than they did the first round. Please refer back to that review and take the suggestions seriously. Your paper will be far better for it.

Signed,
Chris Ferguson

Reviewer #3 (Remarks to the Author):

I would like to thank the authors for their thorough and thoughtful responses to my comments. The revisions have substantially strengthened the manuscript, and I am satisfied that my concerns have been addressed in sufficient detail. Congratulations on this excellent work.

Reviewer #4 (Remarks to the Author):

Context: I am writing this review as someone who was invited after the first round of reviews had already been completed.

In my view, this is a strong and policy-relevant piece on an important question that has received disproportionate public attention,

but where evidence is relatively scarce. Overall, I think the combination of a large survey linked to achievement records is a strength --- the design is clearly better than the mostly cross-sectional literature in this area. Methodologically, I also think the paper is appropriate: the authors conduct multiple robustness checks, and their discussion of the results is mostly appropriate (see one minor issue below).

One issue of (minor) concern is with the language used in the Mechanisms section. While the section initially uses cautious language about “potential mechanisms” and variables that “may be mediated,” I still feel that more words of caution are needed about this analysis. Retrospective self-reports used to measure treatment timing are probably trustworthy, whereas the variables used in the mechanism analysis seem more diffuse and harder to interpret (e.g., because they concern things that are inherently time variable, like parental oversight). I think the authors should strongly flag this section as “exploratory,” and perhaps even present results in a completely separate table. I also find that the word “mechanism” is perhaps wrong for some of the variables; the use of “mediator” would be better: yes, pervasiveness is arguably a mechanism. But parental control is a deterrent, rather than a mechanism, and it is unclear whether people with higher parental control are any better (e.g., it could be that if your kid is relatively sane with their use, you don’t need to control it very much).

Another ask that I think could add some extra flavor to the paper is for authors to run some simple (exploratory) heterogeneity analysis. It would be interesting, for example, to check if effects differ substantially per parental education or baseline achievement. The results already suggest that early adoption is socially patterned, so it would be nice to have some more results here (e.g., it could be helpful for future work to explore these directions further).

As an aside, I strongly disagree with R2 in many aspects of their review: 1) the fact that the data is based off self-reports is not a deal-breaker, especially given that the students merely self-reported the grade at which they started using social media (this is a reasonable ask, much less so than how many hours do you use a week, for example). 2) The effect sizes seem expressive to me, and the gradually decreasing effects depending on the year of adoption strongly suggest this is not merely noise. 3) The analysis allows authors to draw causal conclusions, given the assumptions of the data at hand --- yeah, it is not an RCT, but it is much better than most cross-sectional work, which dominates this field. 4) I found the discussion appropriate given the findings presented.

Version 5:

Decision Letter:

Dear Dr Respi,

We are pleased to inform you that your Article "Longitudinal effect of early social media use on standardized learning outcomes during school career", has now been accepted for publication in Nature Human Behaviour.

Authors may need to take specific actions to achieve compliance with funder and institutional open access mandates. If your research is supported by a funder that requires immediate open access (e.g. according to Plan S principles or the NIH public access policy) then you should select the gold OA route, and we will direct you to the compliant route where possible. Because authors warrant under our subscription licensing terms that they haven’t committed to licensing any version of their article under a licence inconsistent with the terms of our agreement – including the applicable embargo period – publication under the subscription model isn’t suitable for authors whose funders require no embargo.

Once your manuscript is typeset and you have completed the appropriate grant of rights, you will receive a link to your electronic proof via email with a request to make any corrections within 48 hours. If, when you receive your proof, you cannot meet this deadline, please inform us at rjsproduction@springernature.com immediately. Once your paper has been scheduled for online publication, the Nature press office will be in touch to confirm the details.

Acceptance of your manuscript is conditional on all authors' agreement with our publication policies (see http://www.nature.com/nathumbehav/info/gta). In particular your manuscript must not be published elsewhere and there must be no announcement of the work to any media outlet until the publication date (the day on which it is uploaded onto our web site).

If you have posted a preprint on any preprint server, please ensure that the preprint details are updated with a publication reference, including the DOI and a URL to the published version of the article on the journal website.

An online order form for reprints of your paper is available at https://www.nature.com/reprints/author-reprints.html. All co-authors, authors' institutions and authors' funding agencies can order reprints using the form appropriate to their geographical region.

We welcome the submission of potential cover material (including a short caption of around 40 words) related to your manuscript; suggestions should be sent to Nature Human Behaviour as electronic files (the image should be 300 dpi at 210 x 297 mm in either TIFF or JPEG format). Please note that such pictures should be selected more for their aesthetic appeal than for their scientific

content, and that colour images work better than black and white or grayscale images. Please do not try to design a cover with the Nature Human Behaviour logo etc., and please do not submit composites of images related to your work. I am sure you will understand that we cannot make any promise as to whether any of your suggestions might be selected for the cover of the journal.

You can now use a single sign-on for all your accounts, view the status of all your manuscript submissions and reviews, access usage statistics for your published articles and download a record of your refereeing activity for the Nature journals.

To assist our authors in disseminating their research to the broader community, our SharedIt initiative provides you with a unique shareable link that will allow anyone (with or without a subscription) to read the published article. Recipients of the link with a subscription will also be able to download and print the PDF.

As soon as your article is published, you will receive an automated email with your shareable link.

In approximately 10 business days you will receive an email with a link to choose the appropriate publishing options for your paper and our Author Services team will be in touch regarding any additional information that may be required.

If you have any questions about our publishing options, costs, Open Access requirements, or our legal forms, please contact ASJournals@springernature.com

We look forward to publishing your paper.

With best regards,

[redacted]

[redacted]
Nature Human Behaviour

P.S. Click on the following link if you would like to recommend Nature Human Behaviour to your librarian http://www.nature.com/subscriptions/recommend.html#forms

** Visit the Springer Nature Editorial and Publishing website at www.springernature.com/editorial-and-publishing-jobs for more information about our career opportunities. If you have any questions please click here.**

Open Access This Peer Review File is licensed under a Creative Commons Attribution 4.0 International License, which permits use, sharing, adaptation, distribution and reproduction in any medium or format, as long as you give appropriate credit to the original author(s) and the source, provide a link to the Creative Commons license, and indicate if changes were made.
In cases where reviewers are anonymous, credit should be given to 'Anonymous Referee' and the source.
The images or other third party material in this Peer Review File are included in the article's Creative Commons license, unless indicated otherwise in a credit line to the material. If material is not included in the article's Creative Commons license and your intended use is not permitted by statutory regulation or exceeds the permitted use, you will need to obtain permission directly from

the copyright holder.
To view a copy of this license, visit https://creativecommons.org/licenses/by/4.0/

Reviewers' Comments:

Reviewer #1 (Remarks to the Author):

This study investigates the impact of early social media use on students' academic outcomes over time. The authors combined covariate matching with difference-in-differences, using data from a cohort of students in Italy. Early social media exposure, defined as opening a personal social media account before grade 9, was the intervention, and change in academic performance on standardized tests from grade 5 was the outcome. The authors found that earlier exposure to social media is associated with lower standardized test scores. For example, students who created their first social media accounts in 6th or 7th grade scored significantly lower in their 8th/10th-grade Italian and math tests compared to those who waited longer. Overall, this is a timely and important topic – the question of how early digital media use affects education is of great interest to parents, educators, and policymakers. The manuscript's strengths include its longitudinal design, rigorous analytic methods, and attempt to explore mediating factors. While I appreciate the authors' careful analysis and recognize the value of their findings, I do have several major concerns and suggestions for the author to consider:

Results from descriptive analyses:
1. Figure 2 does not take into account the different ranges in years of different exposures and ignores the cumulative effect of exposure over the years. In particular, Figure 1 shows that the majority of the students opened their first social media accounts between grades 6 and 8, suggesting that the number of years exposed to social media could only range from 0 to 2 for most students in this population. However, the years of exposure to video games can range from 1 to 6. A -0.04 in mathematics for every additional year of exposure to social media translates into up to a -0.08 cumulative effect for most students; on the other hand, a +0.01 in mathematics for every additional year of exposure to video games can translate into as much as a +0.06 cumulative benefit, which has a very similar magnitude as the exposure to social media. Hence, this graph showing only the per-additional-year effect can be misleading and may mask the cumulative effect which is also an important outcome.

> *We thank the reviewer for pointing out this issue. Indeed, such an analysis can be further deepened and made more meaningful. Our intention in including Figures 1 and 2 was simply to present the descriptive data before moving into a more detailed and robust analysis. However, we agree that showing the score for every additional year of exposure can be confusing when comparing different technologies. Since this part is not essential and might cause confusion (as underscored also by other reviewers), we have decided to present only the descriptive correlation between years of social*

media use and learning performance, which we interpret as a good reason to investigate the phenomenon further. This is meant simply to provide an overview of the descriptive patterns in the data. In this way, the paper becomes more coherent, does not introduce other technologies that are not discussed further, and refers specifically to smartphones and social media, since the focus of the study is indeed the use of social media via smartphones.

2. Why do videogames (and to a lesser extent PCs too) appear beneficial? As explained above, I believe the magnitude of the cumulative effects should be similar between video games and social media, but I'm not sure why their effects are completely in the opposite direction. There are studies suggesting that some of the skills developed through playing certain video games during early ages could help with brain development, but I think it is important to highlight and provide some rationale for this big observation here.

3. This potentially large cumulative effect of video games also made me wonder what the effect would look like if the authors ran a similar DiD design using videogame adoption as the treatment variable. Would the results remain positively statistically significant?

We agree with the reviewer that these descriptive data — which we interpret as associations rather than effects — are of limited value if they are not explored in greater depth later in the paper. However, a rigorous analysis of the effect of early videogame use is not feasible with the same level of robustness as for social media. This is because we lack a proper pre-trend for videogame exposure: videogames typically enter children's lives much earlier, leaving us without the temporal variation needed to establish credible pre-trends. Moreover, early videogame use represents a phenomenon that is substantively different from social media adoption.
This observation further strengthens our decision to focus the paper on its core objective: the use of social media experienced through a smartphone.

4. Table 1: It is hard to tell if the differences between groups were large enough to show a meaningful difference without something test statistics. Can the authors include a p-value for each treatment-comparison group comparison so that we can know if the differences were significant?

We agree with the reviewer that p-values help clarify the magnitude of the differences in Table 1. We have therefore added this information.

Difference in differences:
1. From Figure 3, early adopters have shown consistently better performance in mathematics and Italian than late adopters, suggesting that there is potential for a decreasing trend over time. Even though the pre-intervention effect is not statistically significant and not comparable in size as the post-intervention findings, many recent studies have cautioned against directly comparing this pre-trend effect to zero due to the low power of this test. I would instead recommend that the authors perform other valid tests like non-inferiority tests on pre-intervention differences to more formally show that any pre-trend divergence was truly

negligible. Another robust approach to consider would be to apply a bounding sensitivity analysis such as through HonestDiD.

We thank the reviewer for this helpful suggestion to obtain stronger evidence regarding the relevance of our results. We agree that relying solely on standard pre-trend tests may be insufficient, given their low power. In the updated manuscript, we explicitly discuss these limitations and show additional analyses, which we report in the Robustness Checks section. In particular, to go beyond the simple verification of the absence of statistically significant pre-intervention differences, we implement an additional bounding sensitivity analysis following the HonestDiD approach proposed by Rambachan & Roth (2023), as suggested by the reviewer.
The resulting confidence sets are reported in Figures A3, A4, and A5, distinguishing the three comparison groups and the causal effects estimated for grades 8 and 10, for both mathematics and Italian. Each figure presents both the standard OLS confidence sets and the robust confidence sets.
The HonestDiD estimates largely confirm our original interpretation of the results, as well as our cautionary note regarding the comparison between the groups who opened a social media account in grade 8 and those who did so later.

2. The target estimands seem to differ for different outcomes. Specifically, when looking at the academic outcome for students at grade 8, the comparison group (i.e., those who did not open a social media account until 9th grade or later) had no exposure to social media at all. Hence this comparison essentially compared ever-takers to never-takers. On the other hand, in the comparison looking at the 10th grade outcome, all groups had opened a social media account, so it was essentially studying the association between outcome and the length of time since social media adoption.

We fully agree with the reviewer on the point raised. Indeed, there is a difference between Grade 8 and Grade 10, and we already explain this feature of our model in Footnote 21. We believe that this is the correct approach, as our intention is to study the association between the outcome and the time elapsed since the adoption of social media at a specific age. We have further clarified the footnote.

3. Can the authors please report confidence intervals for the results shown in Table 2?

We inserted p-values as suggested for a better readability of the results.

4. In footnote 14, the authors note that “If values were missing for only one type of mark (typically, oral tests), the average was imputed with the non-missing value. However, this limitation introduces some noise in the measure.” Please clarify the details of the missing value imputation algorithm used. Because the missing percentage was so high, it would be important to address this additional uncertainty and potential bias in the methods.

The reviewer raises an important point, namely the measurement issues inherent in marks data. For this reason, we have downplayed the importance of the analyses conducted on this outcome (despite its longitudinal nature) throughout the paper.

Reviewer #2 (Remarks to the Author):

I appreciated the opportunity to review this manuscript. It is on an important topic and has some significant strengths. Unfortunately, the results here are so weak they are difficult to interpret. Worse…the authors here falsely interpret weak results as “strong” and “robust” when they are no such thing. This is a common error in this field and one which has caused significant confusion and misinformation. There are also other significant weaknesses to the study I believe would be difficult to correct on revisions such as to give us a clearer picture. I hope that my review is constructive to the authors.

I appreciated that the authors here were honest about debates and conflicts in this field. However, there were still parts where the authors overstate the evidence for various effects. For instance the authors claim: “Concerns are further supported by data showing that as minors’ engagement with digital media has increased, mental health has declined in many countries”. However, that statement is very much in dispute. For instance, there’s no evidence for this in suicide data, which is probably the most reliable data, based on Eurostat and other sources, where youth suicides have remained largely stable or gone down slightly. Similarly, prevalence studies based on DSM criteria have found that, worldwide, anxiety disorders have gone down, not up among youth. Other recent data has cast doubt on other sources…for instance we now know that youth self report is unreliable with a tendency toward false positives (Michael Sheeringa has a recent paper on this and he specifically related this to the social media debate). Further Corredor-Waldron 2024 noted that hospital data is mostly due to the artifact of changes in reporting standards. Scholars should know better than to rely on this kind of ecological fallacy data. Also pop books like Haidt (2024) and Twenge (2017) are not credible sources and should not be cited in a science test.

> *Thank you for allowing us to clarify how we arrived at the conclusions reported in the literature review. The reviewer challenges our claim that “mental health has declined in many countries.” The reviewer is right to highlight the Eurostat figures showing stable suicide rates in Europe. However, suicide is a delayed and relatively insensitive indicator of short-term changes in mental health. Epidemiological research shows that suicide rates are shaped by multiple social and contextual factors and therefore do not necessarily track short-term or even medium-term fluctuations in depression or anxiety (Hawton & Pirkis, 2017; Turecki & Brent, 2016).*
>
> *While we acknowledge the possibility of false positives in youth self-reports, this limitation, as noted by the reviewer, cannot account for the consistent multi-year trends observed across countries and data sources. The convergence of evidence—from national surveys, hospital records, and school-based studies—strongly supports a real and significant decline in adolescent mental health, particularly since the early 2010s. These patterns are too widespread, sustained, and well-documented to be dismissed as mere artifacts. Key sources such as Bor et al. (2014), Orben et al. (2020), CDC (2024), and, more recently, Blanchflower et al. (2025) report rising psychological distress, depressive symptoms, and self-harm, especially among adolescent girls.*
>
> *However, following the caution suggested by the reviewer, we have slightly softened the sentence by saying that “several mental health indicators have declined in many*

countries." While we thank the reviewer for encouraging us to better reflect nuance, we believe the claim - especially in its new form - remains empirically justified.

Bor, W., Dean, A. J., Najman, J., & Hayatbakhsh, R. (2014). Are child and adolescent mental health problems increasing in the 21st century? A systematic review. Australian & New Zealand journal of psychiatry, 48(7), 606-616.

Hawton, K., & Pirkis, J. (2017). Suicide is a complex problem that requires a range of prevention initiatives and methods of evaluation. The British Journal of Psychiatry, 210(6), 381-383.

Centers for Disease Control and Prevention. Youth Risk Behavior Survey Data Summary & Trends Report: 2013–2023. U.S. Department of Health and Human Services; 2024.

Turecki, G., & Brent, D. A. (2016). Suicide and suicidal behaviour. The Lancet, 387(10024), 1227-1239.

Blanchflower, D. C., & Bryson, A. (2025). Life satisfaction in Western Europe and the gradual vanishing of the U-shape in age. Academia Mental Health and Well-Being, 2(3).

Minor issue but throughout please use tab indents. It vastly improves readability, which I struggled with with this draft.

Thank you for this suggestion, which we which we have incorporated.

The authors assert that there's clear evidence for effects on academics, but that doesn't appear to be true. For instance, most studies of cell-phone bans have found they have little impact on student grades. I think the authors need to dig deeper here to avoid citation bias. Doug Parry's meta-analyses may also be helpful here.

We thank the reviewer, as his comment has helped us clarify the scope and limitations of our research. In this part of the literature review, we refer to the effect of social media use during leisure time on school performance. This type of use is not directly related to cell-phone bans at school for at least two reasons. First, the vast majority of social media use takes place outside the school environment, as our data illustrate (e.g., before falling asleep, while doing homework, etc.). Second, the public policy evaluation literature has often documented cases in which legislative efforts failed to address the negative effects of well-known social causes. For example, even if the literature were 100% clear on the negative effects of social media on academic outcomes, school phone bans might still prove completely ineffective for a variety of reasons (weak implementation, strong student resistance, barriers to enforcement, poor targeting, lack of a clear theory of change, etc.).

The authors assert “Most studies suggest that intensive social media use is associated with negative well-being outcomes, including poorer sleep quality…” but this isn’t true at all. Meta-analyses have consistently found little to no evidence for effects (see Ferguson, Kaye et al., Psychology of Popular Media; Yang & Feng, 2024, as well as Ahmed et al., 2024 for sleep). Once again, the authors exhibit some bias at this point of their literature review, which does call into question some degree of their objectivity on this score.

We believe the reviewer here is too conservative in reading a vastly confirmed result in the literature. While we state that there is ample evidence of a negative relationship, he argues that this is “not true at all.” After re-examining the literature, we can confirm that there is, in fact, broad and convergent agreement on the negative association between social media use (SMU) and sleep problems. To cite only the most recent meta-analyses and reviews:

Han, X., Zhou, E., & Liu, D. (2024). Electronic media use and sleep quality: Updated systematic review and meta-analysis. Journal of Medical Internet Research, 26, e48356.

Yu, D. J., Wing, Y. K., Li, T. M., & Chan, N. Y. (2024). The impact of social media use on sleep and mental health in youth: A scoping review. Current Psychiatry Reports, 26(3), 104–119.

Brautsch, L. A., Lund, L., Andersen, M. M., Jennum, P. J., Folker, A. P., & Andersen, S. (2023). Digital media use and sleep in late adolescence and young adulthood: A systematic review. Sleep Medicine Reviews, 68, 101742.

Alonzo, R., Hussain, J., Stranges, S., & Anderson, K. K. (2021). Interplay between social media use, sleep quality, and mental health in youth: A systematic review. Sleep Medicine Reviews, 56, 101414.

All these studies find a negative relationship between intensive social media use—and digital media use more broadly—and sleep. Regarding the studies cited by the reviewer, Ahmed et al. (2024) is the only one that does not explicitly acknowledge this general relationship, although it does state that problematic social media use (PSMU), which it distinguishes from SMU, is associated with poorer sleep. Thus, even the study cited to support the absence of the relationship ultimately confirms our claim that “intensive social media use is negatively related to sleep.”

Moreover, Ferguson, Kaye et al. (Psychology of Popular Media) is not a study specifically about sleep. We could not find the Yang & Feng (2024) study cited.

In light of this evidence, we maintain that our statement accurately reflects the current state of the literature.

The study unfortunately has some limitations that are significant. Must of it is based in self-report, which is unreliable. This also subjects the study to some demand characteristics, which can cause some false-positive results.

We ask the reviewer to be more specific about his concerns regarding the instrument we used (the survey). Our survey was pre-tested before administration on a sample of students of the same age as those included in the research sample (10 cognitive interviews and 123 completed questionnaires administered to students from academic and technical tracks). Footnote 15 details the rationale for the specific wording we used in our core X variable (social media profile opening). The Y variables come from the Italian National Institute for the Evaluation of the School System.
In addition, most of the scales included in the questionnaire were drawn from validated instruments in the literature and were validated again on our sample before being used in the analyses (see the methodological appendix on measures).
Self-reported age and class of first smartphone acquisition have been used in existing research, with high response rates and evidence that students can easily retrieve and situate this event in time, as it represents a significant moment in their lives:

Dempsey, S., Lyons, S., & McCoy, S. (2019). Later is better: mobile phone ownership and child academic development, evidence from a longitudinal study. Economics of Innovation and New Technology, 28(8), 798–815.
Gerosa, T., & Gui, M. (2023). Earlier smartphone acquisition negatively impacts language proficiency, but only for heavy media users. Results from a longitudinal quasi-experimental study. Social Science Research, 114, 102915.
Gerosa, T., Losi, L., & Gui, M. (2024). The age of the smartphone: An analysis of social predictors of children's age of access and potential consequences over time. Youth & Society, 56(6), 1117–1143.

We better clarified this in the manuscript (methods).

The main problem with this study is a common one…due to the large sample size, it runs the risk that many "noise" relationships may pop up as "statistically significant", despite not being real effects. Unfortunately, most of the effect sizes reported here are well within this noise threshold (see Ferguson & Heene,, 2022 for discussion and simulations), meaning we can't be confident that they are real. Even if they were real, they are so small that they are unlikely to be clinically meaningful. This has been a common problem in this field where very tiny effect sizes are misinterpreted as hypothesis supportive when they shouldn't be, and then once again miscommunicated to the public both as causal and large, when they are not. This has been a significant source of false information. At best, the authors here can say they have weak and ambiguous findings that are difficult to interpret and are no cause for alarm.

In some analyses, there are overlapping error bars, which also are grounds to avoid overinterpretation.

Reviewer 2 and 3 say that our results are weak in magnitude. They state that 0.2 or 0.3 standard deviations in standardized test scores stay within a very small magnitude. We have argued in the paper that 0.2 is not small in magnitude, as it represents ¼ of one of the biggest divides in education and it is roughly equivalent to 6 months of

learning. We detailed it in the manuscript while commenting on the results (the paragraph starting with "The gap in performance…" and footnote 6). We did, though, a careful discussion among the co-authors and external experts, and we confirm our evaluation that effects between .2 and .3 are indeed relevant in educational research. The works by Kraft (2020 and 2023), which is one of the leading scientists in the field (and one of the most cited when it comes to impact estimates on education interventions with standardized achievement outcomes), is clear in stating that an impact of 0.2 standard deviations have to be considered large. Please, note that our dependent variables (i.e. score coming from INVALSI standardized test) come from tests that are built within the OECD-PISA framework, which represents the international benchmark for these measures. We hypothesize that this misunderstanding can be related to the specificity of the field, and the changing nature of empirical benchmarks across fields (and, within the educational literature, across time: empirical benchmarks in the early 2000s were higher indeed). While we understand that effect sizes may be interpreted differently across disciplines, reviewer 2's claim that we have "weak and ambiguous findings that are difficult to interpret and are no cause for alarm" seems really questionable in the light of the references cited above. This said, we tried to use a less emphatic language and rephrase the discussion.

Kraft, M. A. (2020). Interpreting effect sizes of education interventions. Educational researcher, 49(4), 241-253.

Kraft, M. A. (2023). The effect-size benchmark that matters most: Education interventions often fail. Educational Researcher, 52(3), 183-187.

In some cases the authors seem to wave away null findings, which once again, gives me some pause about their objectivity. They seem over-eager to find "bad news" for smartphones, which again is very common in this field, but is not good science.

We re-read the manuscript and we could not find evidence for this claim. As stated above, we de-emphasized some passages in the conclusions.

The authors appear to find no effect on subjective wellbeing, which seems like an important finding to highlight more than they do.

We never discussed "effects" in relation to well-being because we could not estimate any: the variable lacks a longitudinal dimension that would allow us to do so. The inclusion of the well-being variable pertains to a specific section of the paper in which we test one of the empirical mechanisms—among those cited in the literature—through which the impact of social media on academic outcomes might operate. We find that well-being is not associated with math and Italian competencies (Figure A3), but it is negatively correlated with years of exposure to social media (Figure A4). These results have limited causal interpretability, as they rely solely on robust correlations. Therefore, we have been very cautious in presenting them, explicitly referring to them as "statistical associations."

The discussion is going to need to be completely rewritten. Right now it greatly exaggerates confidence in the hypothesis, despite weak and inconsistent results. The authors claim “robust evidence” and “strong” despite having found nothing of the sort. The indulge in causal language such as “impact” which is entirely inappropriate for a correlational study, and their recommendations section is, once again, based on causal assumptions that are not appropriate.

This recommendation is certainly useful in terms of softening the language and avoiding overly strong claims. However, we do not feel we can accept the suggestion that this is merely a correlational study. Our work employs causal inference methods (now also with solid robustness checks) and can therefore be interpreted in causal terms, provided that the assumptions we outline in the limitations section are met. In light of the additional robustness analyses conducted—whose results consistently support our main findings—we believe we can confidently refer to these as meaningful results. Accordingly, we consider it appropriate to use causal language, within the boundaries of the assumptions explicitly stated, as is common practice in the literature employing causal inference techniques.

Overall, I am concerned about the statistical approach. I generally don’t like it when authors use what appear to be overly complicated approaches to massage their data, particularly when this is not preregistered. This opens up multiple avenues to p-hacking (to be clear, I’m not making the accusation, just noting this is a widespread problem in this field). I really don’t buy this DiD approach as it is used here. Unfortunately, I have no means to check their work as the dataset is unavailable. I understand the authors limitations in using a protected dataset, but unfortunately that’s not a rationale for being unable to check the authors’ work which I have considerable concerns about.

We appreciate that the reviewer is not directly suggesting any p-hacking. Unfortunately, we are still awaiting permission to publish the anonymized data. For the time being, what we are able to provide is the full analysis log, which is included in the current submission.
Regarding the concern about an allegedly overly complicated approach, it is important to note that combining Difference-in-Differences methods with statistical matching is a well-established strategy in the social sciences for identifying causal effects in non-experimental settings. This approach has been widely used for at least two decades and is considered a standard tool for addressing selection bias and improving causal inference when randomized experiments are not feasible.

Ultimately, I just don’t see much compelling evidence here…and I worry that the authors seem overeager to overinterpret what appear to be a set of weak and inconsistent results. I wish I could be more positive, and don’t mean to be discouraging, and do hope that my comments may give the authors some guidance in their future work.

We disagree with the reviewer on this point. We hope that the clarifications and revisions we have provided will help make our manuscript clearer and more straightforward.

Standard note: In a couple places, I reference my own work, mainly because I am lazy. The authors should not feel compelled to cite these, and in fact are encouraged to find alternative citations that may provide the same purpose.

Signed,
Chris Ferguson

Reviewer #3 (Remarks to the Author):

I congratulate the authors on this piece of work which will make a meaningful contribution to the field. The manuscript examines the associations between time of social media adoption and academic outcomes in a large dataset of Italian students. The research addresses an important and timely question about the educational consequences of social media adoption among adolescents, employing a quasi-experimental design aimed at discovering the causal relationships. The authors utilise a difference-in-difference analysis to compare students who opened social media accounts at different grade levels and provide compelling insights into the effects of social media adoption on education outcomes. The manuscript demonstrates methodological rigour and tackles a policy-relevant question with significant implications for educational practice. While the study makes a meaningful and important contribution to the understanding of the relationship between social media adoption and academic outcomes, several concerns regarding the strength of causal claims, effect size interpretation, and robustness of findings require careful attention to better align the conclusions with the methodological limitations and evidence presented.

Major comments:

1. Causal inference

The manuscript presents a disconnect between the strong causal language employed throughout the text and the methodological assumptions and limitations of the analyses. This requires substantial revision to more accurately reflect the strength and confidence in causality of the present findings.

The manuscript contains several strong causal assertions, including terms such as “causal interpretation” and “solid causal evidence”. Although several confounders are considered, the current presentation lacks clarity regarding the systematic process used to identify and select confounding variables. It remains unclear whether the selection was theoretically driven, pragmatically determined by data availability, or a combination of both. Detailed documentation of this process (e.g., causal diagram) is required to a) support the strong causal claims made, and b) allow readers to evaluate the causal assumptions.

> *We appreciate this comment. We are unable to provide a causal diagram in this context because there is no sufficiently developed theoretical framework that integrates*

inequalities in both early social media adoption and school achievement. Our strategy, instead, is to include all predictors of achievement or early adoption identified in the literature and available in our dataset, in order to mitigate confounding as much as possible. According to existing research, the strongest predictors of our dependent variables are lagged test scores and family educational and migratory background—both of which we incorporated into our analyses.

We have nevertheless clarified this process in the corresponding section of the manuscript, and we have adopted less assertive language when discussing the results and formulating the conclusions.

While I appreciate the authors' use of matching algorithms and their testing for robustness across different procedures, several concerns remain. The absence of group differences between grades two and five do not guarantee that the parallel trends assumption holds throughout the entire study period. Given the limited selection of confounders, as well as the developmental nature of the study population, potential changes in educational policies or practices over time, or evolving socioeconomic conditions that may differentially affect treatment and control groups, it is possible that (time-variant) unobserved variables may confound the present results. While this is not something that can necessarily be addressed in the analyses, it needs to be reflected in a more nuanced presentation of the results and certainty in causality.

We agree with the reviewer on this point. We have already included an additional variable in the model that can be interpreted as a "bad control," in an attempt to account for potential time-varying factors or unobserved confounders related, for instance, to parental stress or parental control over their children's lives. However, we acknowledge that other sources of bias may still remain. We have made this clearer both in the methodological section and in the conclusions of the manuscript.

2. Robust and homogenous outcomes

The manuscript features a section examining the robustness of the results for different methodological decisions. The resulting statements about the robustness of the findings at times extend beyond the context in which robustness was tested. Specifically, the section 'robustness checks' evaluates the robustness of the results to different matching algorithms. However, statements such as "robust empirical evidence regarding the impact of early social media use" overstate this.

Thanks for the comment, we specified the scope of application of our robustness checks which now include additional analyses making use of an honest diff-in-diff approach.

Moreover, I would like to see the comparisons between the different grades discussed in the manuscript. Whereas the current writing implies larger effects with earlier introduction, the group comparisons in support of these statements remain non-significant in various instances. (although the trend appears to be consistent).

We address this point by including new analyses in which we compare the other groups (i.e. 8th vs 7th grade, 8th vs 6th grade, 7th vs 6th grade). These analyses can be found in figures A1 and A2. In part, this concern was met, indirectly, in Table A2 in the appendix. We thank the reviewer for this comment, since this type of analysis enhances the quality of our conclusions significantly. We included a paragraph on this point and included the related analyses in the appendix.

Also, for the additional school-related outcomes, the conclusion that the “effect found on competencies are mirrored on other related academic dimensions” seems to conflict with the mixed nature of the results: several of the additional academic outcomes show no differences across all three grade comparisons. Similarly, the probability of repeating the 9th or 10th grade is increased for students who opened a social media account in the 6th or 7th grade, however, the absence of this relationship for students who opened an account in the 8th grade is not discussed. I would like to see a more nuanced discussion of both the findings that align and conflict with the main interpretation of the present results, which is paramount given the implications of this work.

Thank you for this comment. We agree that these findings required a more thorough discussion. However, in light of the new robustness checks, the results appear consistent with the idea that the bulk of the effect is associated with opening a social media account in 6th or 7th grade. We agree, though, that the use of the verb “mirrored” entails a more stringent similarity; we corrected the sentence accordingly.

3. Effect size interpretation

The language used to describe the results tends to overstate the strength of effects. Examples that make claims about the strength of the evidence include “strong, significant and negative impact” or “solid causal evidence”. While the effects are theoretically meaningful, the observed effect sizes would not be considered strong/large by conventional effect size benchmarks.

We respectfully disagree with the reviewer on this issue. As highlighted above in our response to Reviewer 2, the educational literature shows broad consensus in interpreting effect sizes around 0.2 as “large effects” (see Kraft 2020 and 2023). Moreover, we have provided several benchmarks against which our results can be meaningfully compared.

Minor comments:

4. Mechanistic explanations

The introduction presents two mechanistic explanations that may underlie the potential negative effects of early social media adoption on academic achievement. I agree that the present data do not allow for direct tests of these hypotheses but I wonder whether the differential findings for videogame consoles (and other technological devices in general, where one may suspect similar mechanisms to be at play) could be used to reflect on the mechanisms put forward in the introduction.

Thank you for the comment. Unfortunately, as noted in the manuscript, we cannot directly test any mechanisms due to the nature of our dataset. Moreover, assessing the impact of videogames would be particularly challenging, given that the vast majority of students already owned a console during elementary school, and we lack measures that would allow us to check for pre-trends. We acknowledge that the section on descriptive statistics may have generated some confusion among the reviewers. Since the paper is already quite dense, we decided to remove that section in order to streamline the manuscript. Nonetheless, the potential influence of other technological devices—including videogames—on our core outcomes has been accounted for in the matching algorithm.

5. Type-editing

p6: Given international differences in school systems, referring to grades is most informative for an international readership. For example, “end of lower secondary” (p.6).

Thank you for noting this issue; it has been fixed when needed.

p7: Inconsistent terminology of family structure categories. Specifically, referred to as intact and non-intact in the footnote (Footnote 3) but as intact and separated/divorced two-parent family later (p26).

We appreciate the reviewer highlighting this issue; it has been addressed in the revised text and tables.

p27: It is stated that written tests tend to be missing but a later section in the footnote suggests that oral tests tend to be missing: “if values were missing for only one type of mark (typically, oral tests)” (Footnote 14).

Thank you for pointing out this issue; it has now been corrected.
